# Supplementary material for: Efficient bubble/precipitate traffic enables stable seawater reduction electrocatalysis at industrial-level current densities
Source: Nat Commun. 2024 Apr 5;15:2950. doi: 10.1038/s41467-024-47121-x (PMC10997793; doi:10.1038/s41467-024-47121-x)
Supplement: Supplementary file 1 — Supplementary Information [file 41467_2024_47121_MOESM1_ESM.pdf]

Supplementary Information

Efficient bubble/precipitate traffic enables stable seawater reduction electrocatalysis at industrial-level current densities

Jie Liang[1,2,5], Zhengwei Cai[1,5], Zixiao Li[2], Yongchao Yao[2], Yongsong Luo[2], Shengjun Sun[1], Dongdong Zheng[1], Qian Liu[3], Xuping Sun[1,2,4*], & Bo Tang[1,5*]

[1]College of Chemistry Chemical Engineering and Materials Science, Shandong Normal University, Jinan 250014, Shandong, China. [2]Institute of Fundamental and Frontier Sciences, University of Electronic Science and Technology of China, Chengdu 610054, Sichuan, China. [3]Institute for Advanced Study, Chengdu University, Chengdu 610106, Sichuan, China. [4]High Altitude Medical Center, West China Hospital, Sichuan University, Chengdu 610041, Sichuan, China. [5]Laoshan Laboratory, Qingdao 266237, Shandong, China. [6]Both authors contributed equally to this work.

[*]Correspondence and requests for materials should be addressed to X.S. (email: xpsun@uestc.edu.cn) or B.T. (email: tangb@sdnu.edu.cn)

This file concludes:

Supplementary Notes 1 to 9

Supplementary Figs. 1 to 72

Supplementary Tables 1 to 8

Supplementary Note 1 | Primary motivations for adopting NCP as the model active catalytic material towards seawater reduction.

Hydrogen evolution reaction, the most classic one in energy electrocatalysis, attains the highest rates on platinum (Pt). Transition metal phosphides (TMPs) are among the best alternatives to Pt (please refer to: *J. Am. Chem. Soc.* **135**, 9267–9270 (2013), *J. Am. Chem. Soc.* **136**, 7587–7590. (2014), *Science* **355**, eaad4998 (2017), *Nat. Nanotechnol.* **14**, 1071–1074 (2019)), and the topotactic synthetic strategies, e.g., a low-temperature thermal phosphidation process, unlock the great opportunities to boost electrocatalytic performances by optimizing the structural features.

In 2014, our group reported an easy, effective, and universal route for 3D CoP nanoarray synthesis, i.e., in situ gas (on-site PH_3 from hypophosphite decomposition)–solid (precursor) reaction (Please refer to: *J. Am. Chem. Soc.* **136**, 7587–7590 (2014)). The resulting CoP on CC is an active TMP-based monolithic electrode for pH-universal HER electrocatalysis. Apart from this work, we successfully fabricated various nanostructured TMPs, e.g., interconnected network of MoP nanoparticles (please refer to: *Adv. Mater.* **26**, 5702–5707 (2014)), Ni_2P nanosheet array (please refer to: *Angew. Chem. Inter. Ed.* **56**, 842–846 (2017)), FeP nanorod array (please refer to: *ACS Catal.* **4**, 4065–4069 (2014)), $Fe_xCo_{1-x}P$ nanowire array (please refer to: *Nano Lett.* **16**, 6617–6621 (2016)), etc., with different morphologies, such as nanowires, nanosheets, and nanoparticles, via our surfactant-free topotactic phosphidation method. Our path-breaking reports usher in the golden era of TMPs as robust and high-active H_2-evolving electrocatalysts (please refer to: *Energy Environ. Sci.* **7**, 3519–3542 (2014), *Adv. Mater.* **28**, 215–230 (2015), *Energy Environ. Sci.* **8**, 3022–3029 (2015), *Adv. Energy Mater.* **5**, 1500985 (2015), *Chem. Soc. Rev.* **44**, 5148–5180 (2015), *Chem. Soc. Rev.* **45**, 1529–1541 (2016), *Adv. Energy Mater.* **6**, 1600087 (2016), *ACS Catal.* **6**, 8069−8097 (2016), *Joule* **2**, 1–18 (2018), *Adv. Mater.* **32**, 1806326 (2019), *Adv. Energy Mater.* **10** 1902666 (2020), *Energy Environ. Sci.* **13**, 3185–3206 (2020), *Adv. Energy Mater.* **10**, 1902104 (2020), *Energy Environ. Sci.* **13**, 4564–4582 (2020), *Angew. Chem. Int. Ed.* **60**, 19550–19571 (2021), *ACS Catal.* **14**, 757–775 (2024), etc.). Moreover, transition-metal

phosphides can serve as promising low-cost HER electrocatalyst candidates for seawater reduction (please refer to: *Catal. Commun.* 162, 106382 (2022)). NiCo phosphides are known as a classical catalyst for hydrogen evolution reaction, and its reactivity origin has been extensively reported in the literature (please refer to: *Nano Lett.* **16**, 7718–7725 (2016), *Adv. Funct. Mater.* **26**, 6785–6796 (2016), *Adv. Mater.* **29**, 1605502 (2017). *Adv. Energy Mater.* **9**, 1901213 (2019), *Appl. Catal. B* **343**, 123579 (2024)). Therefore, we used this synthesis technique again to directly integrate highly active bimetallic NiCo phosphides with the 3D honeycomb-type carbon for the demonstration in this work.

Supplementary Note 2 | Ca^{2+}/Mg^{2+} precipitation.

At present, there is very limited discussion of Ca^{2+}/Mg^{2+} precipitates in electrochemical seawater reduction studies, whereas in practical natural seawater reduction processes precipitation is inevitably occurred and the pH of the electrode/seawater interface rises significantly as a result of the seawater reaction. The change in pH is accompanied by the indirect/direct precipitation of Ca^{2+} as well as direct Mg^{2+} precipitation.

Indirect precipitation of Ca^{2+}:

OH^- (aq) + H_2CO_3 (aq) ↔ HCO_3^- (aq) + H_2O (aq)

OH^- (aq) + HCO_3^- (aq) ↔ CO_3^{2-} (aq) + H_2O (aq)

CO_3^{2-} (aq) + Ca^{2+} (aq) ↔ $CaCO_3$ (s)

Direct precipitation of Ca^{2+}:

OH^- (aq) + Ca^{2+} (aq) ↔ $Ca(OH)_2$ (s)

Direct precipitation of Mg^{2+}:

OH^- (aq) + Mg^{2+} (aq) ↔ $Mg(OH)_2$ (s)

Supplementary Note 3 | More possible reasons why the MBPTS works effectively.

In addition to the theoretical explanations presented by finite element simulations, other possible reasons for the effective operation of the microscopic bubble/precipitate traffic system of our NCP/PC are listed below.

(1) ***Honeycomb-like structure of NCP/PC with hydrophilic, open, well-arranged, low-tortuosity micro-channels***

Unlike NF and GF with disordered pore structures, PC has an ordered honeycomb-like porous structure that allows bubbles to escape more quickly without being hindered during the moving process (please refer to: *Adv. Energy Mater.* **10**, 2002955 (2020)). During the eNSR, the open, well-arranged, low-tortuosity micro-channels of NCP/PC are perfect for the quick transportation of electrons, intermediates, gases, etc. Such aligned and hydrophilic channels within the NCP/PC provide extra vent pathways for electrolytes permeation and gaseous bubble escape, thus facilitating the mass transfer. Moreover, NCP grown almost vertically on PC walls but maintained the PC framework unimpeded, which is believed to facilitate electrolyte diffusion and expedite gas escape. In contrast, bubbles on other NCP-based electrodes stayed for a long time and gathered into a big size before leaving the surface, obstructing the active sites and preventing the electrolyte from diffusing.

(2) ***Larger specific surface areas of the smaller bubbles contribute to better abilities to repel precipitates***

The smaller the sizes of the gas bubbles, the larger specific surface areas of the bubbles, and the more precipitates the bubbles would carry away, hence the best anti-precipitation capacity of NCP/PC.

(3) Hierarchically porous structure of NCP/PC

Benefiting from the low-tortuosity and hierarchically porous structure, the electrolyte can penetrate into pores of the NCP/PC, and the numerous gas bubbles generated on the catalyst surface is favored to be released from the hierarchically porous structures (especially the directional channels), thereby ensuring the

pathways of mass transfer. Notably, apart from the wood channels, mesopores (2–50 nm) may also help H_2 gas release and electrolyte permeation during HER process (please refer to: *Electrochim. Acta* **330**, 135274 (2020)). Furthermore, holes across the wood-derived electrode in the perpendicular direction should be able to connect the parallel/aligned channels, thereby enabling better electrolyte diffusion/ion exchange between the micro-channels as well as reducing the flow resistance/concentration polarization (please refer to: *Energy Storage Mater.* **27**, 327–332 (2020)).

(4) ***Inner burst forces***

As the seawater reduction reaction proceeds, H_2 bubbles in the internal low-tortuosity channels of NCP/PC gradually become more numerous and merge to increase in size, and soon the entire bubble diameter that forms may reach the diameters of the channels. Due to space restriction effect of channels, H_2 bubbles would burst as soon as the diameters approach those of the channels of NCP/PC, generating burst forces. In the interface, the acquired blasting force disperses numerous OH^- (i.e., Ca/Mg precipitates) away from catalytic centers, and thus efficiently avoids the poisoning of active sites (please refer to: *Electrochim. Acta* **330**, 135274 (2020)). Therefore, burst forces derived from the breakage of bubbles within the channeled structure of NCP/PC may contribute to the operation of the bubble/precipitate traffic system.

(5) ***Small stretch forces & Buffer of stress/tension or volume expansion caused by bubble desorption***

According to a study by Liu et al. (please refer to: *Adv. Funct. Mater.* **32**, 2107308, (2022)), internal hollow cavities of NCP/PC should buffer the stress/tension or volume expansion caused by bubble desorption. Except for this reason, smaller bubbles produce smaller stretch forces (please refer to: *Adv. Funct. Mater.* **32**, 2107308, (2022)). For the NCP/PC, the smallest stretch force induced by the smallest bubbles will minimize the damage of the electrode. Thus, both the two reasons guarantee the longer lifespan of NCP/PC during seawater oxidation process.

(6) The least amount of inactive areas

Since NCP/PC exhibits the best seawater reduction performance, the nucleation and growth of bubbles on its surface would result in the least amount of inactive areas separating the seawater from the active sites (please refer to: *Chinese Chem. Lett.* **35**, 108351, (2024), *J. Am. Chem. Soc.* **142**, 1857–1863, (2020), *Appl. Catal. B* **286**, 119920 (2021)). Bubbles that grow on the electrode over a long period of time can contribute to more inactive areas separating the seawater and the catalytic surface. The smaller bubble diameter means lower gas-phase occupancy and higher exposure rate of active areas, which indicates higher HER activity (please refer to: *Appl. Energy*, **307**, 118278 (2022)).

(7) Less gas transfer resistance

In situ microscopic observation was performed to directly showcase bubble evolution behaviors for different NCP-based anodes at the same electrolysis voltage. The large numbers of generated gas bubbles of NCP/PC may result from the higher ECSA that generates more catalytic sites. Moreover, the larger quantity of bubbles and the smaller bubble sizes for our NCP/PC indicate less gas transfer resistance for the NCP/PC (please refer to: *Nano Lett.* **23**, 629–636 (2023)), which facilitates the operation of microscopic bubble/precipitate traffic system. According to the research on the relationship between bubble release and local electrolyte potential, large bubbles will greatly disturb the local electrolyte potential and seriously hinder the subsequent bubble generation (please refer to: *Nano Lett.* **23**, 629–636 (2023)). Therefore, the small bubbles of NCP/PC may make the local electrolyte potential less affected, leading to increased energy utilization efficiency (please refer to: *Nano Lett.* **23**, 629–636 (2023)). Additionally, bubbles with smaller sizes are better at facilitating charge transfer and solution resistance (please refer to: *Adv. Funct. Mater.* **32**, 2107308, (2022)), which lowers the potential during gas-evolving electrocatalytic reactions.

In conclusion, using just low-cost natural pine wood blocks as the electrode precursor, it is now possible to create self-cleaning eNSR electrodes. Importantly, smallest bubbles, more directional movements of bubble flows, fewer vortices,

higher sensitivity of gas-liquid interface to perturbation, the least amount of inactive/dead areas, less gas transfer resistance, and less affected local electrolyte potential, etc., may all allow PC-based electrodes like NCP/PC to achieve the more effective microscopic bubble/precipitate transport system.

Supplementary Note 4 | Advantages of this work over previously reported work in terms of anti-precipitation seawater electrolysis.

One of the most primary obstacles to the direct electroreduction of natural seawater is the continuous formation of insoluble Mg^{2+}/Ca^{2+} precipitates on the cathodic electrode, particularly when the operating current densities are high enough (please refer to: *Nat. Energy* **5**, 367–377 (2020)). Moreover, even when using alkaline seawater, the residual Mg^{2+} and Ca^{2+} will still continue to accumulate and form tiny precipitates on the surface of the catalyst over a long period of time, ultimately leading to degradation of performance. Therefore, if seawater needs to be used as electrolytes for generation of H_2, the ways to repel Mg^{2+}/Ca^{2+} precipitates should be discovered. A study by Zeng et al. focused on improving the catalyst-substrate interaction for longer-term stability in eNSR (please refer to: *J. Mater. Chem. A* **7**, 25628–25640(2019)). Anchoring single-atom Pt on CoP also outperformed the Pt/C counterpart for long-term eNSR (please refer to: *J. Mater. Chem. A*, **8**, 11246–11254 (2020)). A core–shell NiMo@C_3N_5/glassy carbon electrode also showed better eNSR stability than Pt/C (please refer to: *Chem. Eng. J.* **438**, 135379 (2022)). Although the catalysts showed HER activities in natural seawater, the stability is not ideal due to the problem of precipitation of Mg^{2+}/Ca^{2+}. For example, while the NiMo wrapped by C_3N_5 shells can be protected from the poison of impurities, white insoluble salts were constantly deposited on the NiMo@C_3N_5/glassy carbon electrode (please refer to: *Chem. Eng. J.* **438**, 135379 (2022)).

To the best of our knowledge, no HER electrode shows particularly high stability in natural seawater. In stability measurements, the previously reported HER electrodes generally displayed current densities below 100 mA cm^{-2} and electrolysis periods under 100 h (please refer to: *J. Mater. Chem. A* **7**, 25628–25640 (2019), *J. Mater. Chem. A* **8**, 11246–11254 (2020), *Chem. Eng. J.* **438**, 135379 (2022), *Adv. Energy Mater.* **9**, 1901333 (2019), *Appl. Catal. B* **304**, 120993 (2022), *J. Electroanal. Chem.* **916**, 116379 (2022), *Green Chem.* **23**, 4551–4559 (2021), *J. Mater. Chem. A* **8**, 25768–25779 (2020), *ACS Energy Lett.* **5**, 2681–2689 (2020), *Energy Environ. Sci.* **10** 788–798(2017), *J. Energy Chem.* **55** 92–101 (2021), *ACS Nano*, **12**, 12761–12769 (2018), *Nat. Commun.*

13, 5785 (2022)). While these studies proposed various catalysts capable of electrolysis in natural seawater, few of them targeted the design of precipitation-repelling catalysts. Recently, a novel electrode design for precipitation-free natural seawater reduction that relies on a special local surface micro-environment was recently reported by Guo et al. (please refer to: *Nat. Energy* **8**, 264–272 (2023)). They proposed that the strong binding of OH^- on Cr_2O_3 would restrict the generated OH^- within the electrical double layer. Although this work captured scientific novelty, the electrode's capacity for preventing precipitate formation was limited, with a precipitation-free electrolysis demonstration at the current density of 100 mA cm^{-2} for 2 h. The emphasis of the work is on achieving anti-precipitation reduction of natural seawater, which was truly difficult to achieve in previous studies (please refer to one of best work so far: *Nat. Energy* **8**, 264–272 (2023)). High-speed camera videos, in situ Raman data, ex situ characterization data, and theoretical simulations all confirm the strong precipitation resistance of our electrode and the effectiveness of our strategy. Our work not only demonstrates uniquely constructed 3D cathodes with record-high natural seawater reduction performances but also reveals universal principles underlying superb anti-precipitation performances (i.e., the PC-based MBPTS design) to solve the long-standing issues in seawater electroreduction, which provides vital insights for electrode designs toward industry electrolysis of seawater.

Supplementary Note 5 | Methods of simulating bubble rising speed outside the electrode.

We used Hadamard-Rybczynski small spherical bubbles model based on pressure-drag balance to calculate bubble rising speed outside the electrode. The compressibility of the liquid phase is ignored in the continuity equation and the influence of gas phase is ignored in the momentum equation in this model:

$$\rho_l \nabla \cdot u_l = 0 \tag{1}$$

$$\frac{\partial \rho_g \phi_g}{\partial t} + \nabla \cdot \left(\rho_g \phi_g u_g\right) = 0 \tag{2}$$

$$\rho_l \left(\frac{\partial u_l}{\partial t} + (u_l \cdot \nabla) u_l\right) = \nabla \cdot \left(-pI + \mu_l(\nabla u_l + (\nabla u_l)^T)\right) + \rho_l g \phi_l \tag{3}$$

where the corner marks l and g refer to liquid phase and the gas phase, and u, p, and ϕ are the velocity field, pressure field and phase field. ρ and μ are the density and viscosity, respectively. To close Supplementary equations (1)–(3), the pressure-drag balance model is required:

$$\phi_l \nabla p = -\frac{12\mu_l}{\delta^2}\left(u_\mathrm{g} - u_l\right) \tag{4}$$

where δ is the bubble diameter, and $u_g - u_l$ is the so-called bubble slip velocity. The compute domain is shown in Supplementary Fig. 55. The red boundary is electrode surface (gas inlet surfaces), a homogenous gas flux satisfies:

$$\left(\rho_g \phi_g u_g\right) \cdot n = \frac{22.4\,\mathrm{mol}/L \cdot i_0}{F \rho_g S} \tag{5}$$

where n is the surface normal basis, i_0 is the total input current of the electrode, F is the Faraday constant, S is the surface area. The green boundary is the water surface (gas outlet), satisfies

$$\left(-pI + \mu_l(\nabla u_l + (\nabla u_l)^T)\right) \cdot n = 0 \tag{6a}$$

$$\phi_g = 0 \tag{6b}$$

The blue boundary is the symmetry plane and other boundaries (far enough to the electrode) are slip walls. Comsol Multiphysics software based on finditer element method is used to solve the equations above.

Supplementary Note 6 | Methods of simulating bubbles repelling the precipitates in the electrode.

Volume-of-fluid (VOF) multiphase fluid model based on level set equation is used in this simulation. Precipitation is seen as a solute control by convection-diffusion equation coupled with VOF model.

The mixed phase satisfies incompressible Navier-Stokes equations (NSEs) (1) and (3), but the density of fluid (ρ_l) and viscosity of fluid (μ_l) in NSEs (1) and (3) should be replaced to:

$$\rho = \rho_l + (\rho_g - \rho_l)\phi_g \tag{7a}$$

$$\mu = \mu_l + (\mu_g - \mu_l)\phi_g \tag{7b}$$

And the inertia term can be ignored in this scale. That is

$$\rho\nabla \cdot u = 0 \tag{8}$$

$$\rho\left(\frac{\partial u}{\partial t} + (u \cdot \nabla)u\right) = \nabla \cdot \left(-pI + \mu(\nabla u + (\nabla u)^T)\right) \tag{9}$$

And the mixed phase velocity u (instead of u_l) can also be expressed in $u = u_l + (u_g - u_l)\phi_g$.

Level set equation is used to simulate the volume fraction of gas ϕ_g, which satisfies

$$\frac{\partial \phi_g}{\partial t} + u \cdot \nabla\phi_g = \gamma\nabla \cdot \left(\epsilon\nabla\phi_g - \phi_g(1 - \phi_g)n_{\nabla\phi_g}\right) \tag{10}$$

where $\gamma = 10^{-6}\, m\, s^{-1}$ and ϵ takes a half of the local mesh cell scale in the simulation. $n_{\nabla\phi_g}$ is the unit vector along the direction of $\nabla\phi_g$.

The concentration of precipitation c in the water satisfies

$$\frac{\partial c}{\partial t} - D\nabla^2 c + u \cdot \nabla c = 0 \tag{11}$$

where D is the isotropic diffusion coefficient of the precipitation, which is related to the precipitation particle size. $D = 10^{-11}$ $m^2\ s^{-1}$ is set in the simulation.

Supplementary equations (8)–(11) are the governing equations of this model. Different boundary conditions are investigated. Liquid inlets, opened boundaries and symmetry planes are marked in the blue, green lines and yellow lines, respectively, in Supplementary Fig. 56, and other boundaries are electrode surfaces. Outside all opened

boundaries, $c = 0$. In Supplementary Fig. 56a,b, gas inlets velocity at electrode surfaces is $v_0 = 10^{-6}$ m/s. Liquid inlets are evenly divided into thirds in the horizontal direction, the middle part (marked in blue) applies a larger liquid inlet velocity $2v_1$ while the liquid inlet on the sides applies a smaller liquid inlet velocity $0.5v_1$ to simulate the inhomogeneity of fluid flow inside the electrode. Where

$$v_1 = \frac{22.4\,\mathrm{mol}/L \cdot i_0}{F\,V} \tag{12}$$

V is the volume of electrode in the compute domain. c of the inlet liquid is the initial concentration c_0. In Supplementary Fig. 56c, the bottom boundary is a symmetry plane. The electrode surfaces have a gas inlets velocity.

Supplementary Note 7 | Symmetric natural seawater electrolysis.

In fact, any feeding mode (either asymmetric one-side feed or symmetric two-side feeds) can be adopted for seawater electrolysis (please refer to: *Mater. Today* **69**, 193–235 (2023), *Nat. Energy 5*, 367–377 (2020)). In the field of seawater electrolysis, there are also notable advances in symmetric electrolysis (please refer to: *Nat. Commun.* **14**, 3607 (2023), *Nat. Energy* **8**, 264–272 (2023)), of which the use of natural seawater on both sides are less because when untreated natural seawater is supplied as the electrolyte to both sides, the anode would suffer from severe chlorine-based corrosion, while the cathode surface would be physically covered by severe Mg^{2+}/Ca^{2+}-based precipitation (i.e., more dead/blocked reaction sites). Moreover, asymmetric electrolysis can avoid one of these situations directly to a certain extent (please refer to: *Energy Environ. Sci.* **13**, 1725–1729 (2020), *Adv. Mater.* **33**, 2101425 (2021), *Small* **19**, 2208076 (2023)). Different feeding modes have the own advantages, and it is necessary to adopt a suitable feeding method according to a specific testing situation, performance/cost requirements, or the electrolyzer components, etc.

Supplementary Note 8 | Rough estimations of electricity cost per kilogram of H_2 from the electrochemical data achieved by our flow-type electrolyzer.

(i) Efficiency

Electrolyzer power = 3.38 V × 0.5 A cm^{-2} = 1.69 W cm^{-2}

The lower heating value (LHV) of H_2 is applied to calculate the efficiencies of NCP/PC electrocatalyst. LHV = 120 kJ g^{-1}.

The H_2 production rate at 0.5 A cm^{-2} is 2.59 × 10^{-6} mol H_2 cm^{-2} s^{-1}

H_2 power out = H_2 production rate × LHV = 0.625 W cm^{-2}

Efficiency = H_2 power out / Electrolyzer power × 100%= 37.0%.

(ii) Electricity cost of hydrogen production

Mass of produced H_2 = H_2 production rate × Electrolyzer area × Molar mass H_2 × Time = 0.744 g H_2.

Volume of H_2 = m / ρ=0.774g / (0.09 g $liter^{-1}$) = 8.6 liters

Energy consumption = Electrolyzer power / (H_2 production rate × Molar mass H_2) = 90.6 kW h/kg H_2.

Electricity cost (H_2/kg) = Energy consumption Electricity bill = 90.6 kW h/kg H_2 × \$ 0.02/kW h=\$ 1.81 /kg H_2.

Supplementary Note 9 | Whether the TMPs experience reconstruction in cathodic hydrogen evolution reaction?

It is a well-established truth that the water reduction (i.e., the HER catalysis) process will reconstruct the metal phosphides (please refer to: *ACS Catal.* **7**, 103–109 (2017), *Energy Environ. Sci.* **15**, 727–739 (2022), *Small* **14**, 1800421 (2018)). This means that all metal phosphides are the pre-electrocatalysts. We give some recent examples here to expound the common phenomenon of such reconstruction, which often does not affect the HER activity much.

In a recent work reported by Li et al., CoOOH and FeOOH both generated in situ on S, Fe dual-doped CoP nanoneedle array@Fe foam after 100 h of HER stability test at −1000 mA cm^{-2} (please refer to: *Chem. Eng. J.* **470**, 144081 (2023)). In Pan's recent work, $Ni(OH)_2$ and FeOOH generated in situ on Ni_2P/FeP-Fe foam after 50 h of HER stability test at −100 mA cm^{-2}, and $Ni_2P/Ni(OH)_2$ heterojunction was determined as the active phase (please refer to: *Adv. Funct. Mater.* **33**, 2302621 (2023)). Notably, Zhang et al. reported that XPS peaks in the Ni 2*p* region shifted to lower binding energy after 80 h of HER stability test at −100 mA cm^{-2}, with the reduction of Ni^{3+} to Ni^{2+} (please refer to: *Appl. Catal. B* **298**, 120611 (2023)).

In other words, based on these studies, it seems that the Fe-group elements (Fe, Co, and Ni)-based phosphides may generate the corresponding high-valence metal hydroxides and/or oxyhydroxides after a long time of HER operation. However, *operando* measurements like *operando* X-ray absorption spectroscopy (XAS) and *operando* Raman experiments provide more information. In Patzke's work (please refer to: *Energy Environ. Sci.* **15**, 727–739 (2022)), *operando* results demonstrate that cobalt phosphide nanoboxes (Co–P NBs) and Fe-doped cobalt phosphide nanoboxes (Co@CoFe–P) both underwent the reconstruction process after the HER tests with newly generated configurations (P–Co–O for Co–P NBs and P–Co–O–Fe–P for Co@CoFe–P). The reconstructed P–Co–O and P–Co–O–Fe–P moieties with low-valence metal centers (M^0/M^+) were the true HER active sites. Moreover, the average valence state of Co ions for the Co–P NBs attained the lowest value of +1.28 at negative

-0.13 V_{RHE}. Interestingly, when no voltage is applied after the HER electrolysis, such a decrease of the average valence state of Co under electrolysis conditions turns into an increase of the average valence state, and the final average valence state is even slightly higher than that of the pristine Co-P NBs. Therefore, reconstruction will occur for TMPs during the HER, and the valence change of the metal center is dynamic and complex (The valence of the metal center of a phosphide may decline during electrolysis, but it may rise to a higher state than its initial state after ending electrolysis process). In addition, such reconstruction during HER may not negatively affect the HER activity of the TMPs.

Moreover, we point out that the reconstruction/phase transition of metal phosphides in some reports was not significant, usually due to small HER current densities (e.g., Negligible changes occurred on $Co_{0.42}Fe_{0.58}P$@C after 18 h of HER stability test at −8 mA cm^{-2}, please refer to: *Adv. Energy Mater.* **12**, 2202394 (2023), negligible changes occurred on OMS-Ni1-CoP after 15 h of HER stability test at −10 mA cm^{-2}, please refer to: *Appl. Catal. B.* **316**, 121667 (2022), negligible changes occurred on Er-NiCoP/NF after 48 h of HER stability test at −20 mA cm^{-2}, please refer to: *Carbon Energy.* **5**, e217 (2023)). For a more example with longer electrolysis time period, 80 h of chronoamperometric testing biased at −10 mA cm^{-2} just resulted in slight surface oxidation of NiCoP@Mxene, due to the low operation current density (please refer to: *ACS. Nano.* **16**, 11049–11058 (2022)). Therefore, the low current density generally does not significantly change the metal phosphide phase.

(1) Importantly, for NCP-based electrodes, amorphous species may generate in situ during the HER process, which generally can act as new active sites and do not have a negative effect on the HER performance (please refer to: *Appl. Catal. B* **339**, 123136 (2023), *Adv. Funct. Mater.* **33**, 2205161 (2023)).

(2) Post-test phosphides typically form high valence metal species and have less phosphorus bonded to the metal (i.e., rapid leaching of P atoms during the reconstruction process), which can be obtained from the Raman results like vibrational modes of M-OH (please refer to: *Chem. Eng. J.* **473**, 145397 (2023)), XRS results like binding energy shift and changed peak intensity (please refer to:

Chem. Eng. J. **473**, 145397 (2023), *Appl. Catal. B* **339**, 123136 (2023), *Energy Environ. Sci.* **15**, 727–739 (2022)), HRTEM results, and elemental mapping results (please refer to: *Chem. Eng. J.* **473**, 145397 (2023)).

(3) XPS peaks in the P 2*p* region usually show decreased intensities (please refer to: *Energy Environ. Sci.* **15**, 727–739 (2022), *Nano Energy* **115**, 108979 (2023)). The P–O peak usually negatively shift, which may due to the formation of low-valence P species (please refer to: *Chem. Eng. J.* **473**, 145397 (2023), *Adv. Funct. Mater.* **33**, 2300625, (2023)). Generally, the singles in the P 2*p* region can be deconvoluted into peaks that belong to phosphorous oxide (P_xO_y), phosphates, and metal–P bonds. Surface-derived phosphates do not negatively affect the HER performance much; instead, such phosphates can behave as multifunctional phase that (i) can prevent internal metal phosphide from further leaching and (ii) may accommodate more electrons to facilitate the HER process (please refer to: *Adv. Mater.* **34**, 2107548 (2022)).

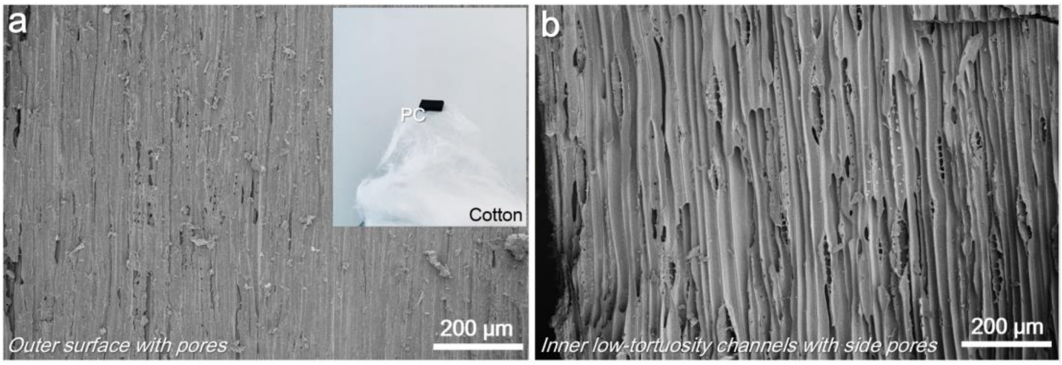

Supplementary Fig. 1 | SEM images for the bare PC. (a) Outer surface of PC with side pores. The surface contains some small debris. The inset image shows a small piece of free-standing PC resting stably on a lump of sterile cotton. (b) Inner low-tortuosity channels with side pores for the PC.

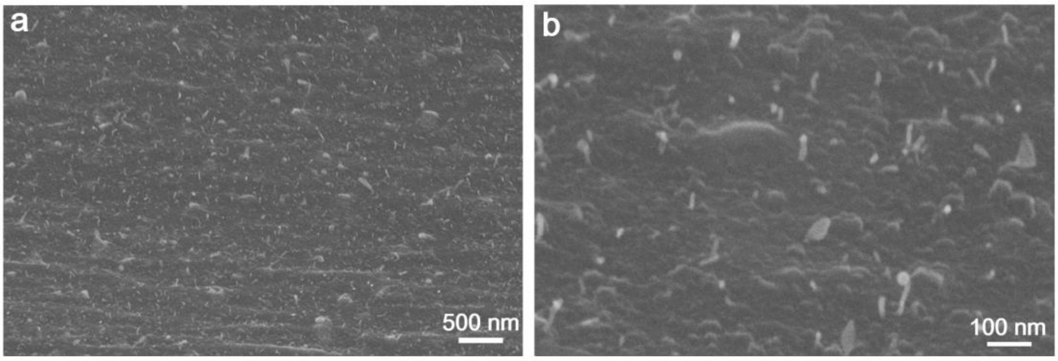

Supplementary Fig. 2 | (a,b) More high-magnification SEM images of PC.

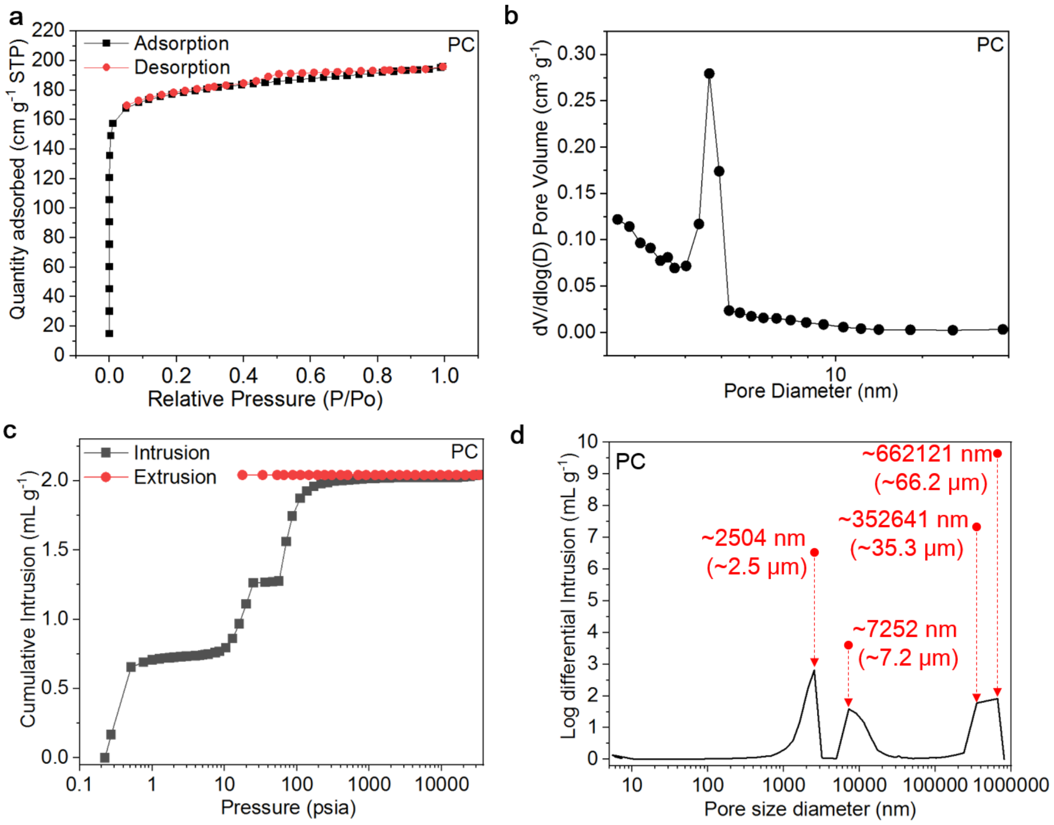

Supplementary Fig. 3 | Basic pore structure analyses of PC. (a) Nitrogen adsorption-desorption isotherms and (b) the corresponding pore size distribution curve for the PC. (c) Mercury feeding and mercury withdrawal curves and (d) MIP pore size distribution curve for the PC.

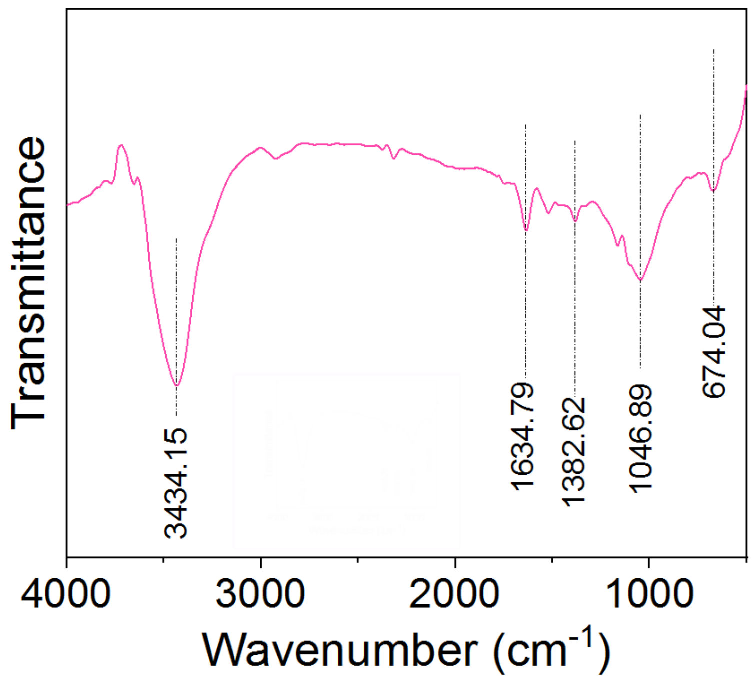

Supplementary Fig. 4 | Infrared signals of the PC in the range of 500–4000 cm^{-1} obtained from Fourier transform infrared spectroscopy measurement. The result suggests the stretching vibration of –OH (3434.15 cm^{-1}), bending vibration of absorbed water molecules (1634.79 cm^{-1}), H−O deformation vibration (1382.62 cm^{-1}), and existence of H−O (1046.89 cm^{-1}), etc., in the PC sample (please refer to: *Energy Fuels* **35**, 18815–18823 (2021), *J. Colloid Interf. Sci.* **608**, 70–78 (2022), *Nat. Commun.* **14**, 6024 (2023), *Compos. B Eng.* **224**, 109169 (2021), *J. Alloys Compd.* **947**, 169446 (2023)).

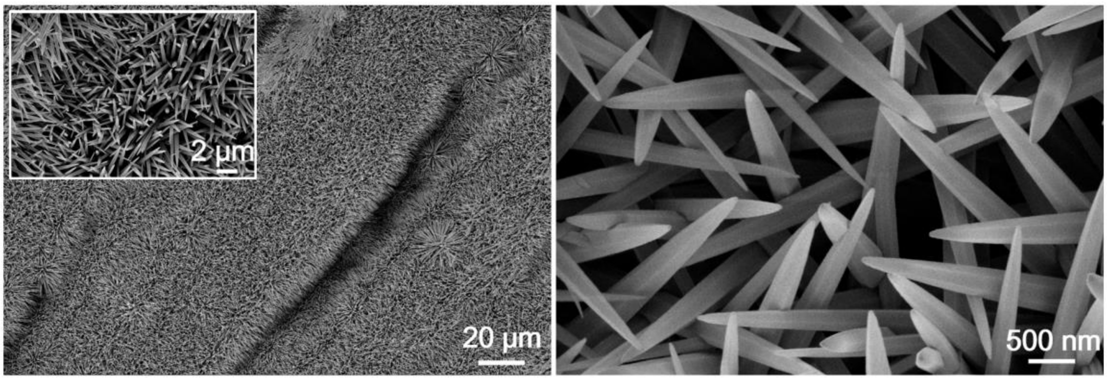

Supplementary Fig. 5 | SEM images for the precursor.

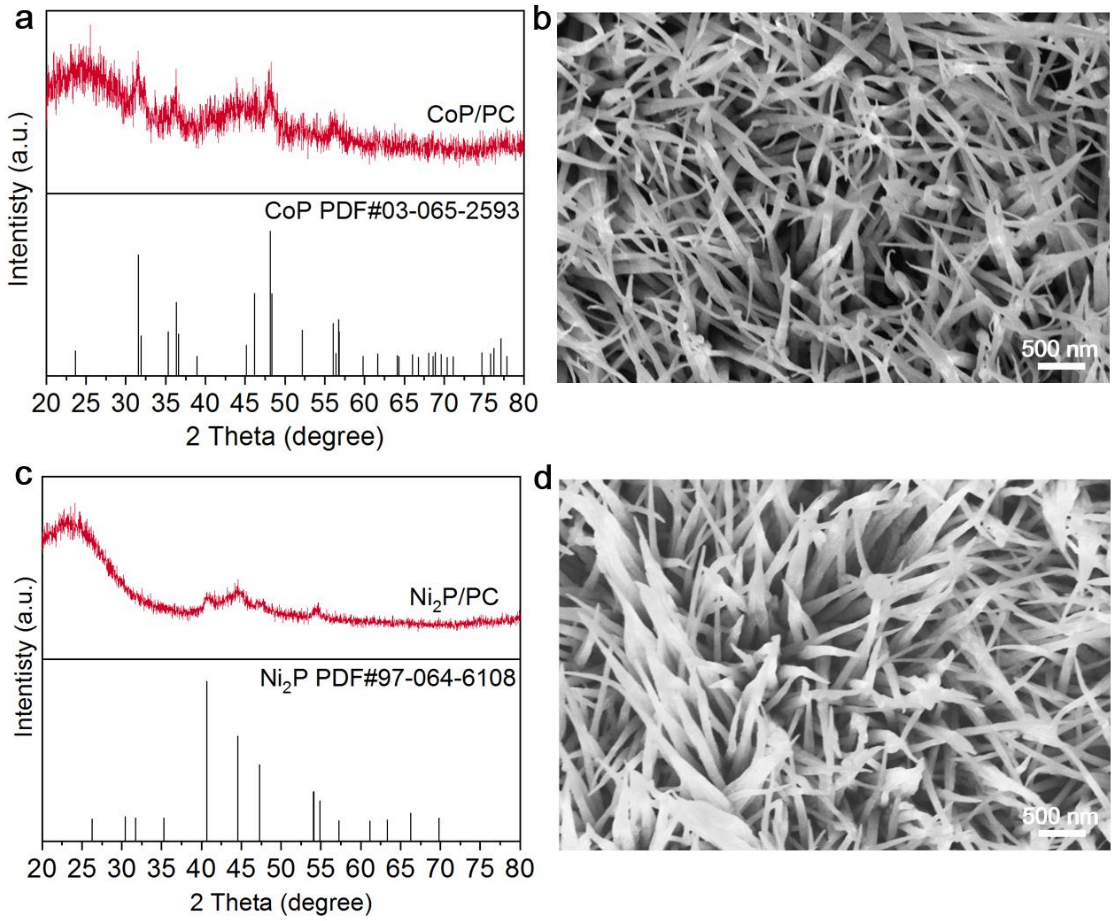

Supplementary Fig. 6 | XRD patterns and high-magnification SEM images. (a) XRD pattern and (b) SEM image for the CoP/PC. (c) XRD pattern and (d) SEM image for the Ni_2P/PC.

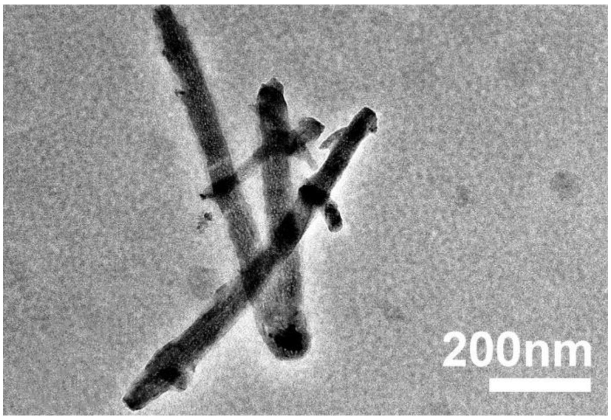

Supplementary Fig. 7 | TEM image of NCP.

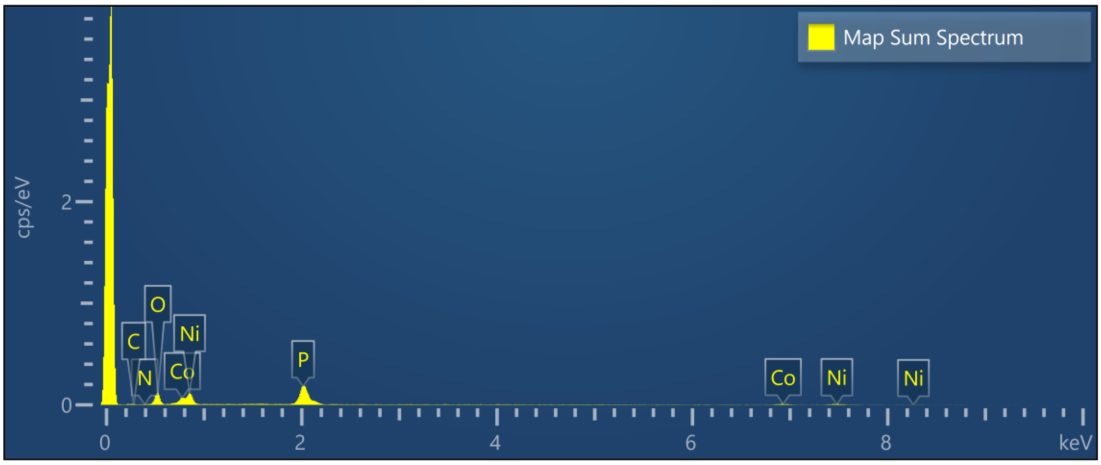

Supplementary Fig. 8 | EDX spectrum of NCP/PC.

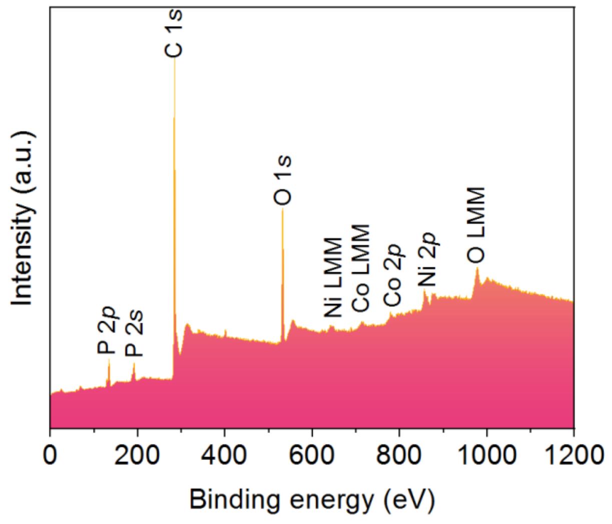

Supplementary Fig. 9 | XPS survey spectra of NCP/PC.

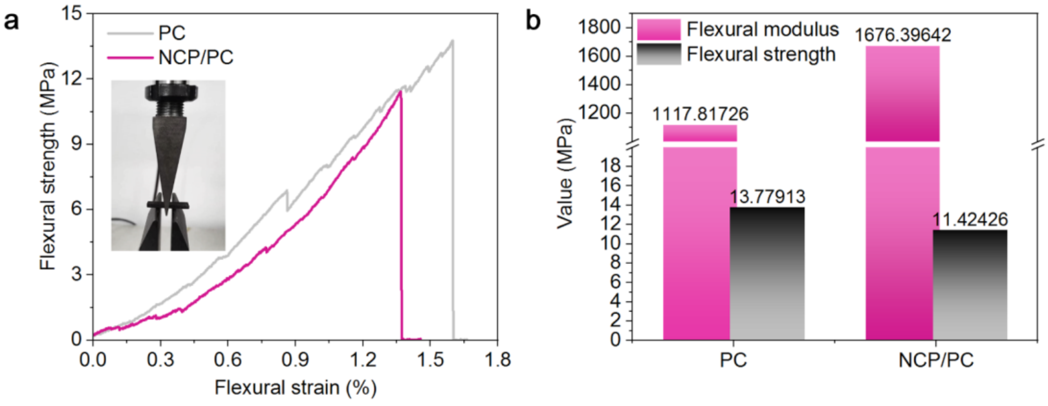

Supplementary Fig. 10 | Mechanical three-point bending properties of NCP/PC and PC. (a) Flexural stress–flexural strain curves for NCP/PC and PC. Inset illustrates the three-point bending test setup. (b) Flexural strength and modulus values of NCP/PC and PC.

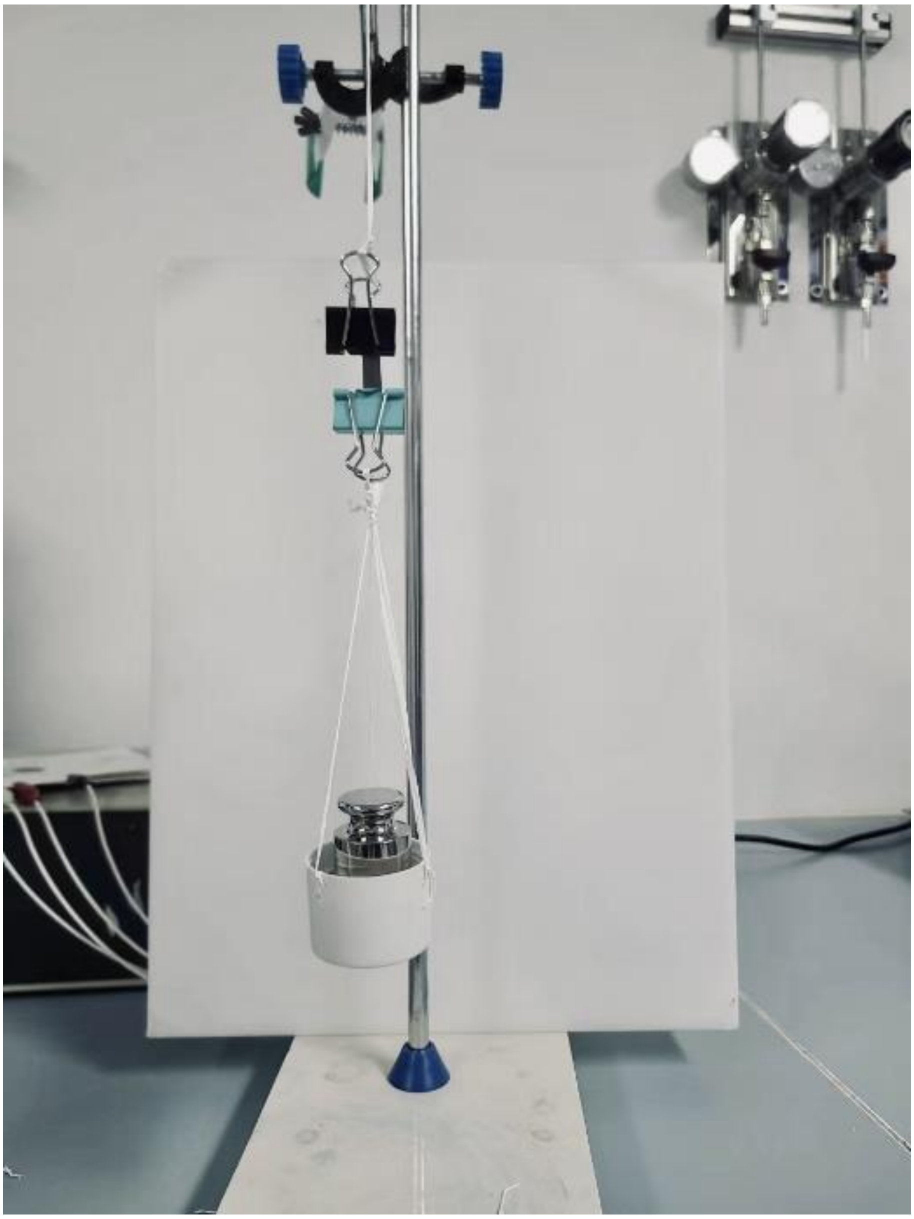

Supplementary Fig. 11 | Photo of a piece of NCP/PC easily withstanding 200 grams of weight. This NCP/PC has a size of ~20 mm × ~5 mm × ~1.9 mm.

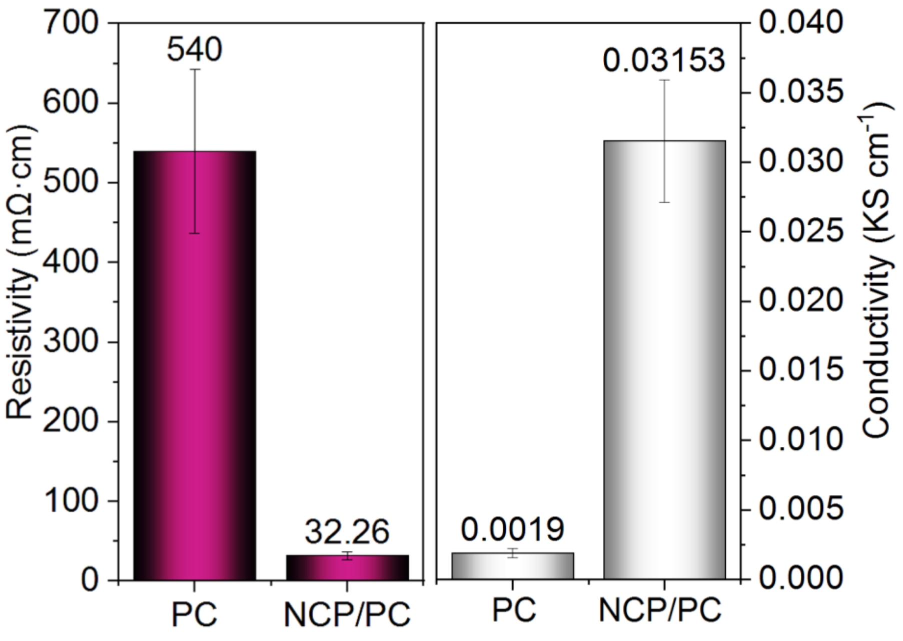

Supplementary Fig. 12 | Four-probe conductivity measurement results for both NCP/PC and PC. Error bars denote the standard deviation of experimental replicates.

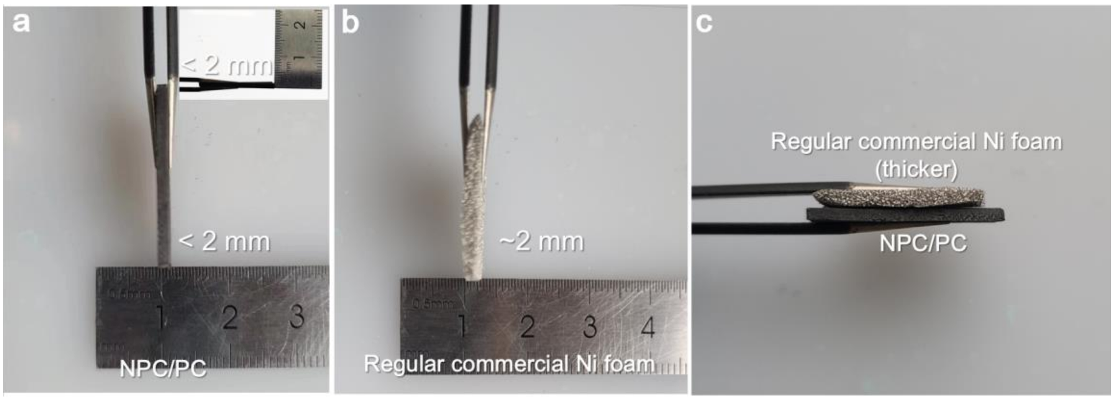

Supplementary Fig. 13 | Photos of classical Ni foam and our NCP/PC. (a) Photos of NCP/PC. Inset shows the photo taken from another angle. (b) Photos of a commercial Ni foam used for electrolysis tests. (c) Electrodes held together by a pair of tweezers to better visualize the thick difference. The photos here illustrate that the NCP/PC is not thick, and the thickness comparison of the two electrodes shows the thinner thickness of our NCP/PC.

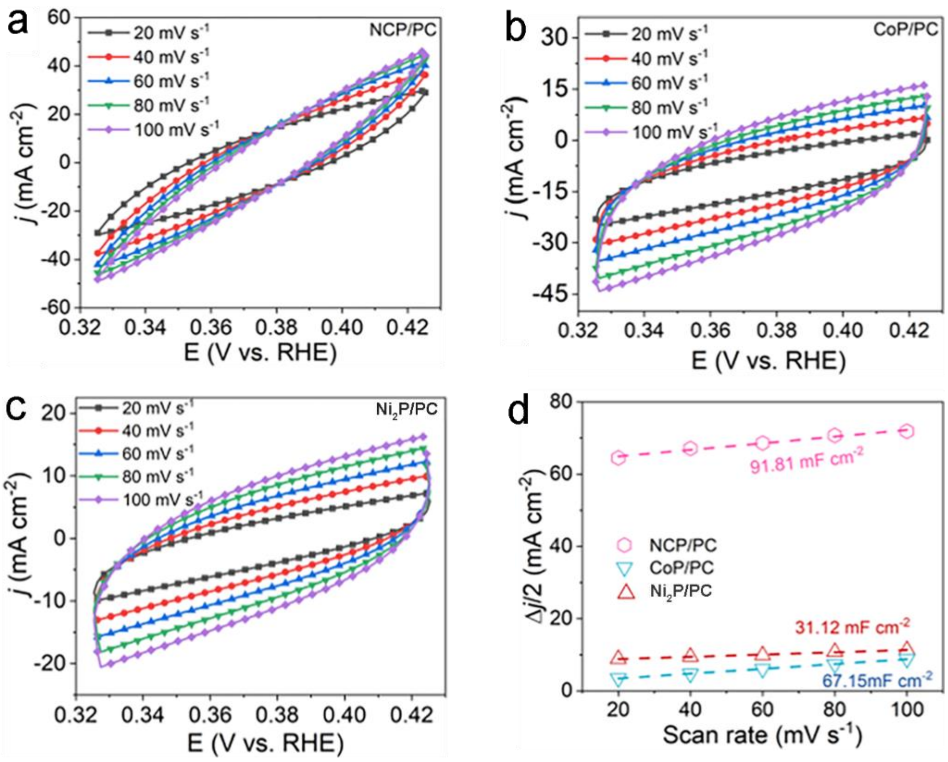

Supplementary Fig. 14 | Electrochemical C_{dl} measurements in alkaline seawater. Cyclic voltammetry curves at various scan rates ranging from 20 to 100 mV s^{-1} within the non-Faradaic electrode potential range for (a) NCP/PC, (b) CoP/PC, and (c) Ni_2P/PC. (d) Capacitive currents on the basis of scan rates for NCP/PC, CoP/PC, and Ni_2P/PC.

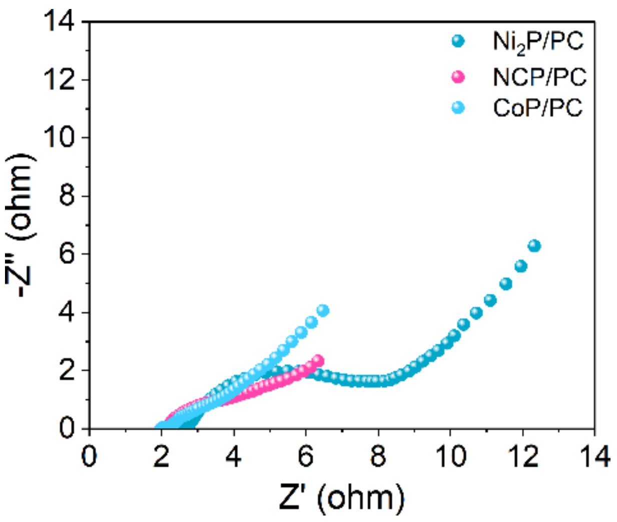

Supplementary Fig. 15 | Nyquist plots for NCP/PC, Ni_2P/PC, and CoP/PC. EIS data were collected in the frequency range of 0.1 to 500000 Hz. A much lower charge transfer resistance of NCP/PC (only 2.5 Ω) compared to its monometallic counterpart also accounts for its superior seawater reduction activity.

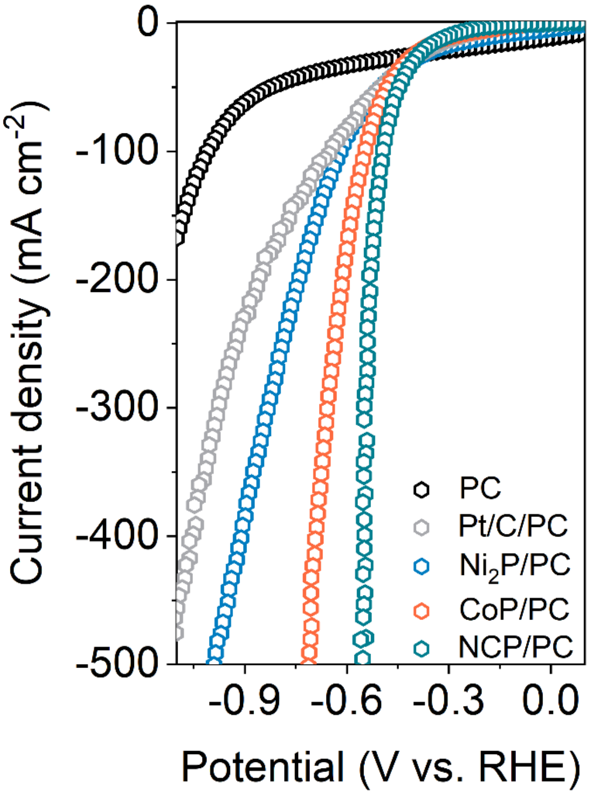

Supplementary Fig. 16 | Polarization curves demonstrating H_2 evolution activities of different electrodes in natural seawater. NCP/PC still maintains the activity superiority in natural seawater, suggesting the high water dissociation ability.

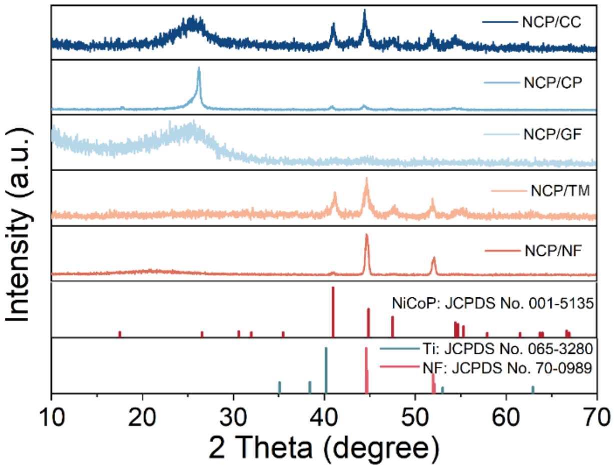

Supplementary Fig. 17 | XRD patterns of the NCP-based cathode counterparts including NCP/CC, NCP/CP, NCP/GF, NCP/TM, and NCP/NF.

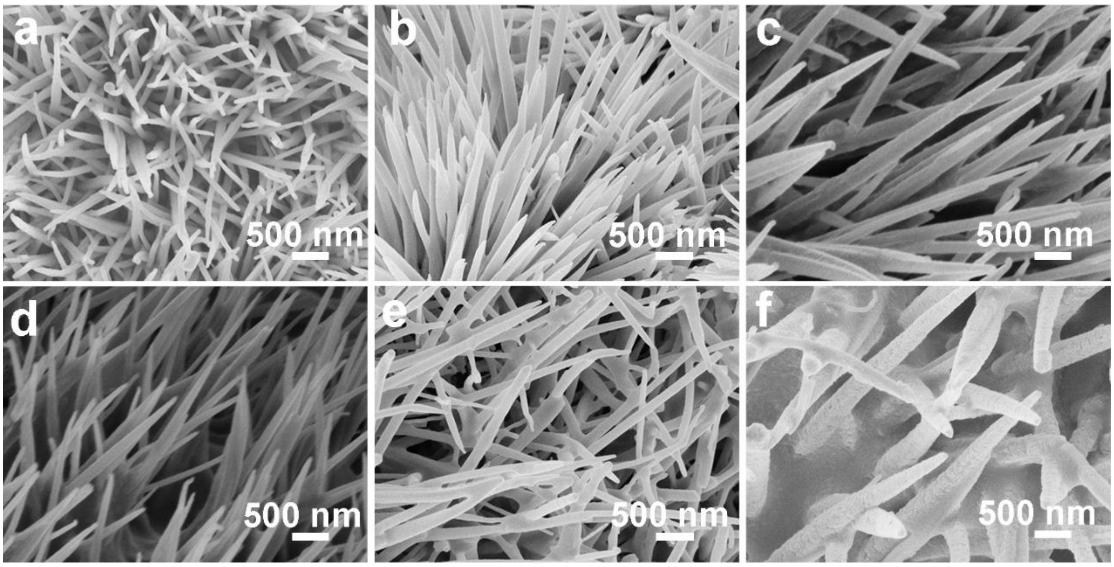

Supplementary Fig. 18 | SEM images of various NCP-based electrodes, including (a) NCP/PC, (b) NCP/NF, (c) NCP/TM, (d) NCP/CC, (e) NCP/GF, and (f) NCP/CP.

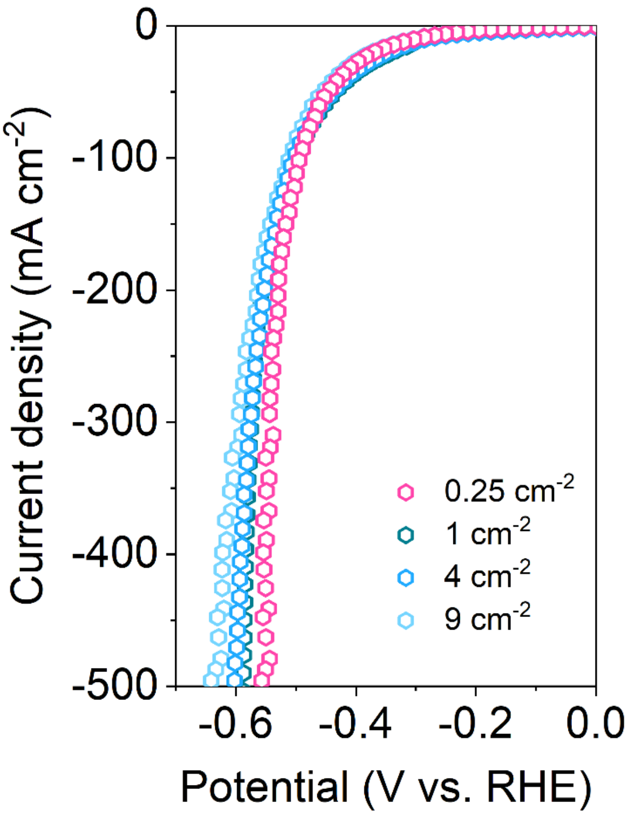

Supplementary Fig. 19 | Polarization curves recorded with NCP/PC electrodes in different sizes (geometric area: 0.25 cm^2, 1 cm^2, 4 cm^2, and 9 cm^2). The measured electrochemistry data here were obtained in a three-electrode system with natural seawater as the electrolyte. Be aware that most reports in the field of seawater electrolysis do not use electrodes larger than 1 cm^2. Our prepared electrodes still exhibit better activities even at the size of 4cm^2 as well as 9cm^2.

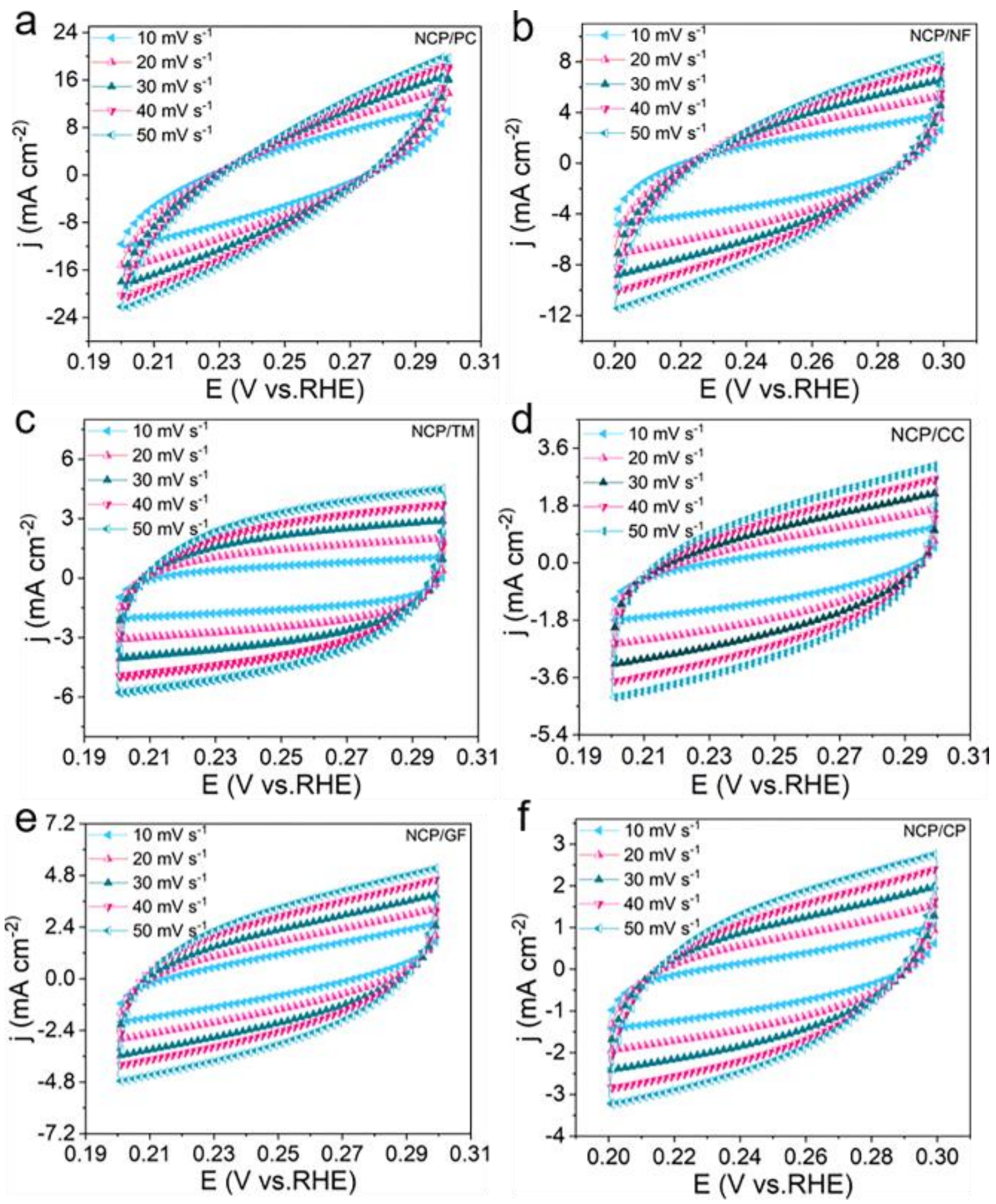

Supplementary Fig. 20 | Electrochemical C_{dl} measurements in natural seawater. Cyclic voltammetry curves at various scan rates ranging from 10 to 50 mV s^{-1} within the non-Faradaic potential range for (a) NCP/PC, (b) NCP/NF, (c) NCP/TM, (d) NCP/CC, (e) NCP/GF, and (f) NCP/CP.

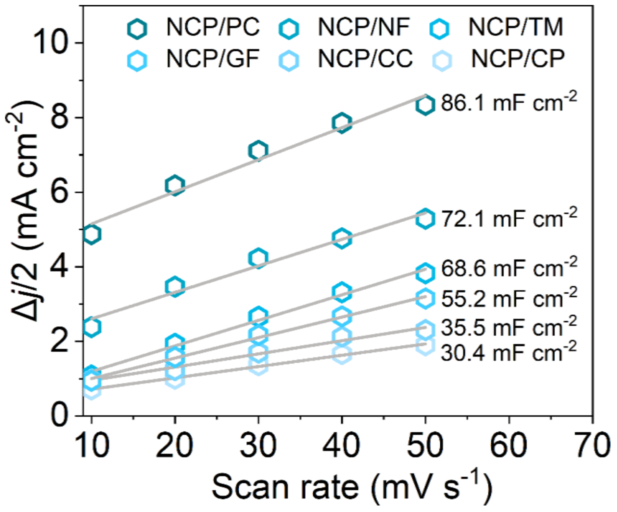

Supplementary Fig. 21 | Capacitive current densities of voltammetry curves as the function of scan rates correspondingly. An obvious increase in the C_{dl} (86.1 mF cm^{-2}) for the NCP/PC sample confirms the proliferation of catalytically active sites.

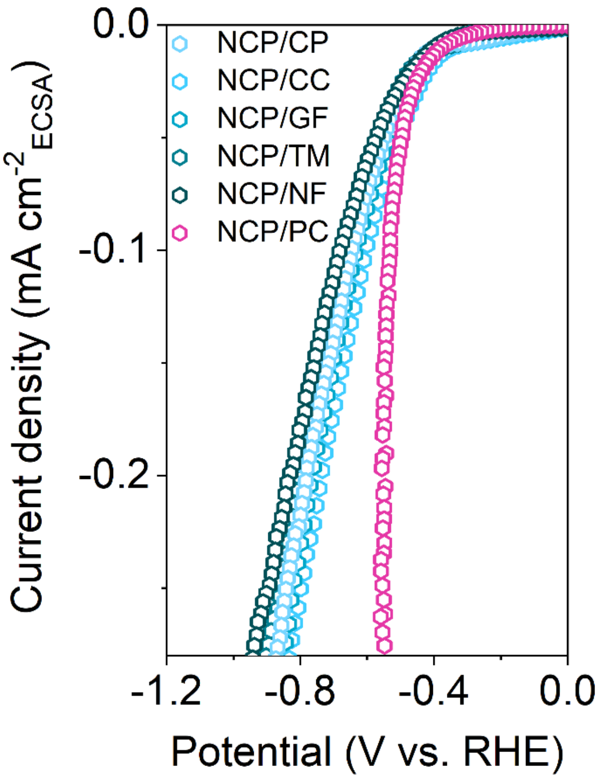

Supplementary Fig. 22 | ECSA-normalized linear voltammetry sweep curves for NCP/PC, NCP/NF, NCP/TM, NCP/GF, NCP/CC, and NCP/CP in natural seawater. NCP/CP still exhibits the largest ECSA-normalized current density, indicating the best electrocatalytic activity is ascribed to an enlarged ECSA and the higher intrinsic activity (please refer to: *Nat. Commun.* **12**, 4587 (2021)).

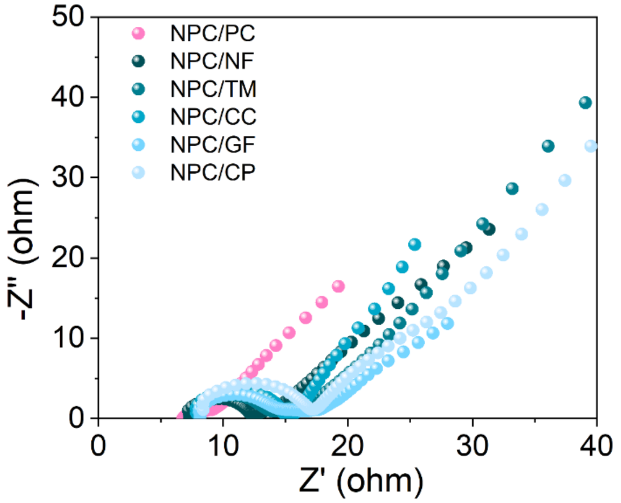

Supplementary Fig. 23 | Nyquist plots in natural seawater for NCP/PC, NCP/NF, NCP/TM, MCP/CC, NCP/GF, and NCP/CP. The plots show the minimum magnitude of the semi-circle of NCP/PC (an electrochemical resistance of 9 Ω) among all the tested samples.

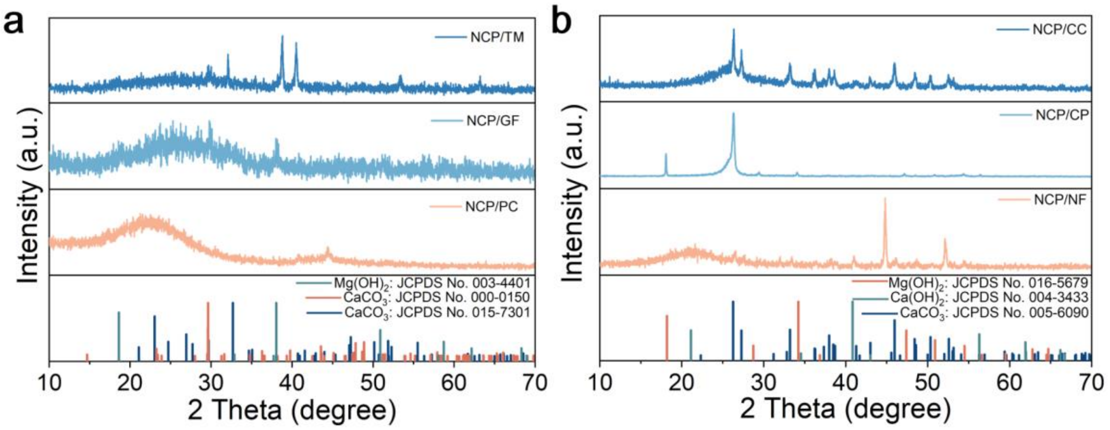

Supplementary Fig. 24 | XRD patterns of all the NCP-based cathodes after 10 h of eNSR catalysis. (a) XRD patterns of NCP/TM, NCP/GF, and NCP/PC after 10-h eNSR. (b) XRD patterns of NCP/CC, NCP/CP, and NCP/NF after 10-h eNSR. The absence of characteristic peaks of Mg^{2+}/Ca^{2+} precipitates in the XRD pattern for the NCP/PC indicates that only the NCP/PC possesses the strong anti-precipitation ability. The NCP/PC thus has exclusively high resistance to precipitation, in contrast to other NCP-based cathodes that were covered by a mass of Mg^{2+}/Ca^{2+} precipitates.

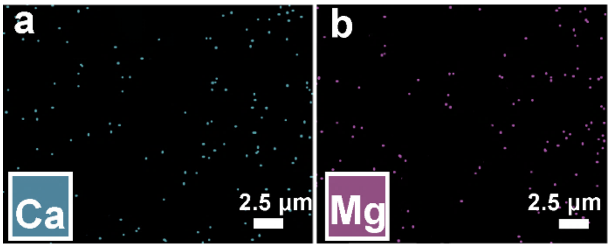

Supplementary Fig. 25 | Elemental distribution of (a) Ca and (b) Mg for NCP/PC after the long-term eNSR electrolysis. The very trace Ca and Mg signals come from the seawater residue on the electrode surface.

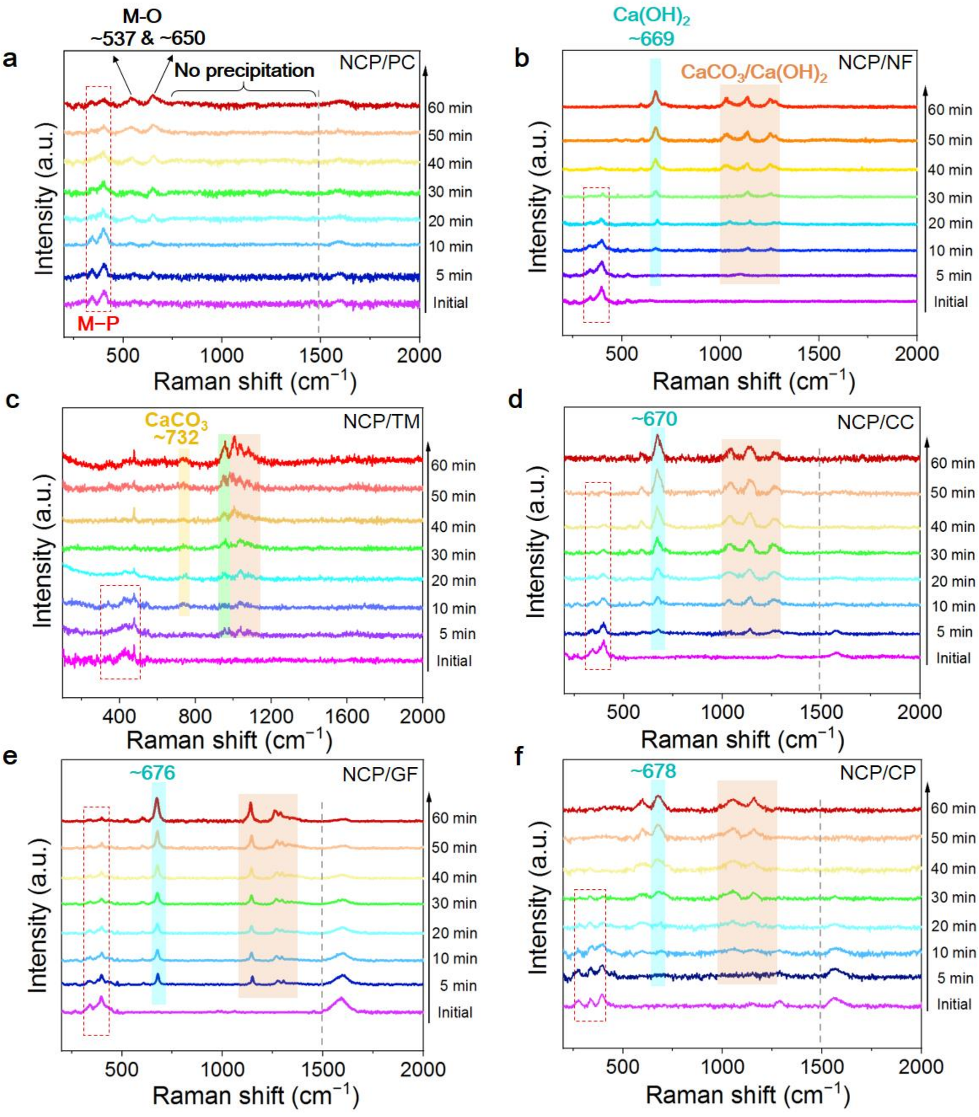

Supplementary Fig. 26 | In situ Raman spectra for various NCP-based cathodes in natural seawater (wavenumber range: from 200 to 2000 cm^{-1}). Time-dependent Raman data for (a) NCP/PC, (b) NCP/NF, (c) NCP/TM, (d) NCP/CC, (e) NCP/GF, and (f) NCP/CP were recorded under a fixed electrode potential.

A peak at around 669 cm^{-1} can be observed for NCP/NF, corresponding to Eg (R) mode of $Ca(OH)_2$ (please refer to: *Solid State Commun.* 8, 541–543 (1970)). Moreover, the Raman bands in the range of ~1000 to ~1300 cm^{-1} indicate that the surface precipitates should contain $CaCO_3$ and $Ca(OH)_2$ (please refer to: *RSC Adv.* **6**, 104537–104548 (2016), *Phys. Chem. Minerals* **46**, 229–244 (2019), *Acta Geotech.* **16**, 3229–3237 (2021). *Chem. Commun.* **53**, 6657–6660 (2017), *Appl. Environ. Microbiol.* **81**,

7403–7410 (2015), *Heritage* **4**, 3970–3986 (2021), *J. Appl. Electrochem.* **39**, 39–44 (2009), *J. Power Sources***172**, 435–445 (2007)).

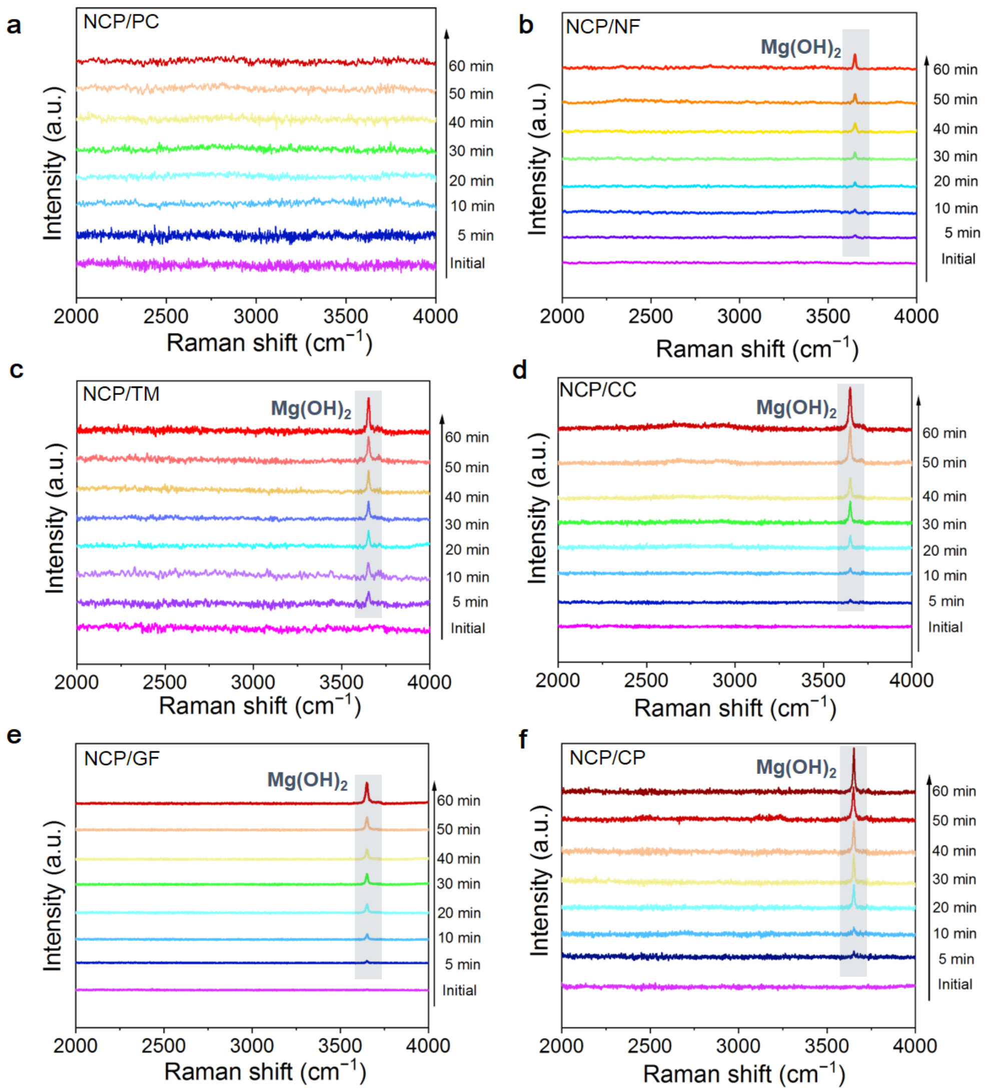

Supplementary Fig. 27 | In situ Raman spectra for various NCP-based electrodes in natural seawater (wavenumber range: from 2000 to 4000 cm^{-1}). Time-dependent Raman data for (a) NCP/PC, (b) NCP/NF, (c) NCP/TM, (d) NCP/CC, (e) NCP/GF, and (f) NCP/CP were recorded under a fixed electrode potential.

Raman spectra in higher wavenumbers ranging from 2000 cm^{-1} to 4000 cm^{-1} reveal that NCP/NF, NCP/TM, NCP/CC, NCP/GF, and NCP/CP are all covered by insoluble $Mg(OH)_2$ precipitates (please refer to: *Sci. Rep.* **6**, 20525 (2016), *J. Geophys. Res. Solid Earth* 124, 8267–8280 (2019)). In sharp contrast, NCP/PC does not show any Raman peak in this wavenumber range, fully indicating its superb anti-precipitation ability.

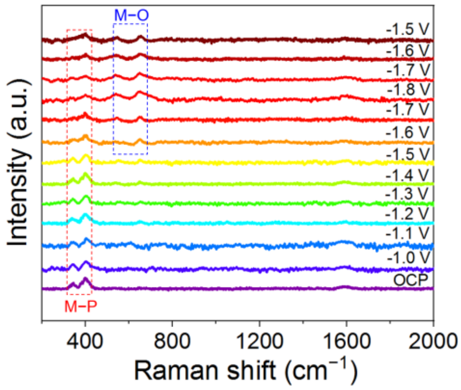

Supplementary Fig. 28 | In situ Raman spectra for NCP/PC under different electrode potentials in natural seawater.

According to previous Raman studies on metal phosphides and NiCo-based materials during/after various electrochemical processes, such as HER catalysis, (please refer to: *Energy Environ. Sci.* **15**, 727–739 (2022), *ACS Catal.* **10**, 81–92 (2020), *Chem. Eng. J.* **473**, 145397 (2023), *Adv. Mater.* **34**, 2107548 (2022), *Int. J. Energy Res.* **46**, 13035-13043 (2022), *Nano Energy* **114**, 108601 (2023), *Nat. Commun.* **14**, 1949 (2023), *J. Mater. Chem. A*, **9**, 18421–18430 (2021), *Appl. Catal. B* **316**, 121678 (2022), *Nat. Catal.* **4**, 1050–1058 (2021), *Adv. Energy Mater.* **13**, 2204114, (2023), *Chem. Eng. J.* **435**, 134261, (2022)), the two pronounced peaks appearing at the potentials more negative than −1.5 V in the Raman spectra of NCP/PC should belong to M–O bonds (M = Ni or Co) in metal oxyhydroxides.

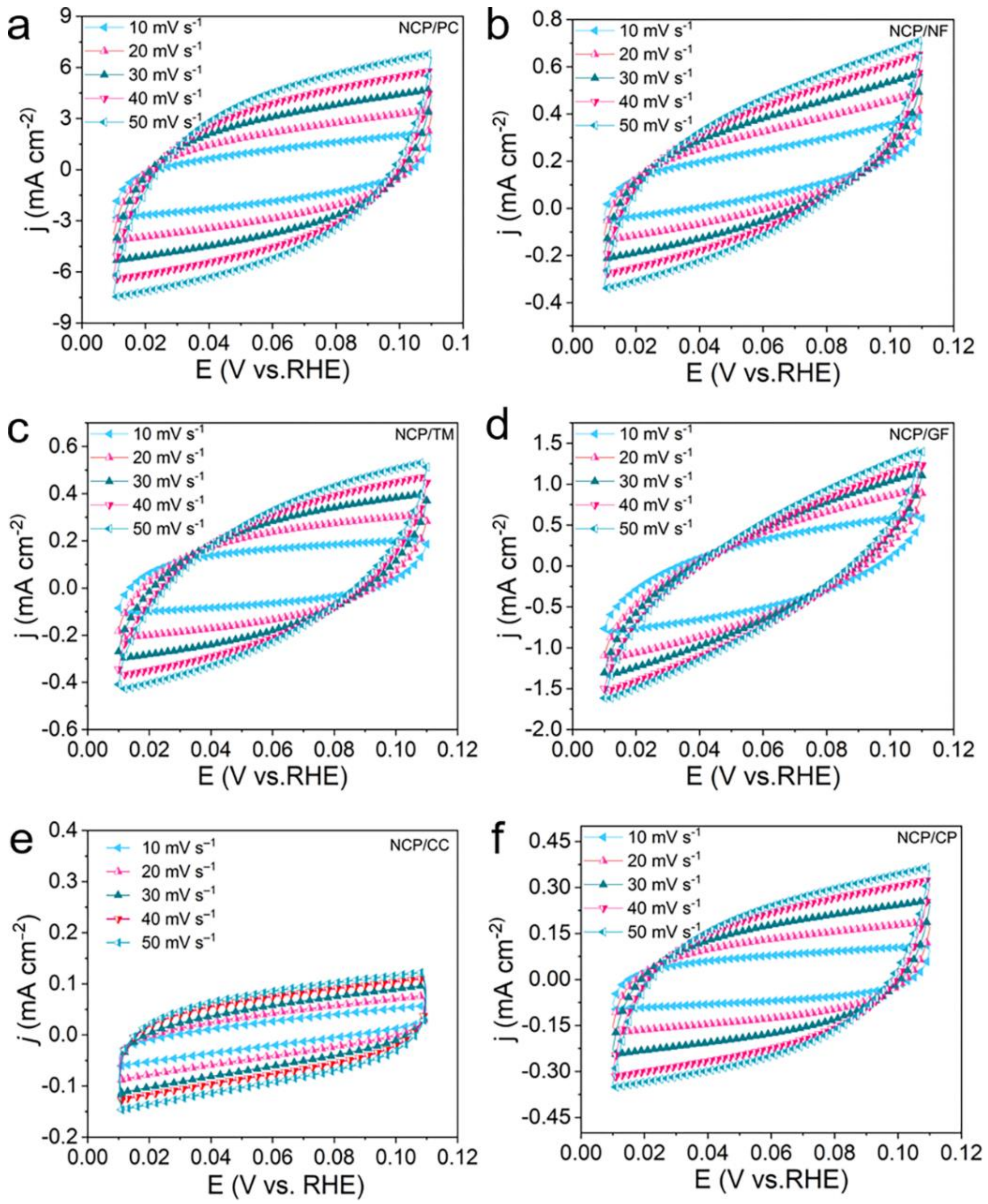

Supplementary Fig. 29 | Electrochemical C_{dl} measurements for different anodes after 10 h of NSR electrolysis. Cyclic voltammetry curves at various scan rates ranging from 10 to 50 mV s^{-1} within the non-Faradaic potential range for (a) NCP/PC, (b) NCP/NF, (c) NCP/TM, (d) NCP/GF, (e) NCP/CC, and (f) NCP/CP.

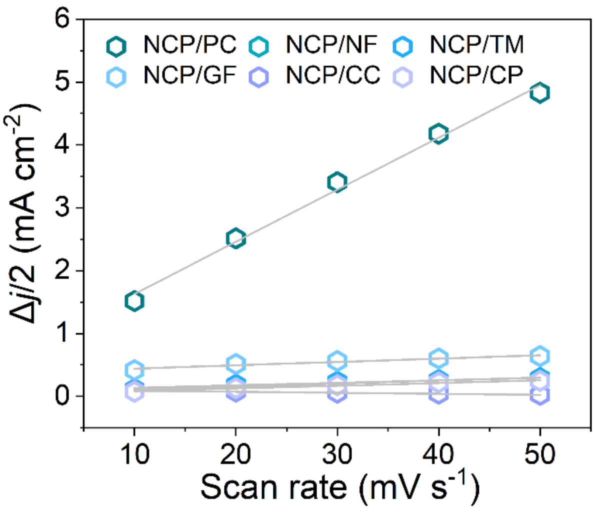

Supplementary Fig. 30 | Capacitive current densities of voltammetry curves as the function of scan rates correspondingly. The C_{dl} for the reconstructed NCP/PC is 83.1 mF cm^{-2}, close to that of pristine NCP/PC (86.1 mF cm^{-2}).

Electrochemical C_{dl} values for NCP with different substrates (CP, CC, GF, TM, and NF) after the 10 h of NSR electrolysis are 83.1 mF cm^{-2} for the NCP/PC, 4.8 mF cm^{-2} for the NCP/NF, 4.3 mF cm^{-2} for the NCP/CP, 1.7 mF cm^{-2} for the NCP/CC, 5.4 mF cm^{-2} for the NCP/GF, and 4.0 mF cm^{-2} for the NCP/TM. Therefore, only the NCP/PC demonstrates a minimal change in ECSA. The remaining electrodes have drastically decreased ECSAs, which should be resulted from the heavy precipitation covering the electrode surface.

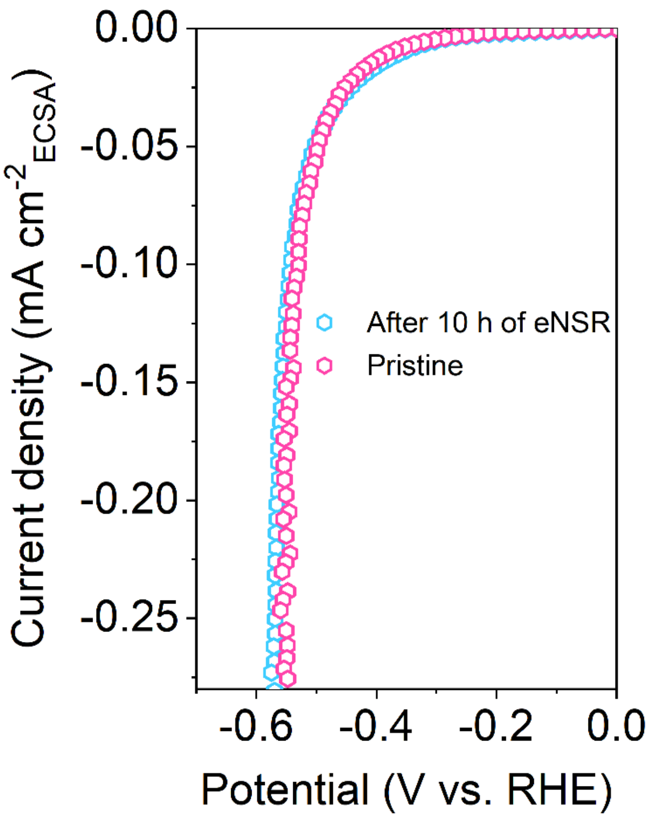

Supplementary Fig. 31 | Comparison of ECSA-normalized *j* on NCP/PC before and after 10 h of eNSR. ECSA-normalized E-*j* curve after the long-term eNSR (the pink curve) does not show a marked attenuation compared with the ECSA-normalized E-*j* curve before the electrolysis (the blue curve), suggesting that the NCP/PC possesses the comparable intrinsic activity as before long-term electrolysis.

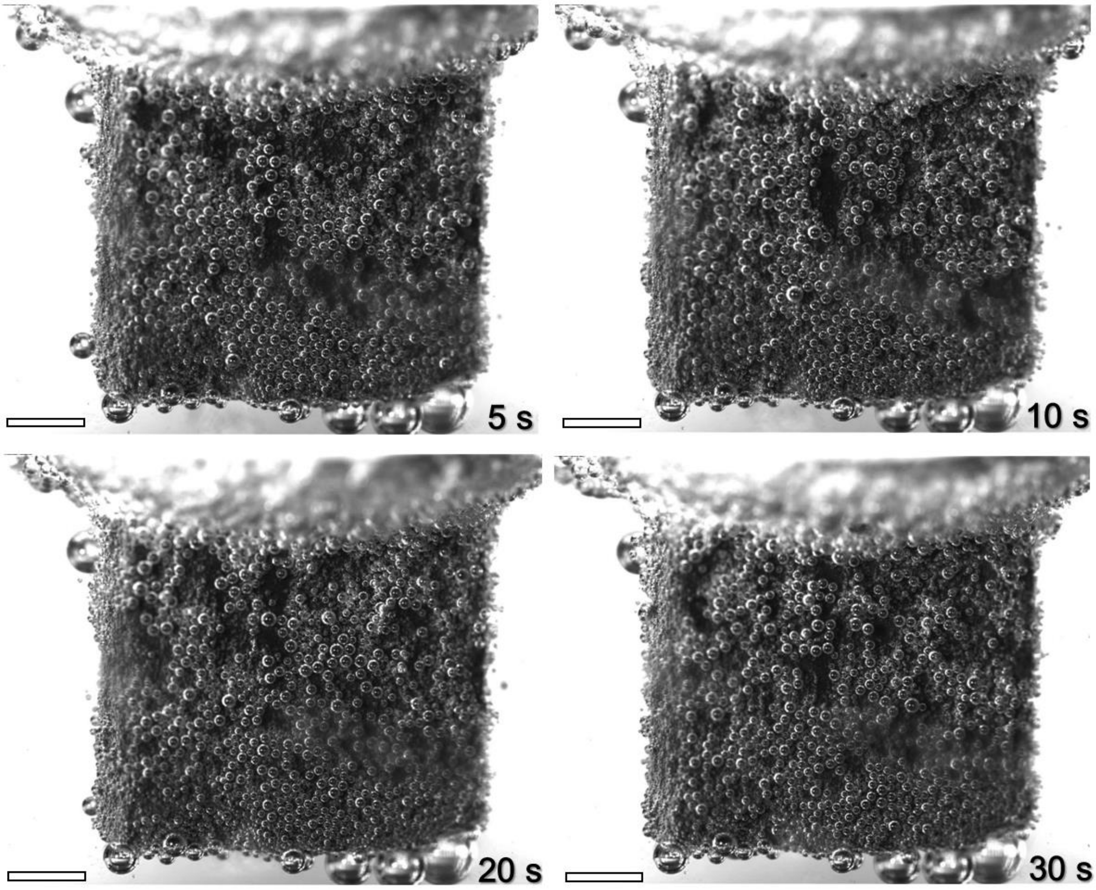

Supplementary Fig. 32 | Movements of H_2 bubbles and precipitates during the eNSR process on the front of the NCP/PC cathode, captured in real time by a high-speed camera. Scale bars: 1 mm.

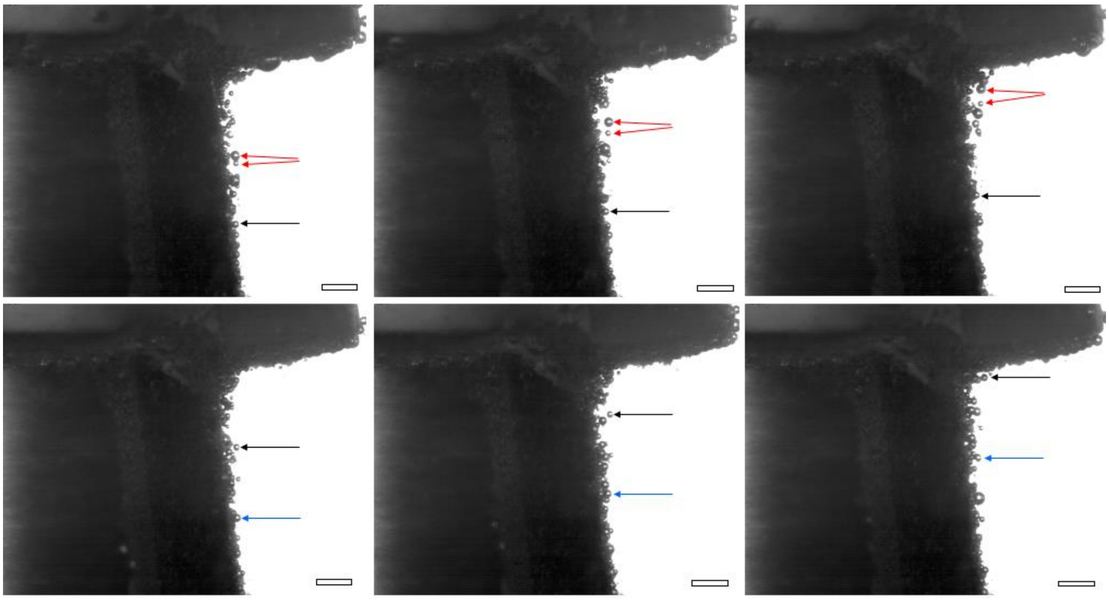

Supplementary Fig. 33 | Movements of H_2 bubbles and precipitates during the eNSR process on the NCP/PC cathode (from a specific angle), captured in real time by a high-speed camera. Some bubbles are labeled in the figure for purpose of guiding the eyes. Scale bars: 1 mm.

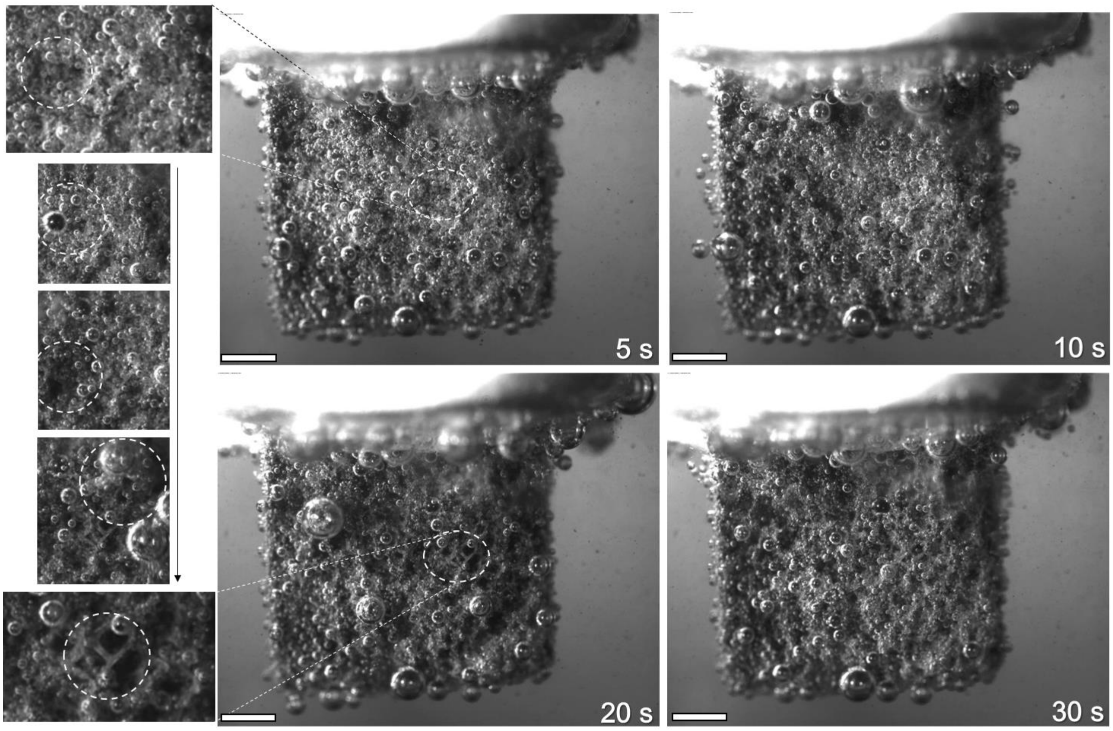

Supplementary Fig. 34 | Movements of H_2 bubbles and precipitates during the eNSR process on the front of the NCP/NF cathode, captured in real time by a high-speed camera. Scale bars: 1 mm.

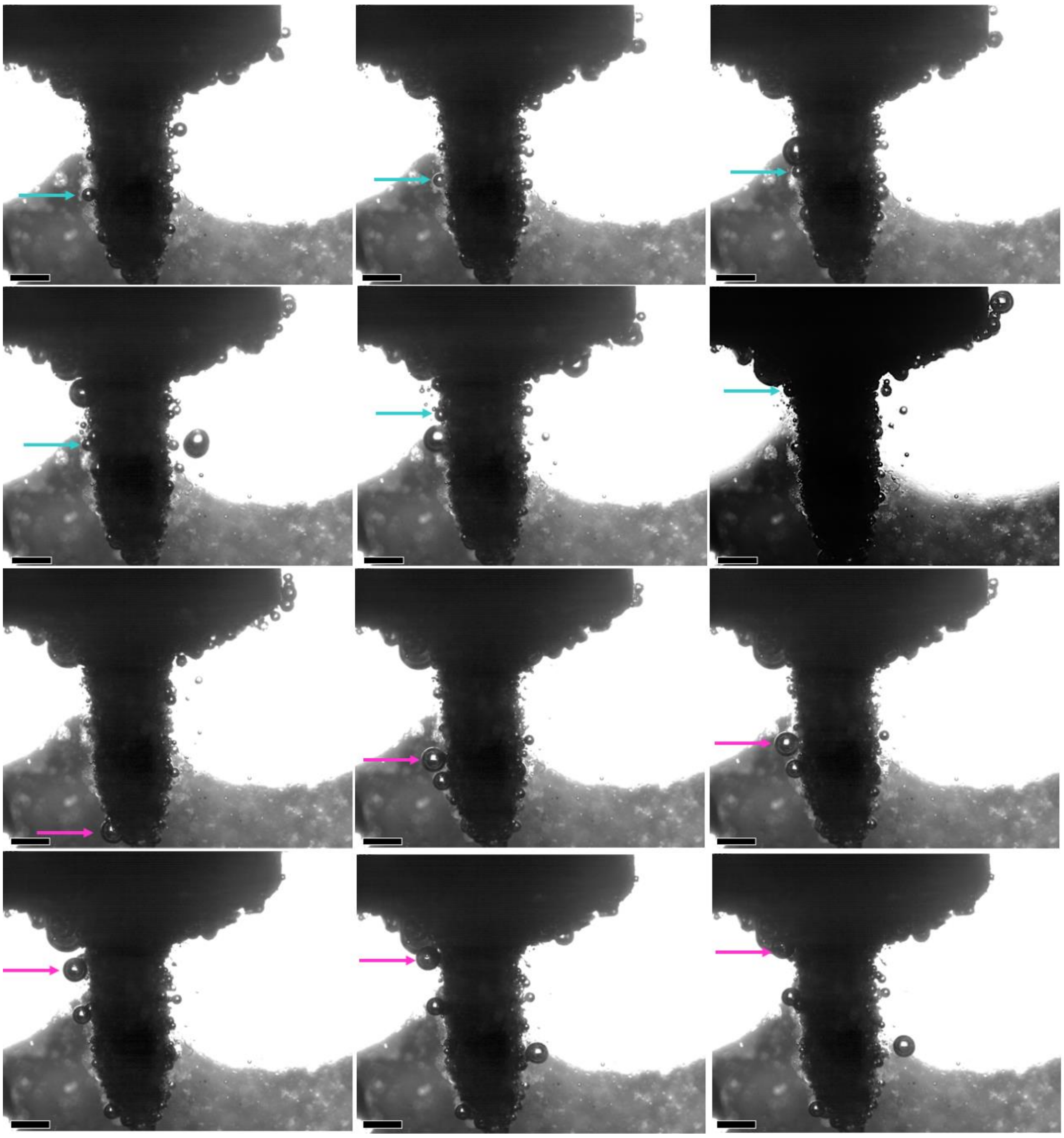

Supplementary Fig. 35 | Movements of H_2 bubbles and precipitates during the eNSR process on the side of the NCP/NF cathode, captured in real time by a high-speed camera. Some bubbles are labeled in the figure for purpose of guiding the eyes. Scale bars: 1 mm.

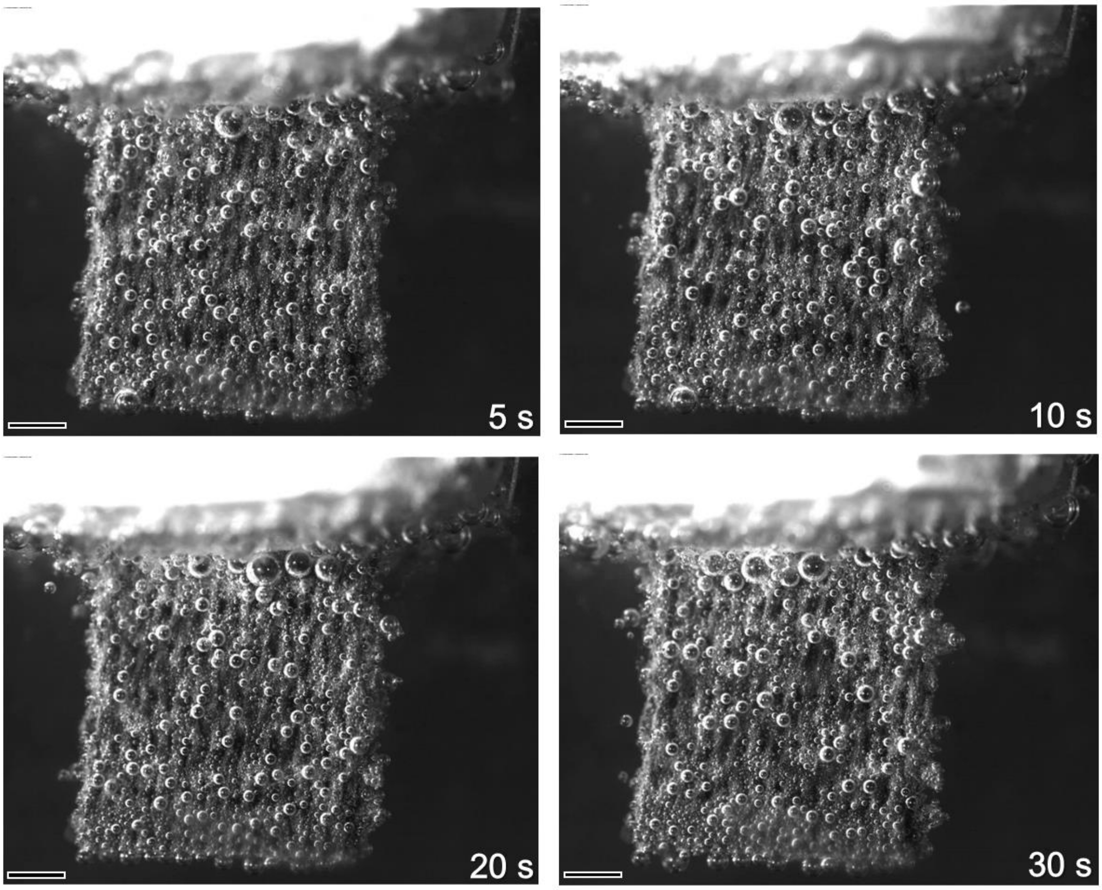

Supplementary Fig. 36 | Movements of H_2 bubbles and precipitates during the eNSR process on the front of the NCP/TM cathode, captured in real time by a high-speed camera. Scale bars: 1 mm.

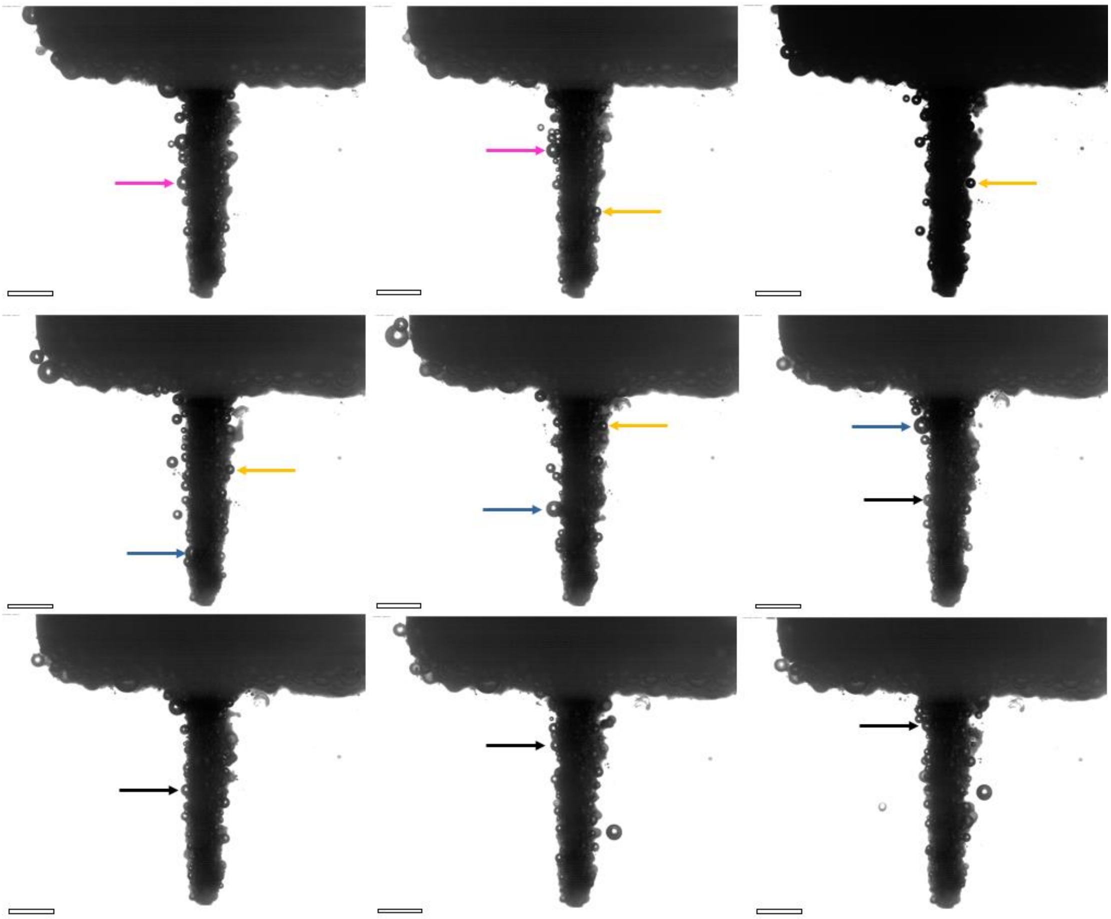

Supplementary Fig. 37 | Movements of H_2 bubbles and precipitates during the eNSR process on the side of the NCP/TM cathode, captured in real time by a high-speed camera. Some bubbles are labeled in the figure for purpose of guiding the eyes. Scale bars: 1 mm.

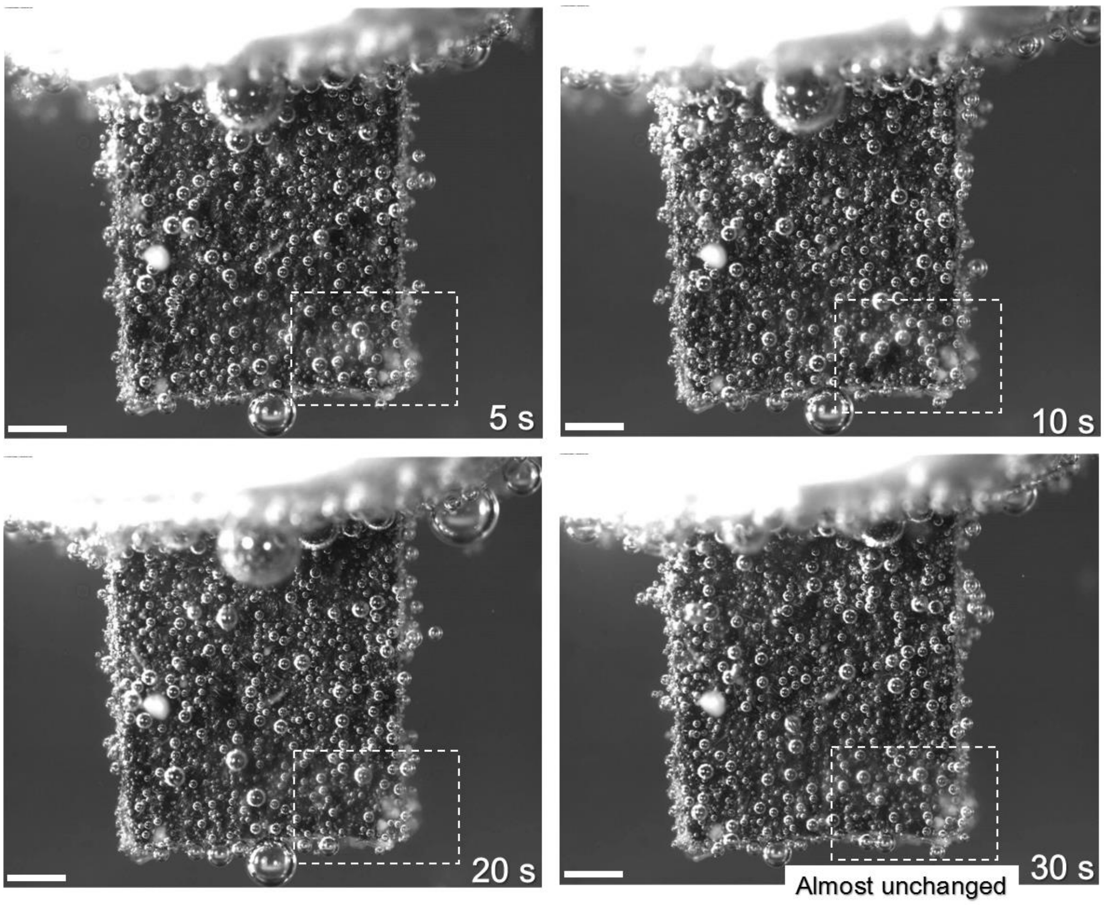

Supplementary Fig. 38 | Movements of H_2 bubbles and precipitates during the eNSR process on the front of the NCP/CC cathode, captured in real time by a high-speed camera. Scale bars: 1 mm.

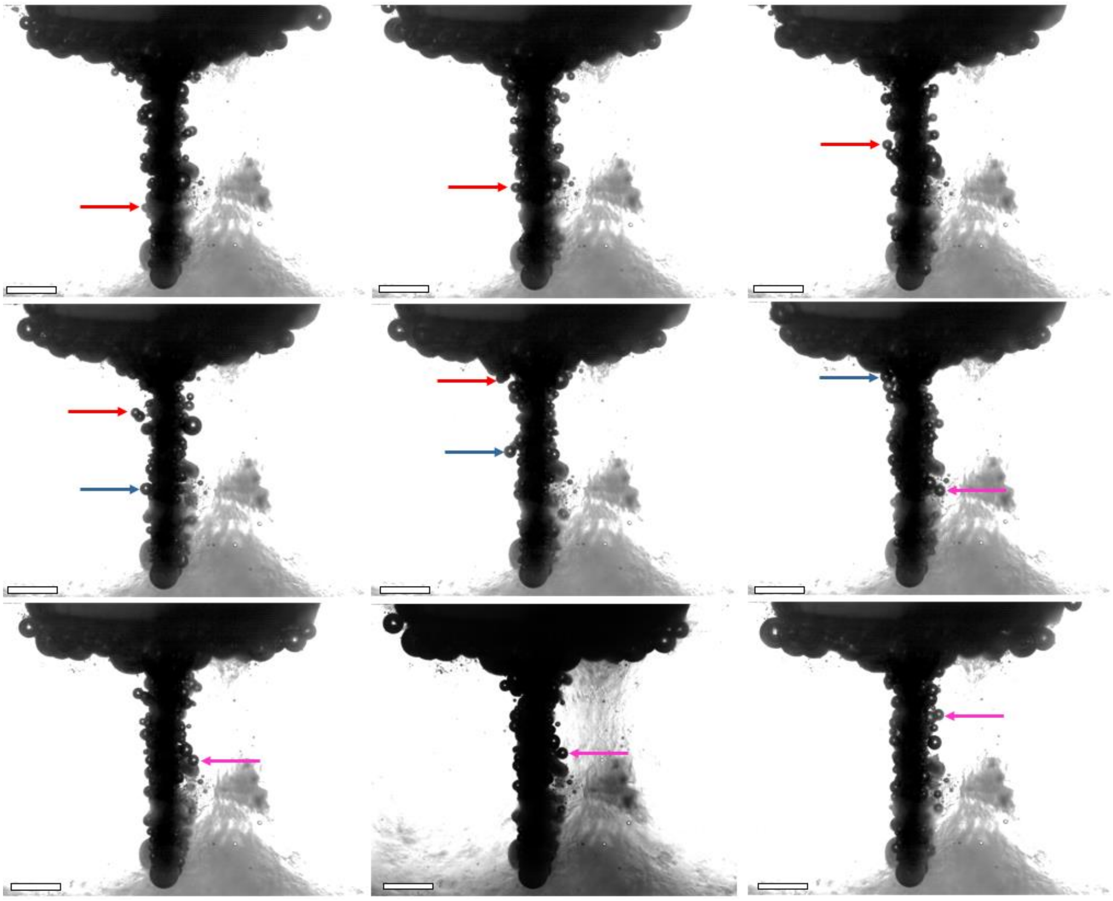

Supplementary Fig. 39 | Movements of H_2 bubbles and precipitates during the eNSR process on the side of the NCP/CC cathode, captured in real time by a high-speed camera. Some bubbles are labeled in the figure for purpose of guiding the eyes. Scale bars: 1 mm.

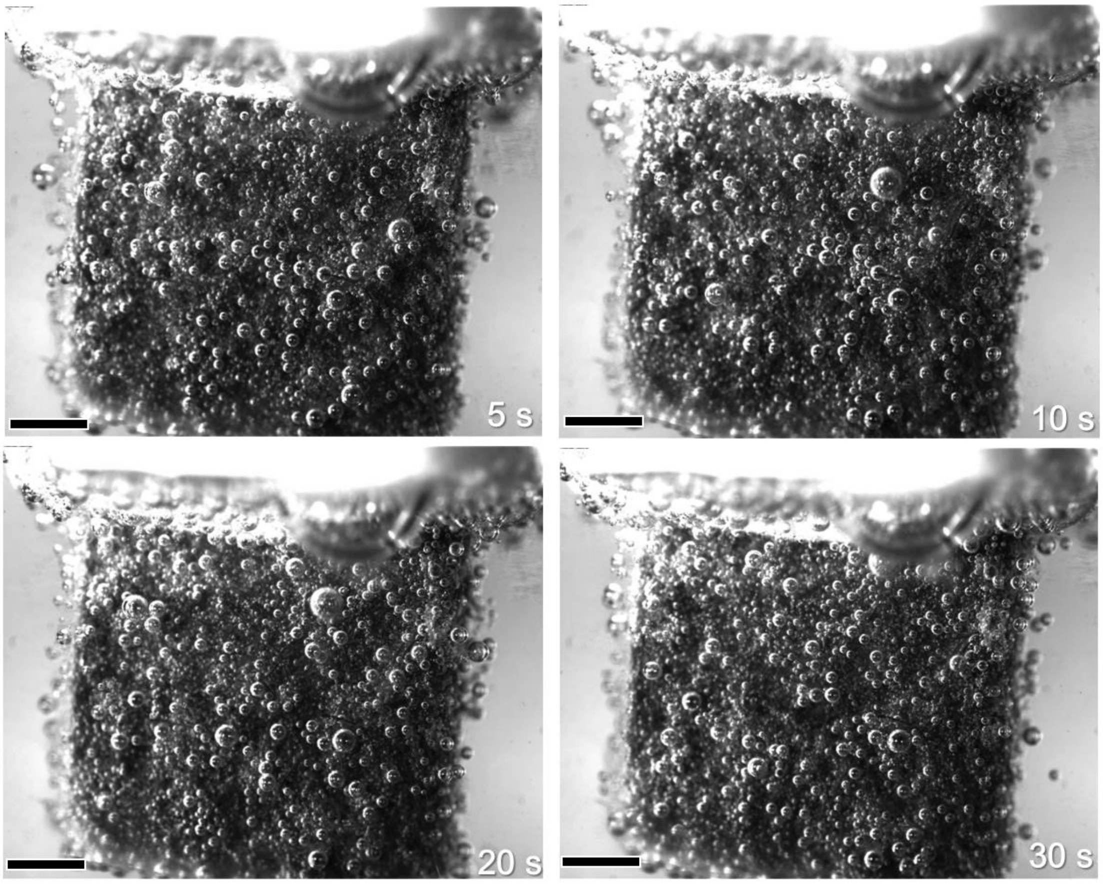

Supplementary Fig. 40 | Movements of H_2 bubbles and precipitates during the eNSR process on the front of the NCP/GF cathode, captured in real time by a high-speed camera. Scale bars: 1 mm.

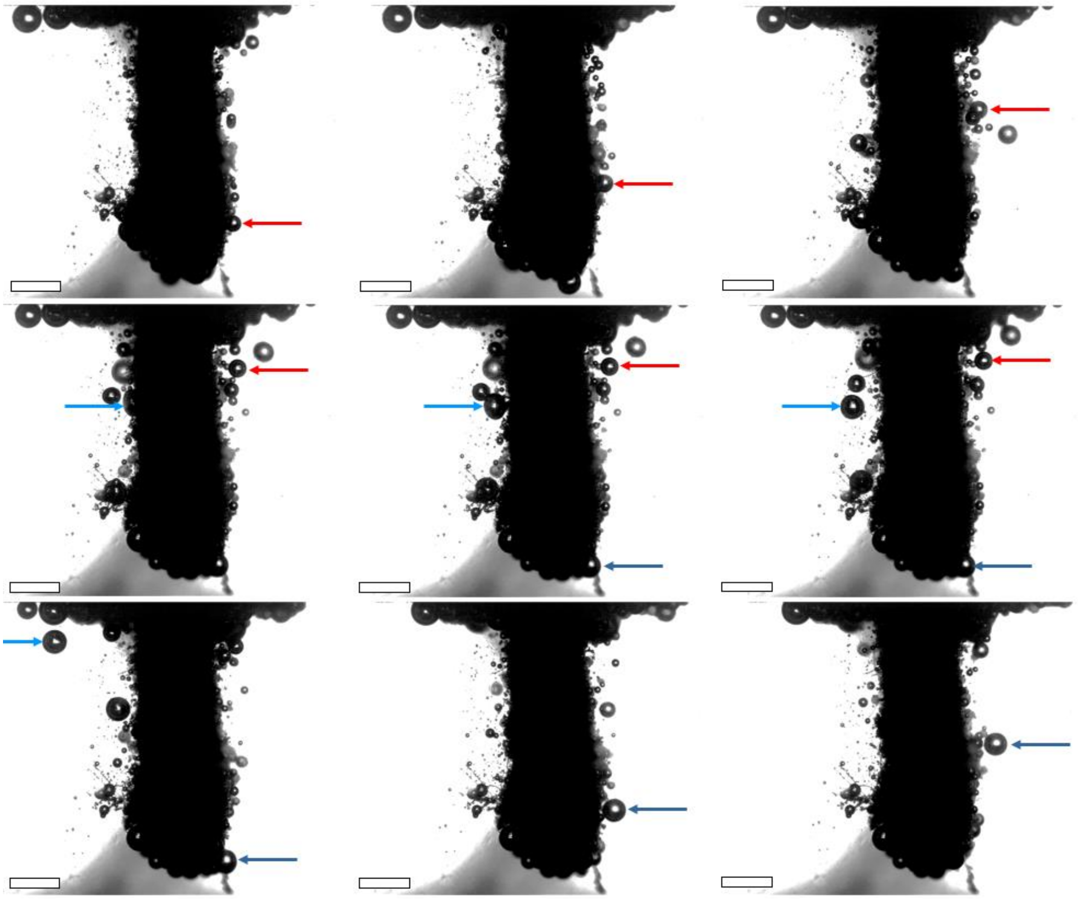

Supplementary Fig. 41 | Movements of H_2 bubbles and precipitates during the eNSR process on the side of the NCP/GF cathode, captured in real time by a high-speed camera. Some bubbles are labeled in the figure for purpose of guiding the eyes. Scale bars: 1 mm.

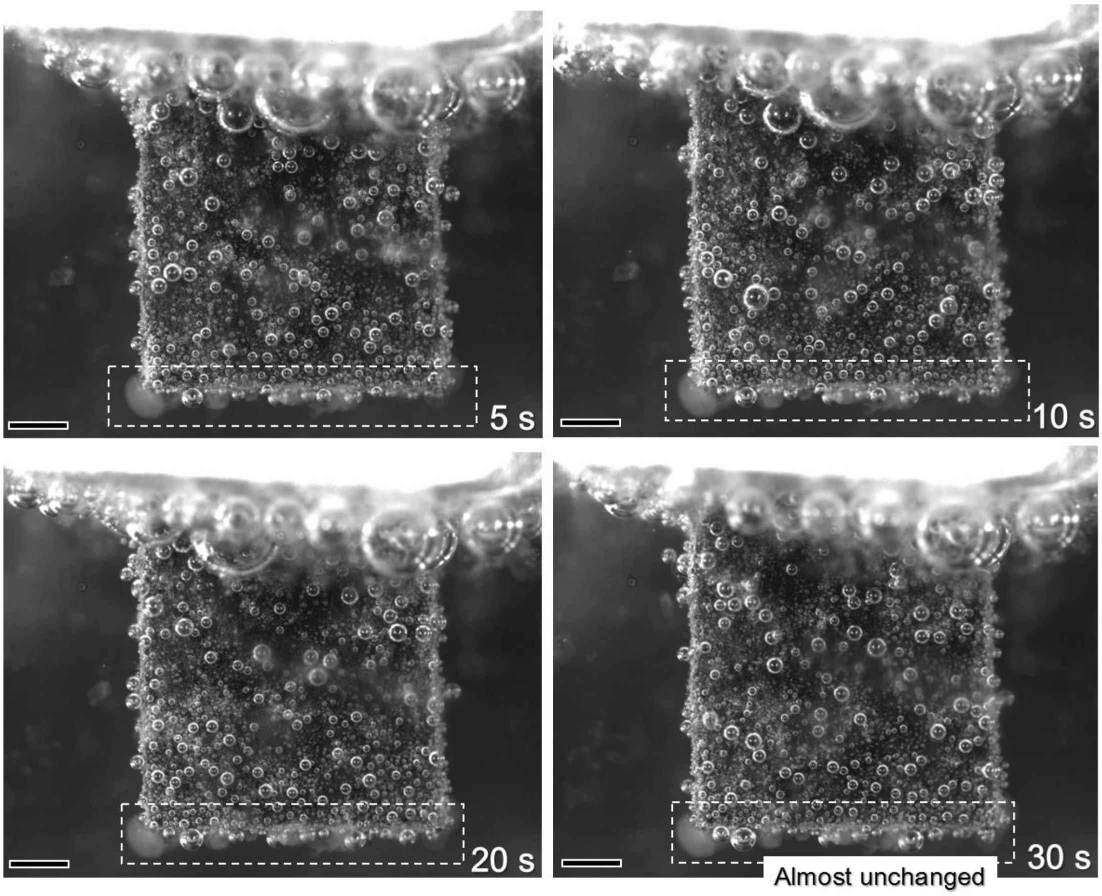

Supplementary Fig. 42 | Movements of H_2 bubbles and precipitates during the eNSR process on the front of the NCP/CP cathode, captured in real time by a high-speed camera. Scale bars: 1 mm.

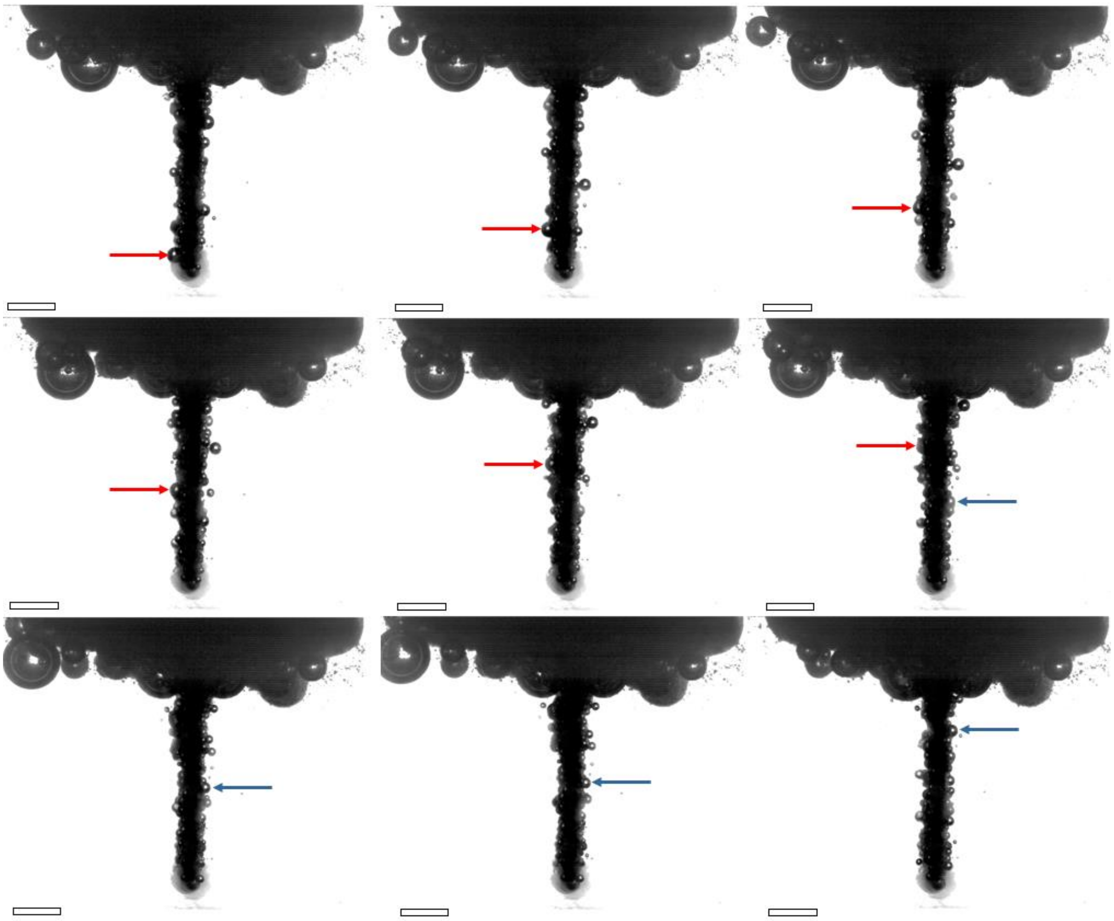

Supplementary Fig. 43 | Movements of H_2 bubbles and precipitates during the eNSR process on the side of the NCP/CP cathode, captured in real time by a high-speed camera. Some bubbles are labeled in the figure for purpose of guiding the eyes. Scale bars: 1 mm.

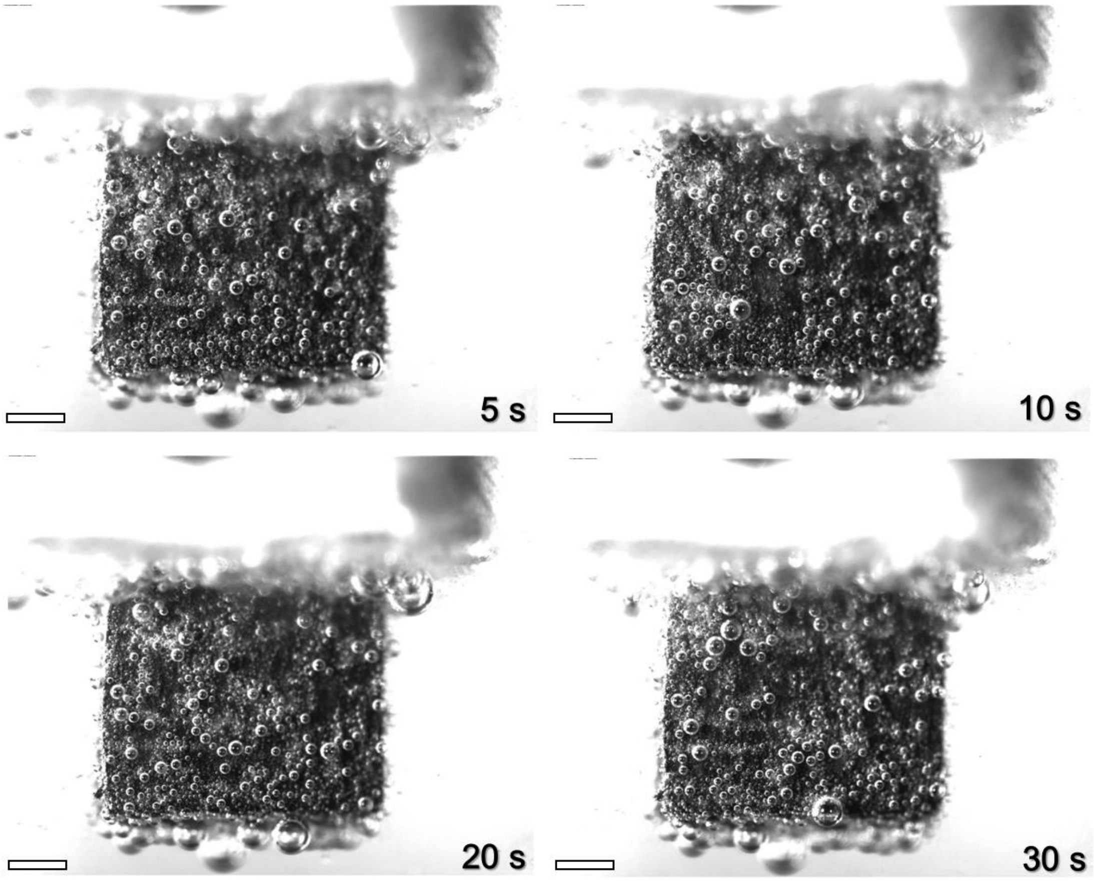

Supplementary Fig. 44 | Movements of H_2 bubbles and precipitates during the eNSR process on the front of the bare PC cathode, captured in real time by a high-speed camera. Scale bars: 1 mm.

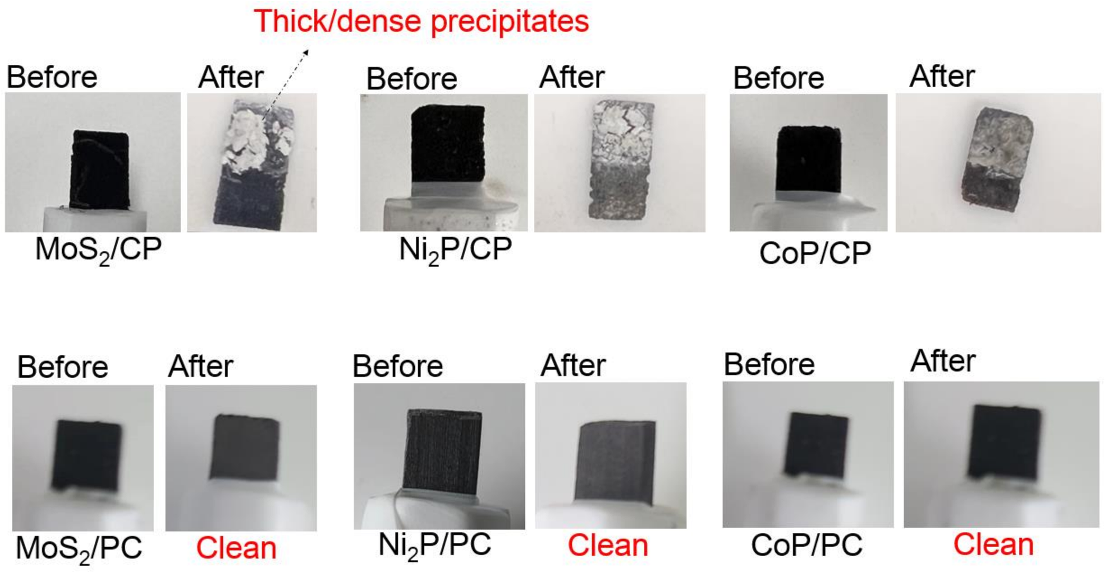

Supplementary Fig. 45 | Universally improved anti-precipitation ability achieved by the PC-based cathodes. This figure directly demonstrates digital images of various cathodes before and after the eNSR electrolysis tests under an operating current density of –500 mA cm^{-2} for 1 h.

As expected, when the same catalysts (e.g., MoS_2 nanosheets) were prepared on CP or PC, significant differences can also be found in the anti-precipitation ability of the resulting electrodes (MoS_2/CP and MoS_2/PC). For the PC-based cathodes, including MoS_2/PC, Ni_2P/PC and CoP/PC, no surface precipitate can be observed even after tests under an industrial level current density. In contrast, their counterparts based on CP are covered by a large amount of precipitates after tests under identical conditions. Thus, the experiments demonstrate the universality of the anti-precipitation design proposed by us.

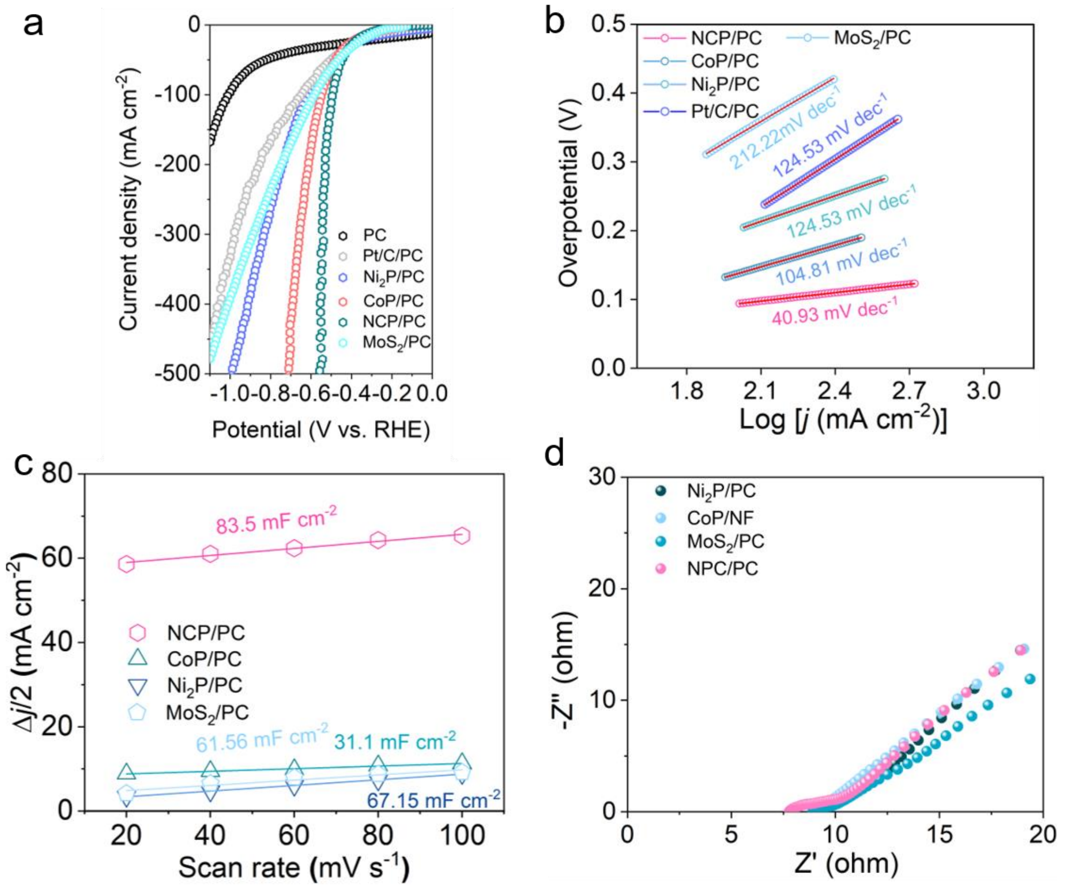

Supplementary Fig. 46 | Comparison of eNSR activities of different PC-based electrodes. (a) Linear sweep voltammetry curves, (b) Tafel slopes, (c) C_{dl} values, and (d) Nyquist plots of different PC-based electrodes, including NCP/PC, Ni_2P/PC, CoP/PC, and MoS_2/PC. The polarization curves of PC and Pt/C/PC and Tafel slope of Pt/C/PC are also given.

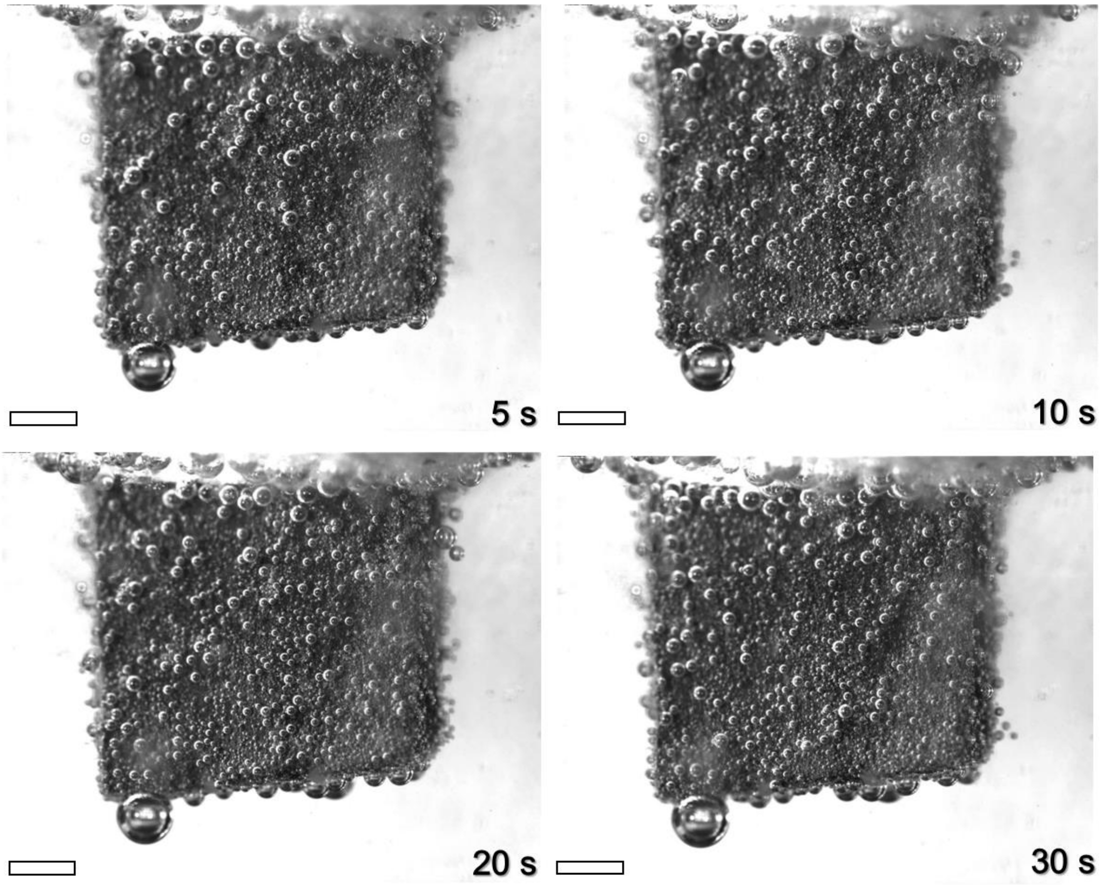

Supplementary Fig. 47 | Movements of H_2 bubbles and precipitates during the eNSR process on the front of the CoP/PC cathode, captured in real time by a high-speed camera. Scale bars: 1 mm.

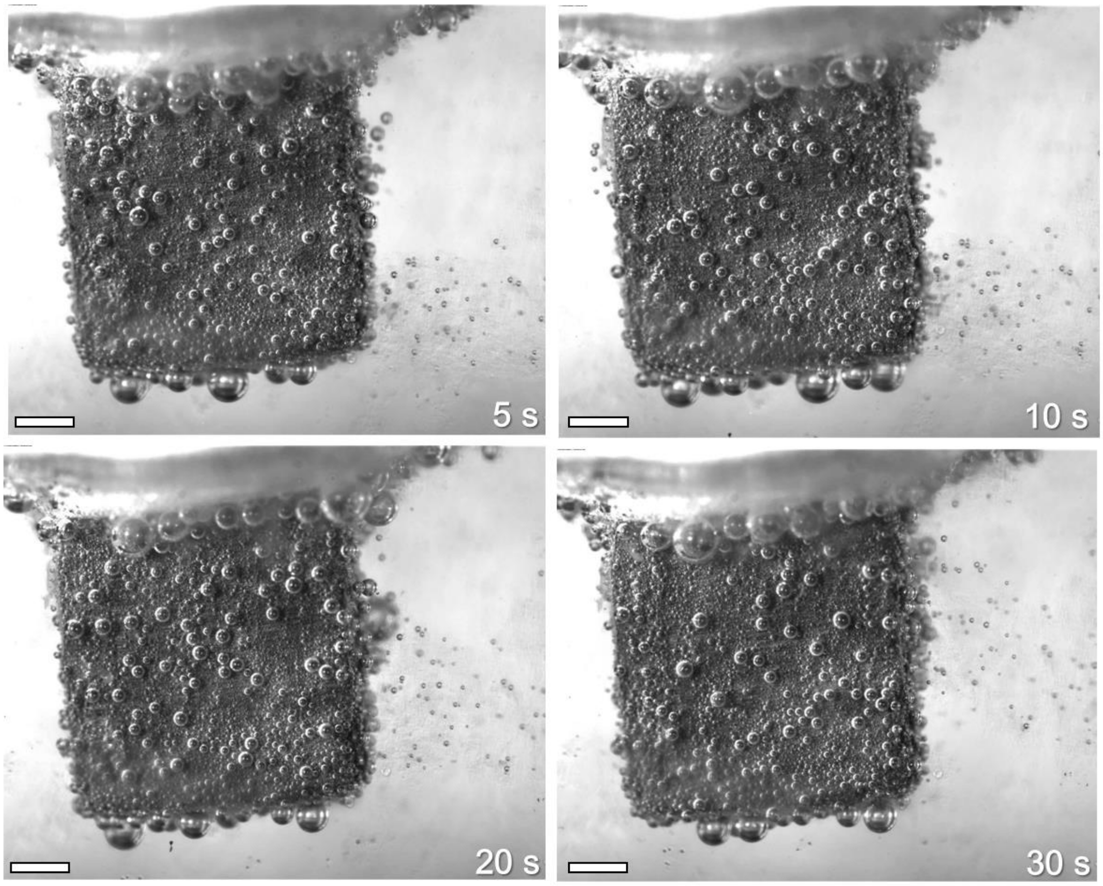

Supplementary Fig. 48 | Movements of H_2 bubbles and precipitates during the eNSR process on the front of the Ni_2P/PC cathode, captured in real time by a high-speed camera. Scale bars: 1 mm.

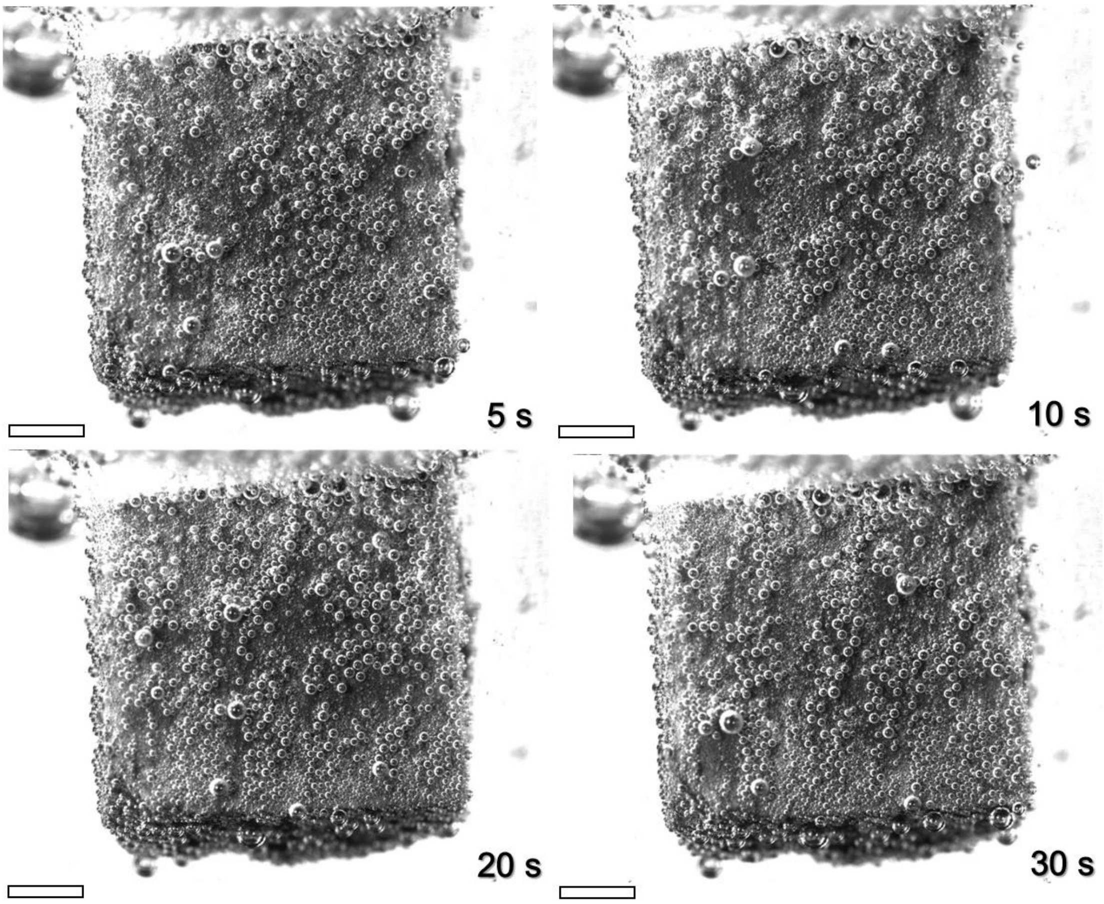

Supplementary Fig. 49 | Movements of H_2 bubbles and precipitates during the eNSR process on the front of the MoS_2/PC cathode, captured in real time by a high-speed camera. Scale bars: 1 mm.

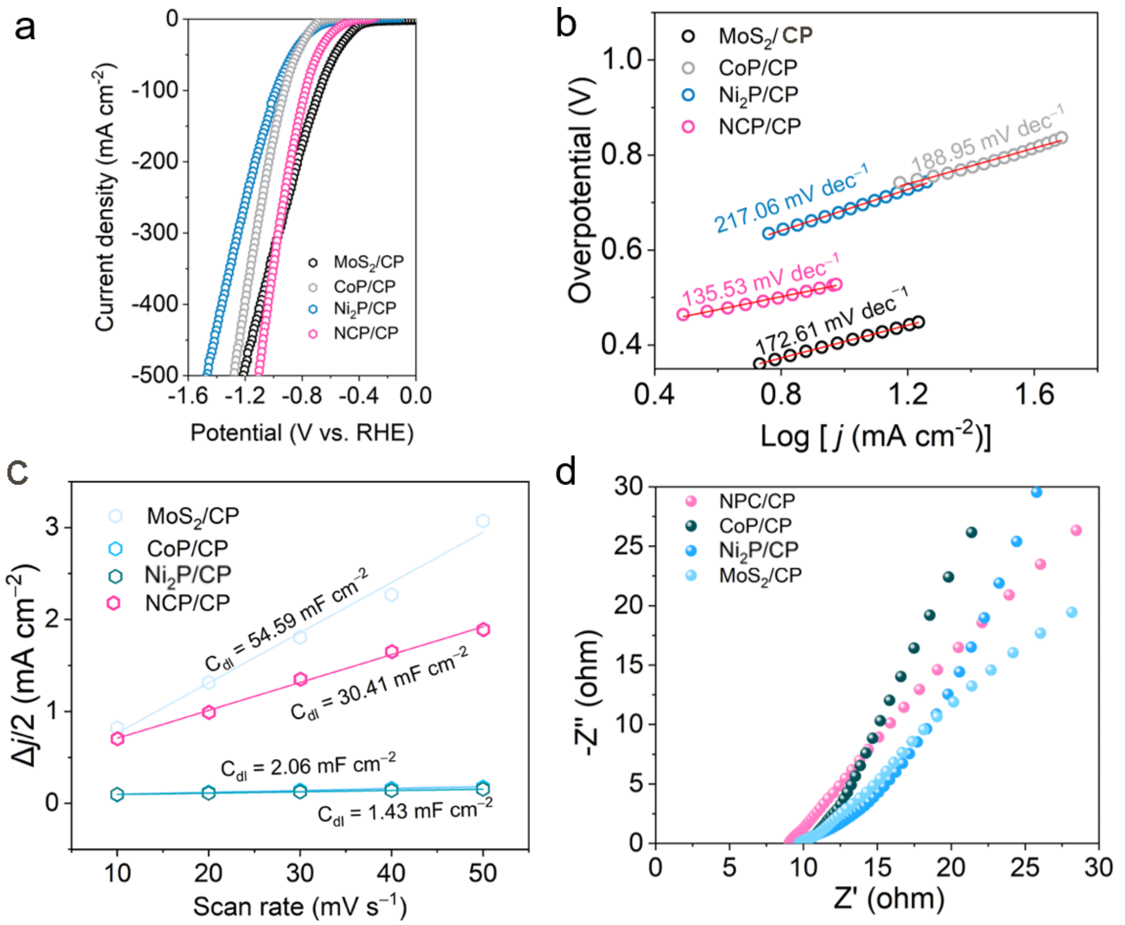

Supplementary Fig. 50 | Comparison of eNSR activities of single NCP, CoP, Ni_2P and MoS_2 supported by CP. (a) Linear sweep voltammetry curves, (b) Tafel slopes, (c) C_{dl} values, and (d) Nyquist plots of different PC-based electrodes in natural seawater, including NCP/CP, Ni_2P/CP, CoP/CP, and MoS_2/CP.

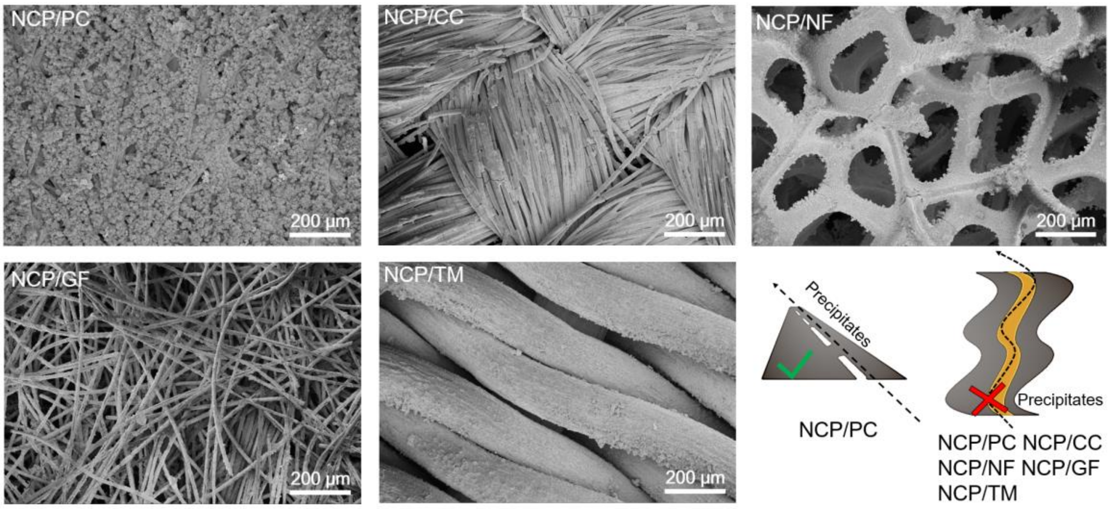

Supplementary Fig. 51 | Low-magnification SEM images presenting the broader view of NCP-based cathodes that possess poor anti-precipitation ability for eNSR.

In the natural seawater electroreduction, precipitates and H_2 bubbles are theoretically generated almost simultaneously because the formation of precipitation must consume OH^-, a byproduct from the process of generating H_2 bubbles. Ca^{2+}/Mg^{2+} precipitates do not actively detach from the electrodes when they form; they are not buoyant like air bubbles. Therefore, to solve the challenge of precipitates adhering to the cathode during H_2 production from seawater, it is extremely promising to consider bubbles as natural nano/micron-cleaners so that the local flows can dislodge these precipitates in a timely manner. However, precipitation does not bypass obstacles as bubbles/water generally do, and if there are too many twists and turns in the process of precipitate following or being swept by bubble flows (and by forces generated by bubble bursting and merging), the precipitation will be confined to the obstacles. Therefore, the cathode should not only provide the forces/flows to repel Ca^{2+}/Mg^{2+} precipitates but also the paths for Ca^{2+}/Mg^{2+} precipitates to move away from the electrode with the bubbles. Our wood-derived 3D cathode has the architecture with abundant, relatively straight, regular, and nature-made channels, which are not available in other substrates such as PC, CC, NF, GF, and TM.

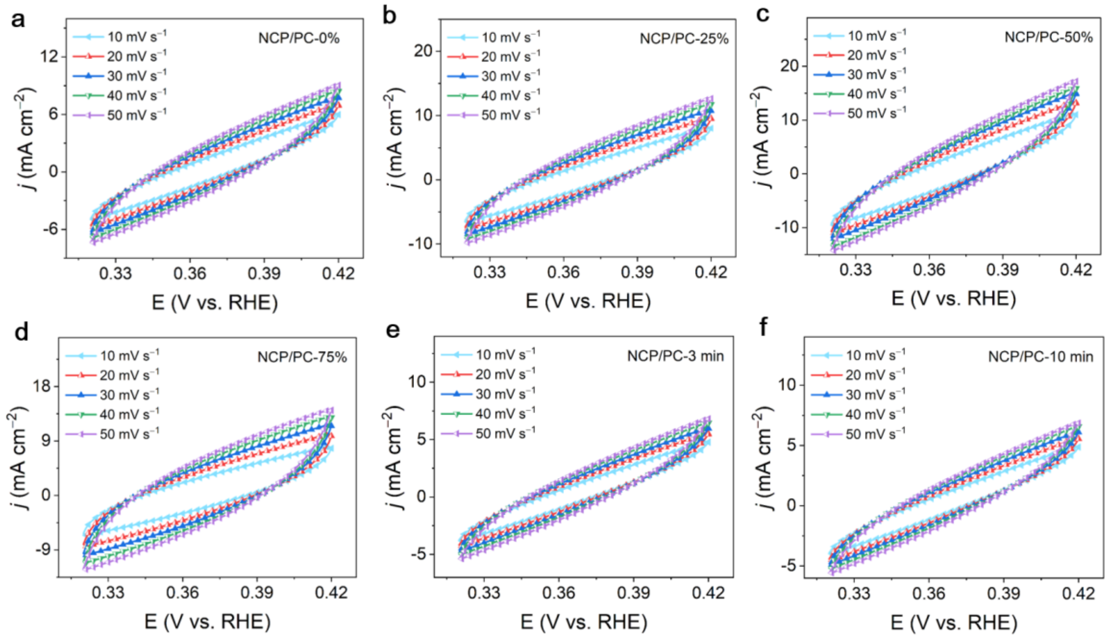

Supplementary Fig. 52 | Electrochemical C_{dl} measurement results for NCP/PC with different pore blocking treatments, including (a) NCP/PC-0%, (b) NCP/PC-25%, (c) NCP/PC-50%, (d) NCP/PC-75%, (e) NCP/PC-3 min, and (f) NCP/PC-10 min.

In order to block the pore structure of NCP/PC to varying degrees, we immersed NCP/PC in an aqueous solution with the same concentration of agarose gel at varied depths (a same soaking time). To be more specific, NCP/PC being immersed 1/4 of its volume will lose 25% of the pore structure (75% of the available pores are thus left, denoted as NCP/PC-75%), NCP/PC being immersed 1/2 of its volume will lose 50% of the pore structure (denoted as NCP/PC-50%), NCP/PC being immersed 3/4 of its volume will lose 75% of the pore structure (denoted as NCP/PC-25%), and NCP/PC being fully immersed in agarose gel-containing solution will lose 100% of the pore structure (denoted as NCP/PC-0%). Meanwhile, two more samples with extended soaking times include NCP/PC-3 min and NCP/PC-10 min.

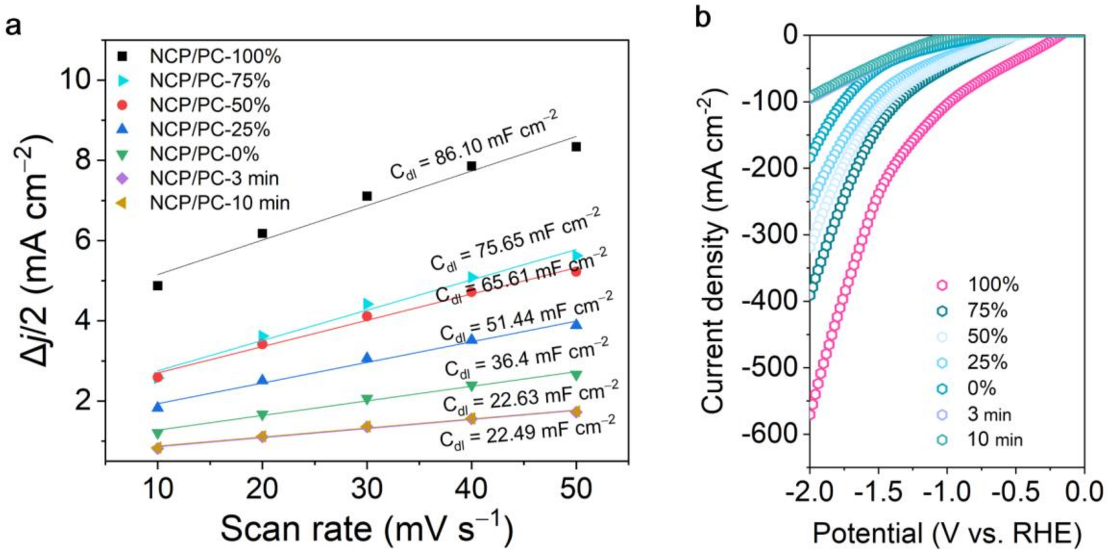

Supplementary Fig. 53 | (a) C_{dl} values and (b) the corresponding changes in eNSR *j* versus the applied potentials for NCP/PC with different pore blocking treatments.

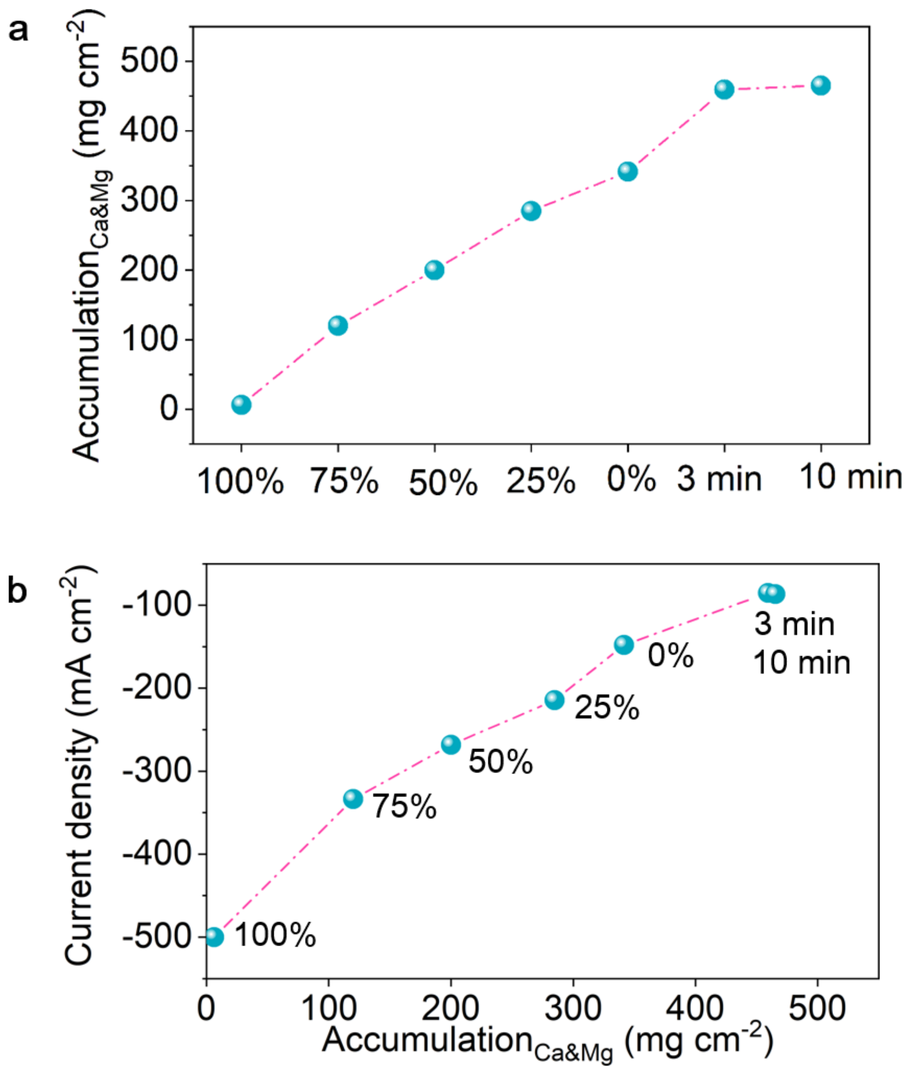

Supplementary Fig. 54 | (a) Accumulation amounts of Mg and Ca for various cathodes. (b) Linear fitting of *j* and precipitation amounts for NCP/PC with different pore blocking treatments. Note that *j* here indirectly represent whether the bubble release is effective or not.

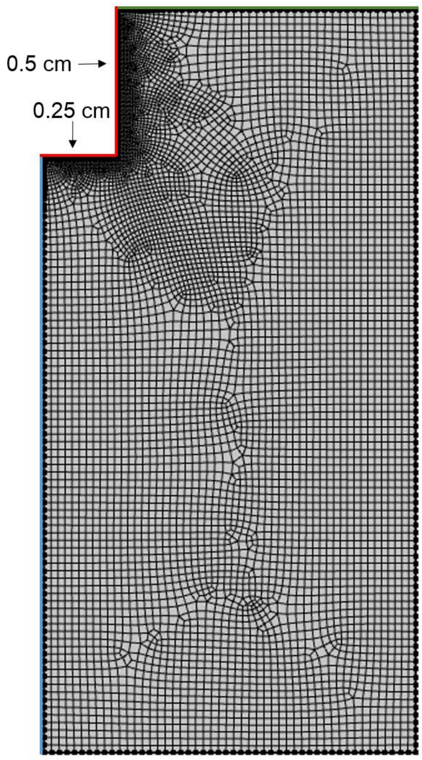

Supplementary Fig. 55 | Compute domain of simulation 1.

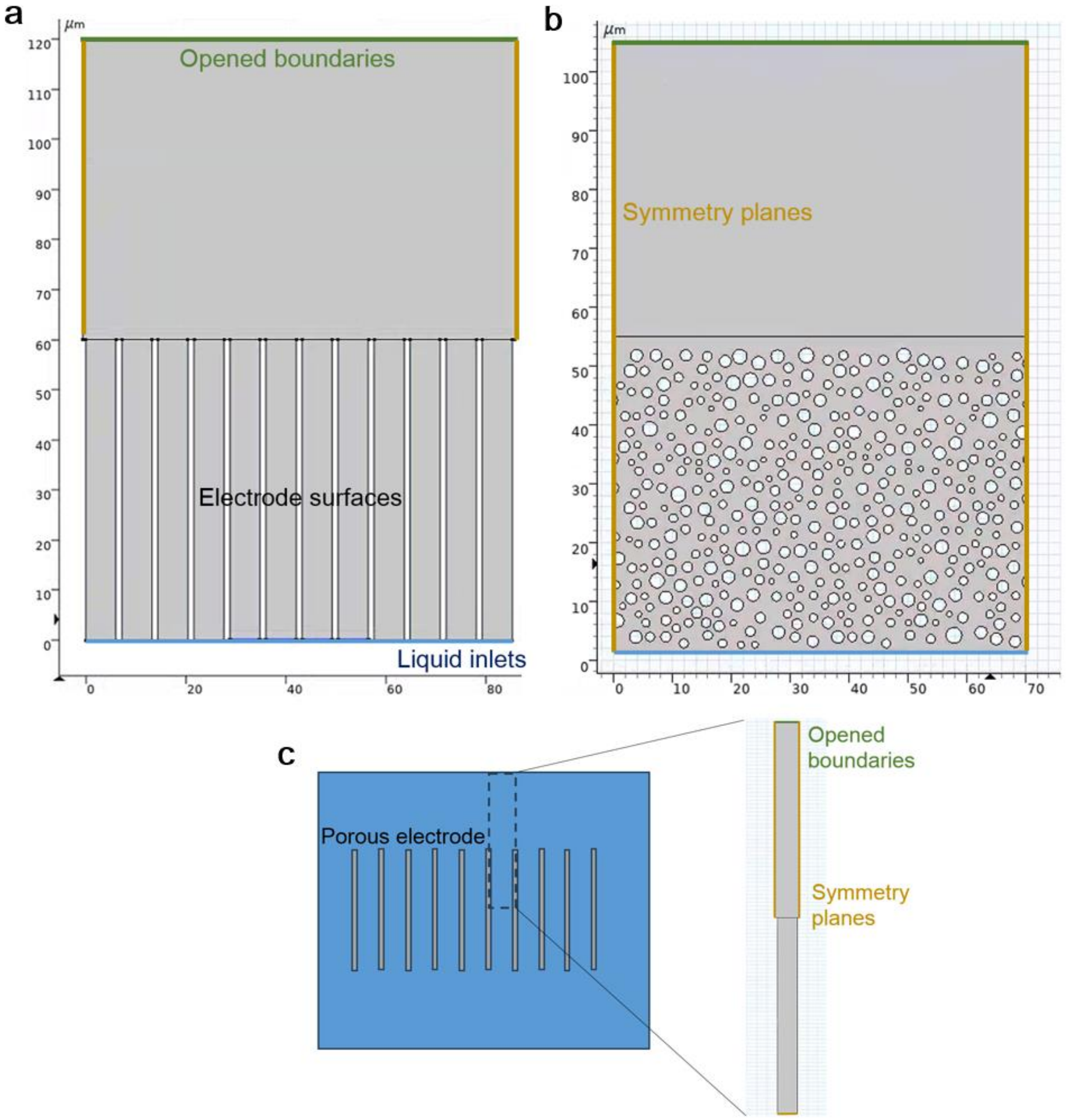

Supplementary Fig. 56 | Compute domains and different boundary conditions in (a, b) simulations for the model of bubble rise outside the electrode and the model of bubble-based anti-precipitation mechanism in the electrode and (c) simulations for pore size optimization of the optimal sample electrode.

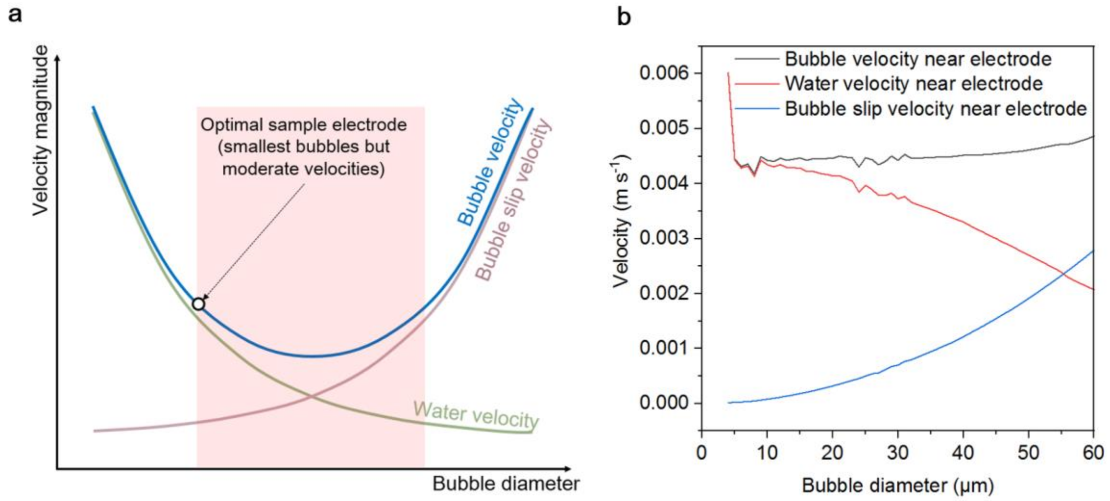

Supplementary Fig. 57 | (a) A brief schematic diagram of the velocity magnitude versus the bubble diameter. (b) Relationship between the stabilized velocity of the liquid phase (u_{gas}, time-averaged from t = 30 s to 50 s) and the bubble diameter (i_0 = 0.125 A).

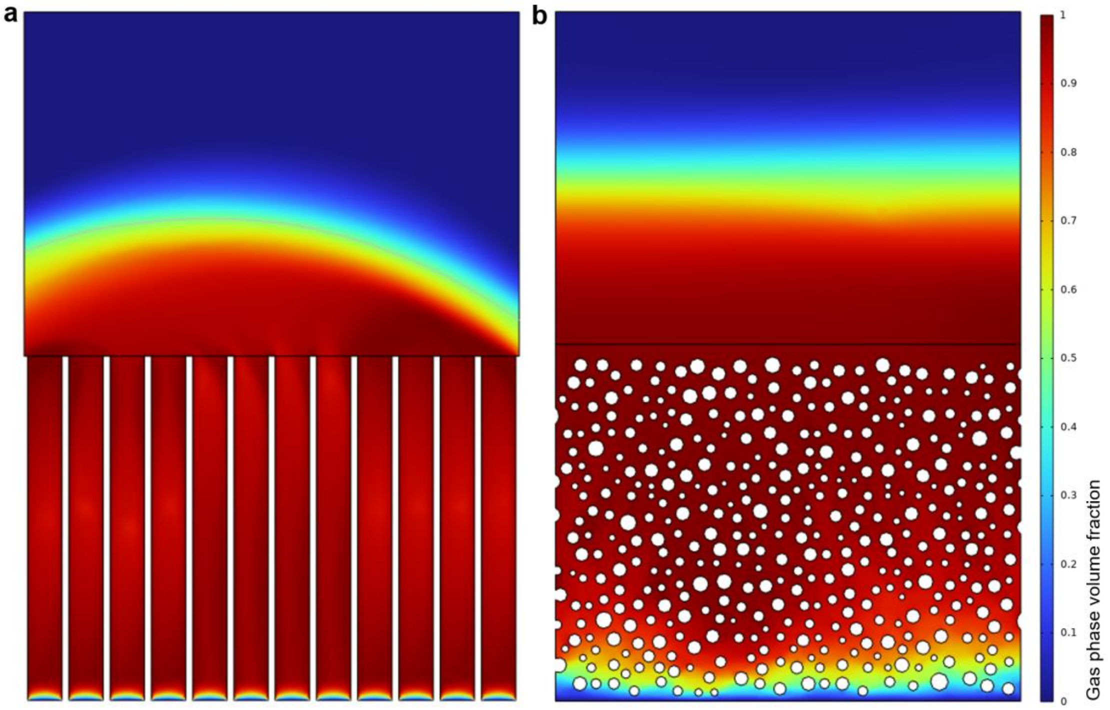

Supplementary Fig. 58 | Quantitative comparison of the volume fraction of gas phase, the ϕ_g, for (a) the optimal electrode and (b) the counterpart electrode.

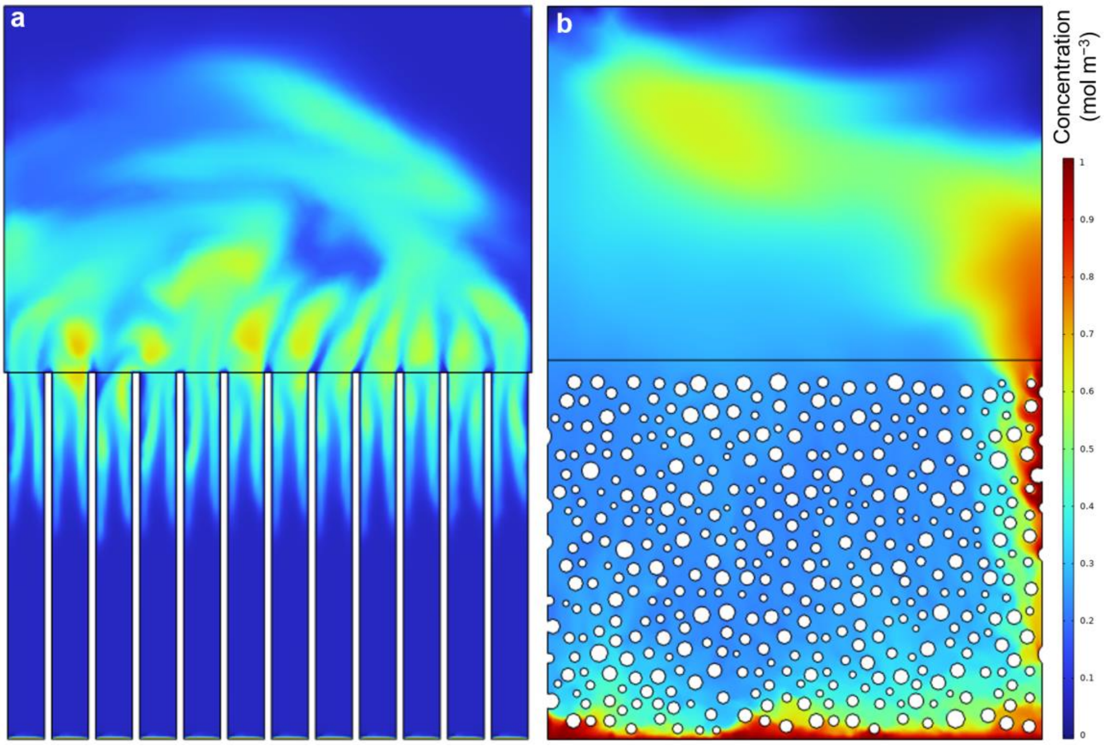

Supplementary Fig. 59 | Precipitation concentration distribution of (a) the optimal electrode that represents NCP/PC and (b) the counterpart electrode.

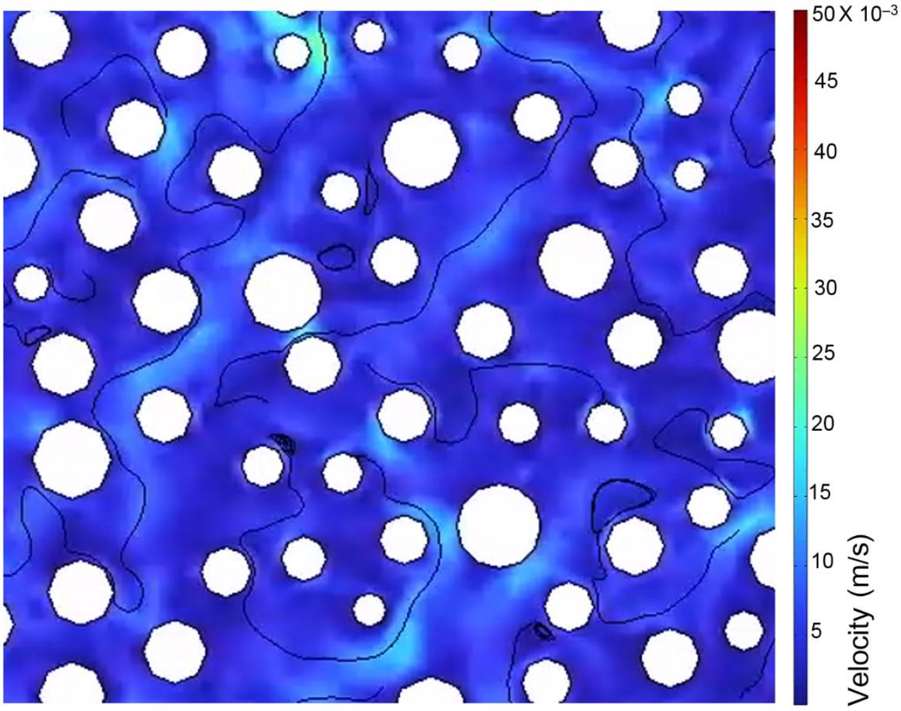

Supplementary Fig. 60 | Vortices that are prone to appear in the velocity field. The non-directional flow field is not conducive to sediment repulsion.

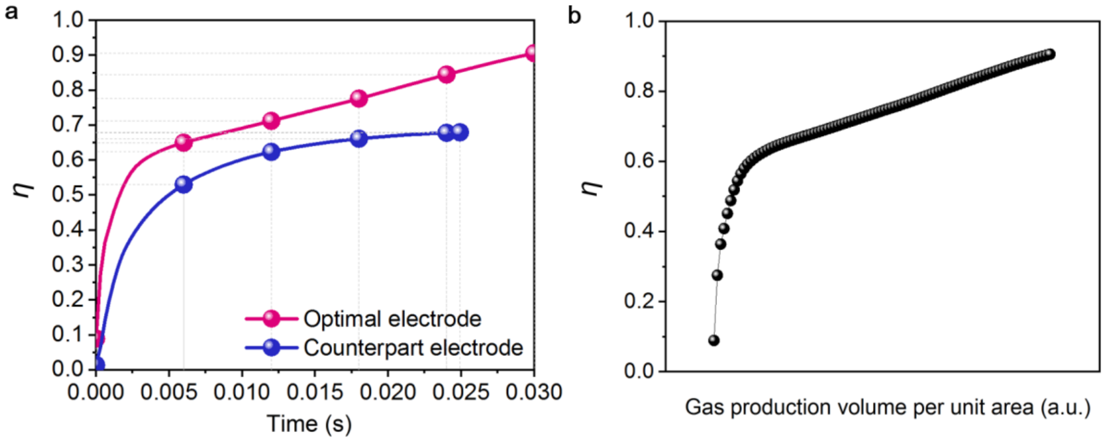

Supplementary Fig. 61 | (a) Quantitative comparison of the optimal electrode and the counterpart electrode in terms of the η(t). (b) The relationship between the η and the gas production volume per unit area.

The bubble release volume per electrode area can be obtained from the equation (12) in Supplementary Note 6 (v = v_1t), and the relationship between repelling rate and gas production volume per unit area can be obtained by converting η(t) to η(v). The precipitate concentration inside the electrode (below the black line in the middle, please see Supplementary Fig. 58 and 59) at the initial moment is set as c_0 = 1 mol m^{-3}, and the concentration outside the electrode is 0 mol/m^3. We defined the repelling rate as:

$$\eta(t) = 1 - \frac{1}{c_0 V}\left(\int_{\Omega} c(t)\, dV + A\right)$$

where Ω refers to the portion of the computational domain within the electrode, and A is the amount of precipitated material from time 0 to time t. Comparison of the repelling rate requires a given characteristic time τ, its scale is equivalent to the period of seawater injection (i.e., both are equal in size by orders of magnitude), and empirically preferable τ = 0.001s to 0.1s. Repelling rates at time t = τ quantitatively reflects the abilities of the electrodes to repel precipitates.

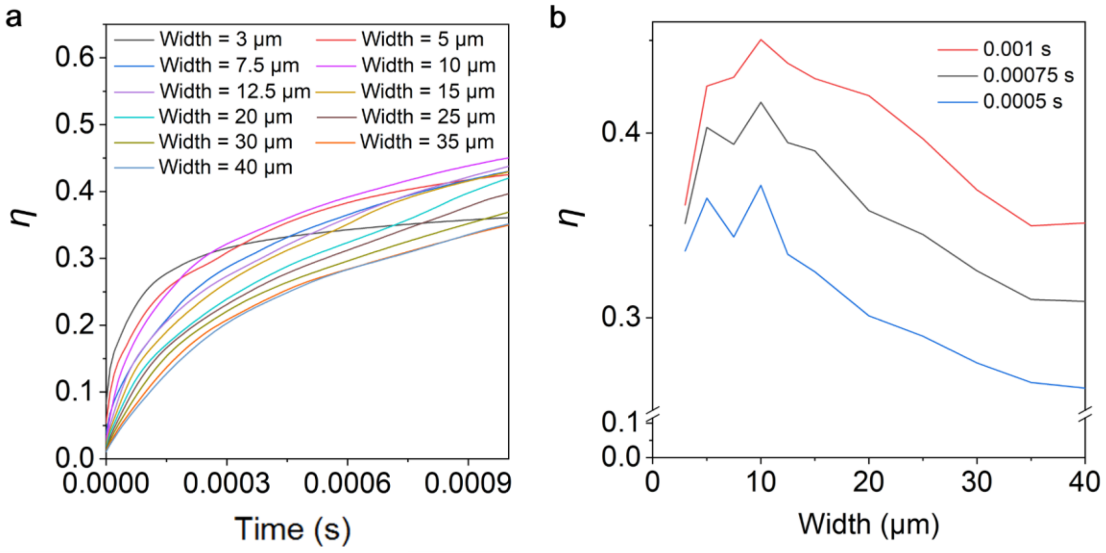

Supplementary Fig. 62 | (a) Quantitative comparison of *η* for different pore widths. (b) Determination of the optimal pore width.

This model (of pore size optimization) is calculated in similar ways as the models of bubble rise and bubble-based anti-precipitation mechanisms (Supplementary Notes 5 and 6). When the characteristic time is short, smaller holes (with lower pore widths) are more dominant/advantageous because they can fill up with bubbles faster, and the optimal pore size increases with the increase of the characteristic time, and the optimal pore size is near 10 um in the interval of t = 0.0005 to 0.001s under this condition.

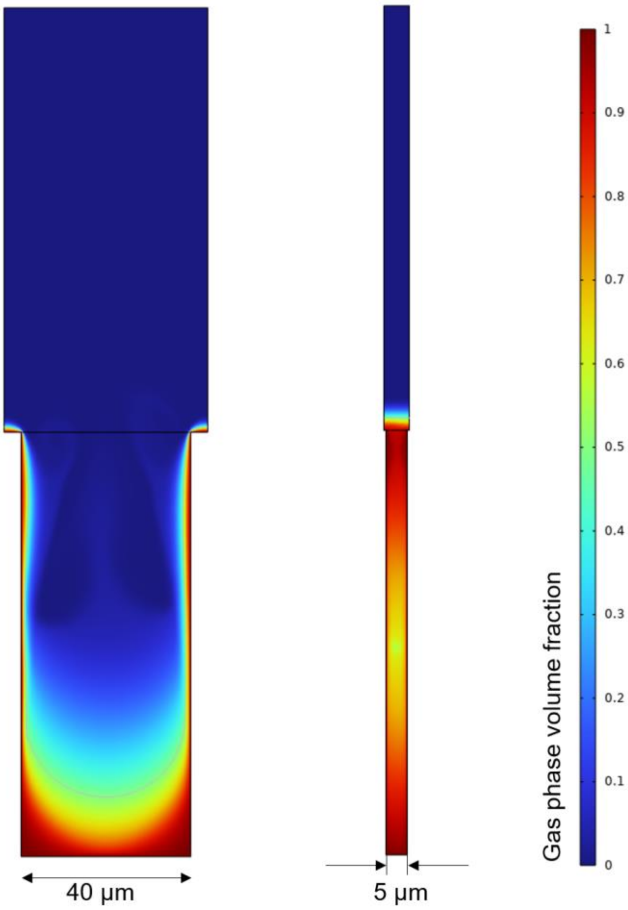

Supplementary Fig. 63 | Quantitative comparison of the gas phase distribution in pores of different widths.

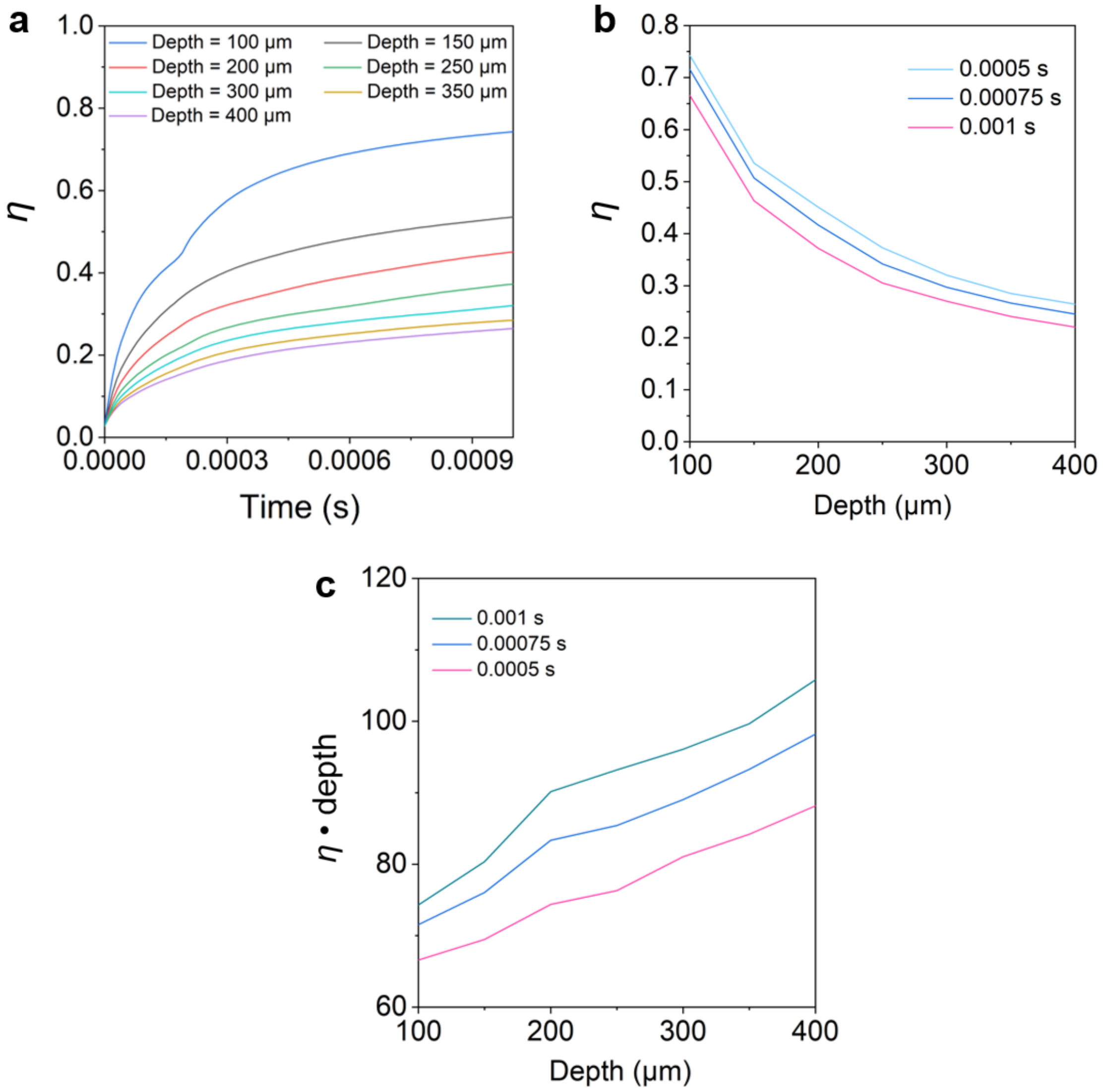

Supplementary Fig. 64 | (a) Relationship between the repelling ratio and the time at different pore depths. (b) Relationship between the repelling ratio and the pore depth at different times. (c) Comparison of the performance at different times by multiplying the repelling ratio and the depth of the pore.

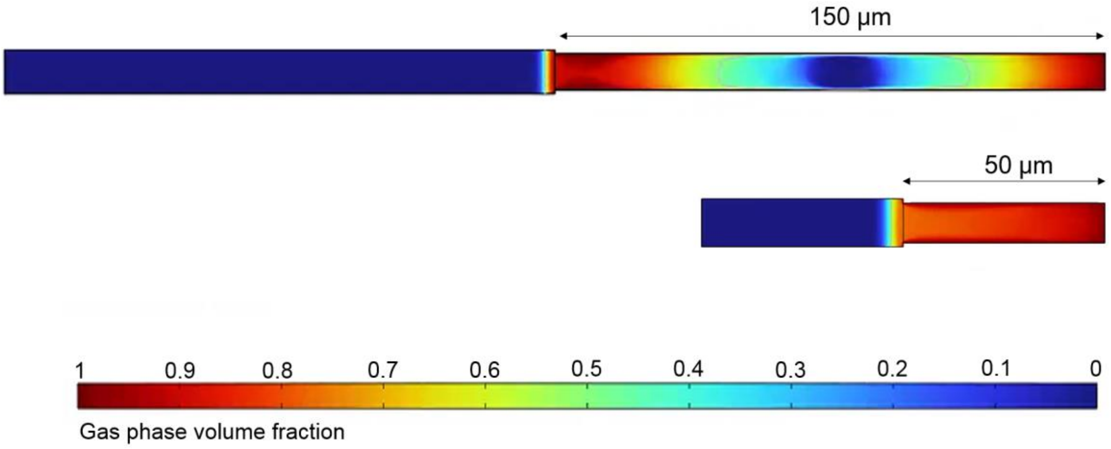

Supplementary Fig. 65 | Quantitative comparison of the gas phase distribution in pores of different depths.

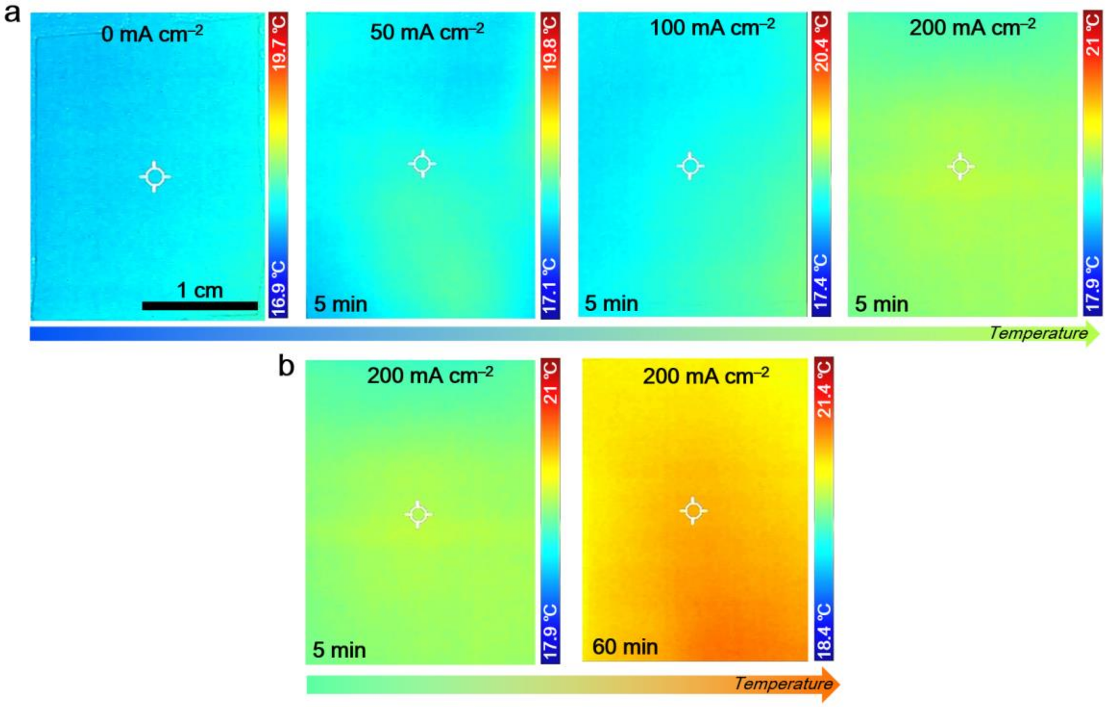

Supplementary Fig. 66 | Mapping eNSR activities (current densities) on our NCP/PC electrode (size: ~2 cm ×~3 cm) using infrared thermography. (a) Mapping eNSR activities under different current densities as well as (b) different reaction times. Since the experiments were conducted in winter, the temperatures were lower.

We performed this measurement in a three-electrode system. The quantity of heat (q) generated on the catalyst surface as a result of charge transfer is linearly correlated with the current density ($q \propto j$). For an individual electrode region, the occurrence of localized activity should result in proportional local heat generation. Thus, the observation of the localized heating distribution can in principle be regarded as an electrochemical activity distribution (please refer to: *ACS Energy Lett.* **7**, 2410–2419 (2022), *Nat. Commun.* **14**, 6579 (2023)). The heat map is directly related to the current distribution. At the reduction j of 200 mA cm^{-2}, the relatively uniform heat map is equivalent to a relatively uniform current density distribution on the electrode surface (for instance, please see the 5-min sample or 60-min sample in Supplementary Fig. 66b).

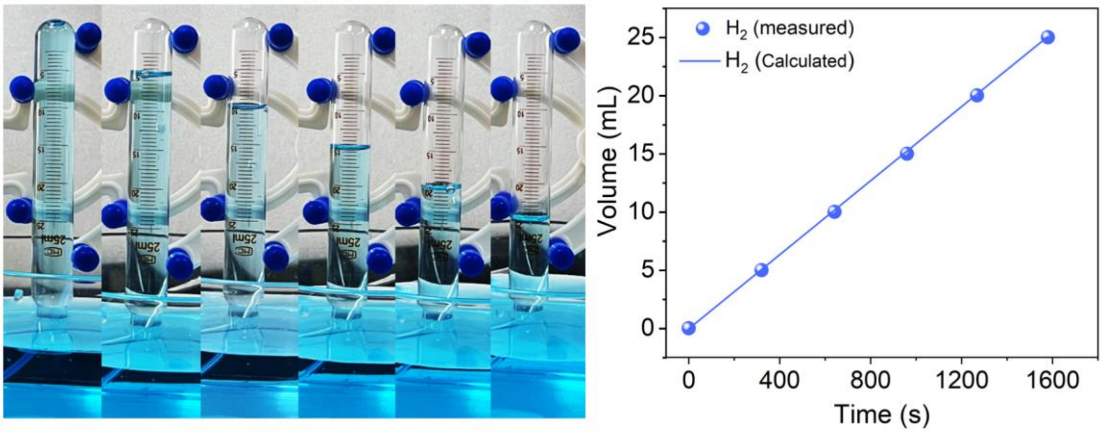

Supplementary Fig. 67 | Optical photographs of the drainage tests and the related produced gas amounts for Faradaic efficiency calculations.

We conducted the gas quantification tests at regular intervals and determined the each FE value in the selected range (25-min testing for each measurement). The H_2 gas produced from the cathodic chamber will be fed into the drainage tube.

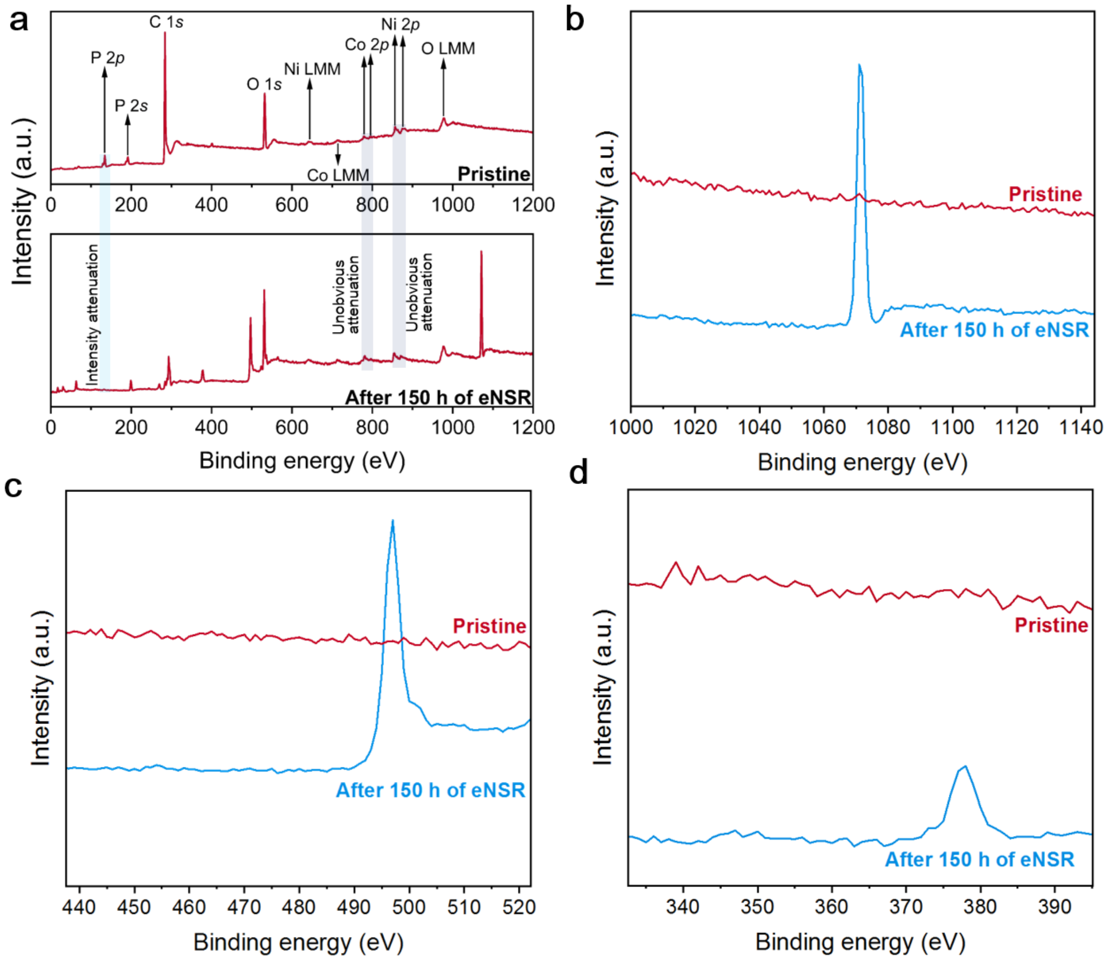

Supplementary Fig. 68 | XPS survey scan analysis based on data before and after the natural seawater electrolysis. (a) Full range spectra. (b–d) Evidence of Na/K species from seawater left on the surface of the electrode after the test.

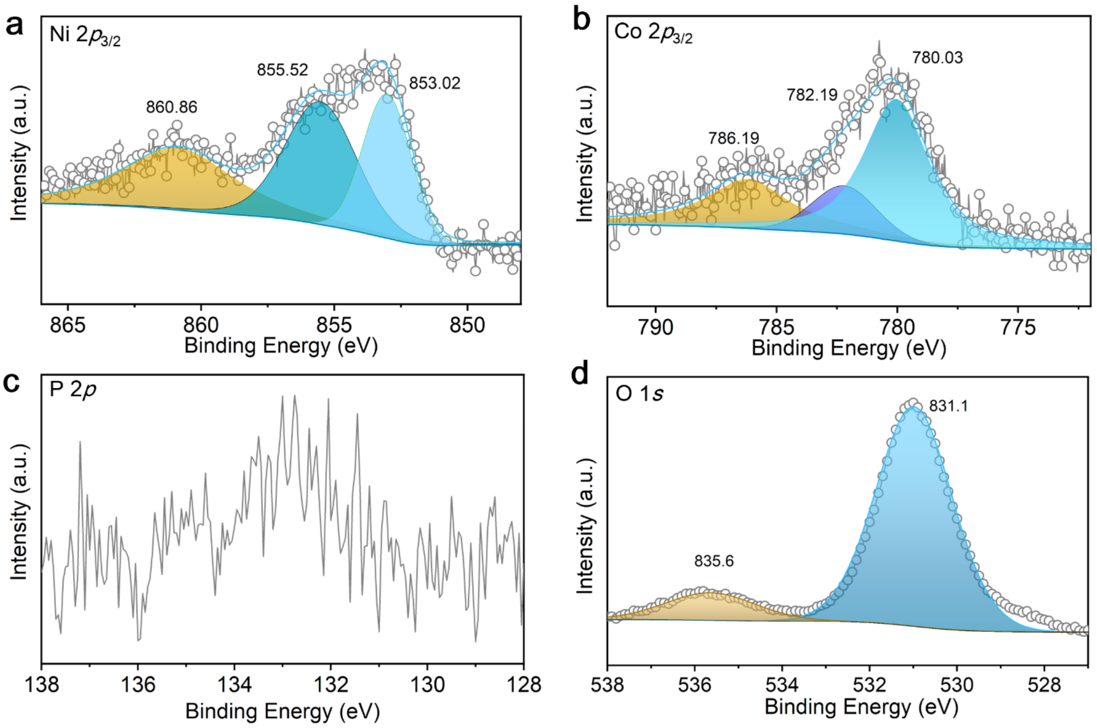

Supplementary Fig. 69 | High-resolution XPS spectra of the NCP/PC after the long-term natural seawater reduction tests in the (a) Ni 2*p*, (b) Co 2*p*, (c) P 2*p*, and (d) O 1*s* regions.

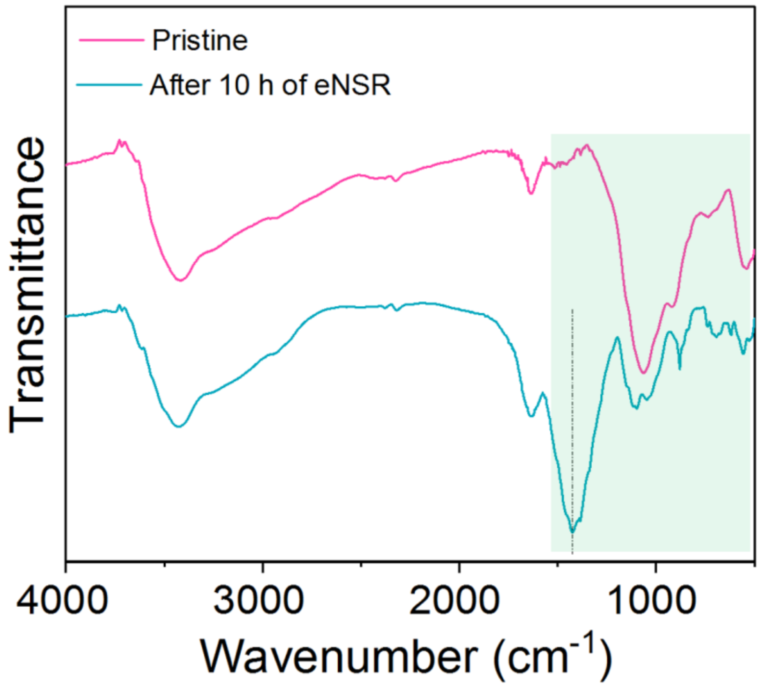

Supplementary Fig. 70 | Infrared signals of NCP/PC in the range of 500–4000 cm^{-1} before and after the natural seawater reduction test. The most obvious change occurs at wavenumbers lower than 1425 cm^{-1} (bands below 800 cm^{-1} may correspond to metal–oxygen stretching and bending modes, please refer to: *J. Mater. Chem. A* **1**, 9046–9053 (2013)), as part of the phosphides convert to NiCo-based oxidized species (please refer to: *J. Colloid Interface Sci.* **608**, 70–78 (2022), *Chinese Chem. Lett.* **33**, 2741–2746 (2022), *Ceram. Int.* **40**, 5339–5342 (2014), *Energy Fuels* **35**, 18815–18823 (2021), *Adv. Funct. Mater.* **17**, 644–650 (2007), *Sci. Rep.* **6**, 18737 (2016), *Int. J. Hydrogen Energy* **36**, 10057–10064, (2011)).

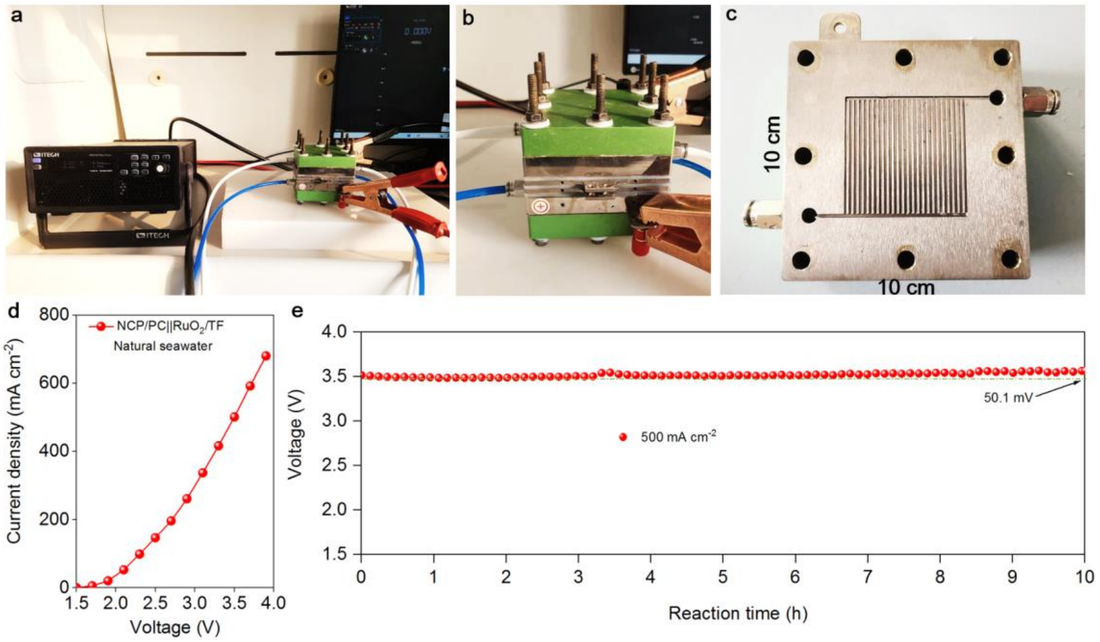

Supplementary Fig. 71 | Performance measurements of the NCP/PC cathode in an electrolyzer consisting of single stacks. (a–c) Pictures of the DC power as well as the outside and inside of the scaled-up electrolyzer for industrially relevant testing. The total electrode area is 54 cm^2. (d) Polarization curve of the electrolyzer by measuring the voltage at a series of current densities (without *iR* compensation). (e) Long-term durability data. Notes: TF stands for porous Ti felt.

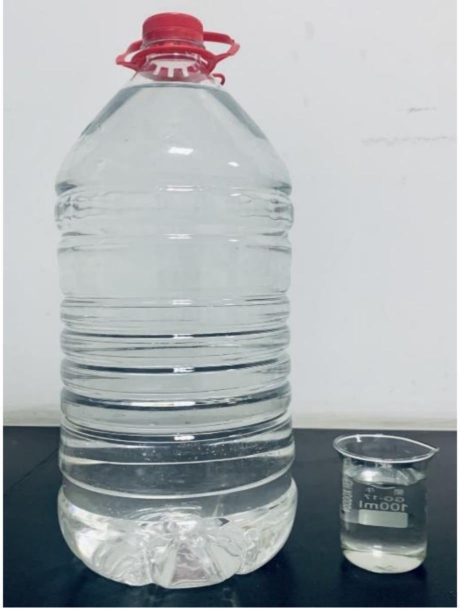

Supplementary Fig. 72 | Natural seawater directly collected from the Yellow Sea, China. Note that the seawater can be used directly as the electrolyte for eNSR measurements in this study without disinfection or filtration.

Supplementary Table 1. Pore structure data of our 3D PC substrate by both BET and MIP characterizations.

Data	PC
BET surface area	685.0335 $m^2\ g^{-1}$
Single point adsorption total pore volume of pores	0.303044 $cm^3\ g^{-1}$ (pores less than 346.1816 nm diameter)
Single point adsorption total pore volume of pores less than 40.3122 nm diameter	0.300800 $cm^3\ g^{-1}$
Desorption average pore diameter	5.0962 nm
MIP porosity	50.1493%
Interstitial porosity	47.6300 %
Median Pore Diameter (Volume)	10347.0 nm
Median Pore Diameter (Area)	6.8 nm
Average Pore Diameter (4V/A)	610.2 nm

Supplementary Table 2. Comparison of Tafel slopes for the NCP/PC with some previously reported electrocatalysts in alkaline seawater.

Catalyst	Electrolyte	Tafel slopes	Ref.
NCP/PC	1 M KOH + seawater	40.93 mV dec^{-1}	This work
CeO_2/α-MoC/β-Mo_2C MRs/CC	1 M KOH + seawater	58.4 mV dec^{-1}	*Appl. Catal. B* **317**, 121774 (2022)
F-$FeCoP_v$@IF	1 M KOH + seawater	108 mV dec^{-1}	*Appl. Catal. B* ***328***, 122487 *(2023)*
Ru-FeP_4/IF	1 M KOH + seawater	51.58 mV dec^{-1}	*Appl. Catal. B* **319**, 121950 (2022)
NiS@FeNiP/NF	1 M KOH + seawater	61.9 mV dec^{-1}	*Small* **19**, 2300194 (2023)
MnCo/NiSe/NF	1 M KOH + seawater	51.24 mV dec^{-1}	*Appl. Catal. B* **325**, 122355 (2023)
RuFe-Ni_2P@NF	1 M KOH + seawater	97 mV dec^{-1}	*Nano Energy* **105**, 108008 (2023)
RuNi-Fe_2O_3/IF	1 M KOH + seawater	85.08 mV dec^{-1}	*Chin. J. Catal.* **43**, 2202–2211 (2022)
Fe-CoNiP@NC	1 M KOH + seawater	86.09 mV dec^{-1}	*Chem. Eng. J.* **446**, 136987 (2022)

Notes: IF stands for iron foam.

Supplementary Table 3. Comparison of electrochemical stability at different reduction current densities for the NCP/PC with some previously reported electrocatalysts in alkaline seawater.

Catalyst	Electrolyte	*j* (mA cm^{-2})	Time (h)	Ref.
NCP/PC	1 M KOH + seawater	1000	1000	This work
CeO_2/α-MoC/β-Mo_2C MRs/CC	1 M KOH + seawater	1000	100	*Appl. Catal. B* **317**, 121774 (2022)
Ni-MoN/Cu foam	1 M KOH + seawater	500 (two-electrode system)	100 (two-electrode system)	*Adv. Mater.* **34**, 2201774 (2022)
MnCo/NiSe/NF	1 M KOH + seawater	500	200	*Appl. Catal. B* **325**, 122355 (2023)
NiMoN/NF	1 M KOH + seawater	500 (two-electrode system)	100 (two-electrode system)	*Nat. Commun.* **10**, 5106 (2019)
Ni_2P-Fe_2P/NF	1 M KOH + seawater	500	24	*Adv. Funct. Mater.* **31**, 2006484 (2021)
$Co_2Mo_3O_8$/MnO_2/NF	1 M KOH + seawater	100	100	*Appl. Catal. B* **338**, 123015 (2023)
Mo_2C-Ru/C	1 M KOH + seawater	100	100	*Adv. Funct. Mater.* **34**, 2301925 (2023)
Ni-WO_x@NF	1 M KOH + seawater	10	120	*Appl. Catal. B* **325**, 122397 (2023)
Ni-SA/NC	1 M KOH + seawater	50	14	*Adv. Mater.* **33**, 2003846 (2021)
Pt/CNT-$_{200}$$(N_2H_5)_4Mo_2S_6$	1 M KOH + seawater	1000	600	*Appl. Catal. B* **338**, 122996 (2023)
Ni-NiO-Cr_2O_3	1 M KOH + seawater	400 (two-electrode system)	1000 (two-electrode system)	*Proc. Natl. Acad. Sci.* **116**, 6624–6629 (2019)
$(Fe_{0.74}Co_{0.26})_2P/Ni_3N$	1 M KOH + seawater	100	40	*Small* **19**, 2207082 (2023)
NiMoS/NF	1 M KOH + seawater	800 (two-electrode system)	100 (two-electrode system)	*Appl. Catal. B* **291**, 120071 (2021)
FeP@CoP/NF	1 M KOH + seawater	~175 (two-electrode system)	100 (two-electrode system)	*Appl. Catal. B* **317**, 121799 (2022)
$Fe_2P/Ni_{1.5}Co_{1.5}N/Ni_2P$	1 M KOH + seawater	100	40	*ACS Nano* **17**, 1681 (2023)
HW-NiMoN-2h	1 M KOH + seawater	1000	70	*Nano-Micro Lett.* **15**, 157 (2023)
FeP@CoP/CC	1 M KOH + seawater	10	100	*Nano Energy* **114**, 108601 (2023)
Cr-Co_xP/NF	1 M KOH +	100	140	*Adv. Funct. Mater.* **33**,

	seawater			2214081 (2023)
Ni-SN@C	1 M KOH + seawater	11	40	*Adv. Mater.* **33**, 2007508 (2021)
R-CoC_2O_4@MXene	1 M KOH + seawater	10	100	*Nat. Commun.* **13**, 5785 (2022)
Rh/RhOOH metallene/GCE	1 M KOH + seawater	10	20	*Adv. Funct. Mater.* **32**, 2201081 (2022)
Pt-Co-Mo/NF	1 M KOH + seawater	500	12	*Appl. Catal. B* **317**, 121762 (2022)
NiMoN/NF	1 M KOH + seawater	500 (two-electrode system)	100 (two-electrode system)	*Energy Environ. Sci.* **13**, 3439 (2020)
Fe/F-Ni_2P@NC/NF	1 M KOH + seawater	100	30	*Chem. Eng. J.* **454**, 140210 (2023)
$Ru_{1,n}$-$ZnFe_2O_x$-C/GDE	1 M KOH + seawater	10	150	*Small* **18**, 2204155 (2022)
PC@CMS-NG	1 M KOH + seawater	50 (two-electrode system)	37 (two-electrode system)	*Chem. Eng. J.* **473**, 145348 (2023)
$Fe_{0.01}$-Ni&$Ni_{0.2}Mo_{0.8}N$/NF	1 M KOH + seawater	425 (two-electrode system)	80 (two-electrode system)	*Energy Environ. Sci.* **15**, 3945 (2022)
FMCO/NF	1 M KOH + seawater	10 (two-electrode system)	72 (two-electrode system)	*Appl. Catal. B* **328**, 122488 (2023)
RuNi-Fe_2O_3/IF	1 M KOH + seawater	100	24	*Chin. J. Catal.* **43**, 2202–2211 (2022)
Fe-CoNiP@NC	1 M KOH + seawater	~100	24	*Chem. Eng. J.* **446**, 136987 (2022)
Cu_2S/NF	1 M KOH + seawater	~15	24	*Int. J. Hydrog. Energy* **47**, 30819–30829 (2021)
Pt-Ni@NiMoN/NF	1 M KOH + seawater	500	40	*Energy Environ. Sci.* **16**, 4584–4592 (2023)

Notes: GCE stands for glassy carbon electrode.

Supplementary Table 4. Comparison of overpotentials at a high reduction current density of –1000 mA cm^{-2}, η_{1000}, for the NCP/PC with some previously reported electrocatalysts in (simulated) alkaline seawater.

Catalyst	Electrolyte	η_{1000} (mV)	Ref.
NCP/PC	1 M KOH + seawater	145	This work
NCP/PC	1 M KOH + seawater	η_{500} = 121	This work
CeO_2/α-MoC/β-Mo_2C MRs/CC	1 M KOH + seawater	165	*Appl. Catal. B* **317**, 121774 (2022)
Ni-MoN/Cu foam	1 M KOH + seawater	176	*Adv. Mater.* **34**, 2201774 (2022)
F-$FeCoP_v$@IF	1 M KOH + seawater	210	*Appl. Catal. B* **328**, 122487 (2023)
$Ni_2P/Co(PO_3)_2$/NF	1 M KOH + seawater	307	*Int. J. Hydrogen Energy* **48**, 17783 (2023)
$1T_{0.63}$-$MoSe_2$@MoP	1 M KOH + seawater	317	*ACS Appl. Mater. Interfaces* **14**, 30683 (2022)
Ru-FeP_4/IF	1 M KOH + seawater	318	*Appl. Catal. B* **319**, 121950 (2022)
NiS@FeNiP/NF	1 M KOH + seawater	327	*Small* **19**, 2300194 (2023)
Ru-Ni(Fe)P_2/NF	1 M KOH + seawater	375	*Small* **19**, 2300030 (2023)
Cu_3P-FeP@CC	1 M KOH + 0.5 M NaCl	406	*ACS Appl. Energy Mater.* **5**, 2909 (2022)
P-Ni_4Mo/CF	1 M KOH + seawater	551	*Chin. J. Struct. Chem.* **41**, 2207068 (2022)
MnCo/NiSe/NF	1 M KOH + seawater	270	*Appl. Catal. B* **325**, 122355 (2023)
NiMoN/NF	1 M KOH + 0.5 M NaCl	218	*Nat. Commun.* **10**, 5106 (2019)
Ni_2P-Fe_2P/NF	1 M KOH + seawater	389	*Adv. Funct. Mater.* **31**, 2006484 (2021)
RuFe-Ni_2P@NF	1 M KOH + seawater	310	*Nano Energy* **105**, 108008 (2023)
NiPS/NF	1 M KOH + seawater	η_{500} = ~275	*J. Energy Chem.* **75**, 66–73 (2022)
RuNi-Fe_2O_3/IF	1 M KOH + seawater	η_{500} = 300	*Chin. J. Catal.* **43**, 2202–2211 (2022)
NiCoHPi@Ni_3N/NF	1 M KOH + seawater	η_{500} = 281	*ACS Appl. Mater. Interfaces* **14**, 22061–22070 (2022)
Ni_3N@C/NF	1 M KOH + seawater	η_{500} = 279	*J. Mater. Chem. A* **9**, 13562–13569 (2021)
NiCo@C/MXene/Cu foam	1 M KOH + seawater	η_{500} = 275	*Nat. Commun.* **12**, 4182 (2021)
CoO@C/MXene/NF	1 M KOH + seawater	η_{500} = 270	*Adv. Mater.* **34**, e2109321 (2022)

Fe-CoNiP@NC	1 M KOH + seawater	η_{500} = 260	*Chem. Eng. J.* **446**, 136987 (2022)
FeP@CoP/NF	1 M KOH + seawater	η_{500} = 450	*Appl. Catal. B* **317**, 121799 (2022)

Notes: Some reported electrodes do not achieve ampere-level current densities, and we also give the corresponding overpotentials at an eASR current density of –500 mA cm^{-2}, η_{500}, for these previous electrodes and our NCP/PC electrode.

Supplementary Table 5. C_{dl}, mass activity values, and η for various NCP-based cathodes in natural seawater.

Electrode	C_{dl}	mass activity	η
NCP/PC	86.1 mF cm^{-2}	26.733 A mg^{-1}@−0.5 V_{RHE}	553.8 mV at 500 mA cm^{-2}
NCP/NF	72.1 mF cm^{-2}	2.689 A mg^{-1}@−0.5 V_{RHE}	874 mV at 500 mA cm^{-2}
NCP/TM	68.6 mF cm^{-2}	3.459 A mg^{-1}@−0.5 V_{RHE}	961.4 mV at 500 mA cm^{-2}
NCP/CC	35.5 mF cm^{-2}	4.855 A mg^{-1}@−0.5 V_{RHE}	1116.4 mV at 500 mA cm^{-2}
NCP/GF	55.2 mF cm^{-2}	2.348 A mg^{-1}@−0.5 V_{RHE}	992.6 mV at 500 mA cm^{-2}
NCP/CP	30.4 mF cm^{-2}	4.322 A mg^{-1}@−0.5 V_{RHE}	1213.3 mV at 500 mA cm^{-2}

Supplementary Table 6. Comparison of R_{ct} of various cathodes, including NCP/CP, NCP/NF, NCP/CC, NCP/TM, NCP/GF, and NCP/PC. EIS data were collected in the frequency range of 0.1 to 500000 Hz.

Cathode	R_{ct} in natural seawater (ohm)
NCP/CP	17.3
NCP/GF	16.8
NCP/CC	15.5
NCP/TM	14.2
NCP/NF	12.2
NCP/PC	9.0

Supplementary Table 7. Comparison of stability at different current densities for the two-electrode system in natural seawater.

Catalyst	Electrolyte	*j* (mA cm^{-2})	Time (h)	Ref.
NCP/PC‖DSA	Natural seawater	500	150	This work
Cr_2O_3–CoO_x@Ti fibre felt‖Cr_2O_3–CoO_x@Ti fibre felt	Natural seawater	500	100	*Nat Energy* **8**, 264–272 (2023)
NiCoN|Ni_xP|NiCoN‖S-(Ni,Fe)OOH	Natural seawater	10 (with notable voltage loss)	24	*ACS Energy Lett.* **5**, 2681–2689 (2020)

Supplementary Table 8. Ion/elemental compositions and pH values of the natural seawater (before seawater alkalization) and alkaline seawater (after seawater alkalization) used in this work.

Species	Conc. [mg L^{-1}] for natural seawater	Conc. [mg L^{-1}] for alkaline seawater
Mg^{2+}	1016.751	0.16
Ca^{2+}	358.04	8.371
K^{+}	358.04	35123.26
SO_4^{2-}	658.86	664.689
Na^{+}	9877.836	11492.33

Notes: The seawater was collected from Huangdao district, Qingdao city, China (in summer). The pH value for natural seawater was around 7.89, and pH for alkaline seawater was around 14.
