# Supplementary material for: Efficient bubble/precipitate traffic enables stable seawater reduction electrocatalysis at industrial-level current densities
Source: Nat Commun. 2024 Apr 5;15:2950. doi: 10.1038/s41467-024-47121-x (PMC10997793; doi:10.1038/s41467-024-47121-x)
Supplement: Supplementary file 3 — Description of Additional Supplementary Files [file 41467_2024_47121_MOESM3_ESM.pdf]

DESCRIPTION OF ADDITIONAL SUPPLEMENTARY FILES

File name: Supplementary Movie 1

Description: Real-time observation of the bubble release process of NCP/PC.

File name: Supplementary Movie 2

Description: Real-time observation of the bubble release process of NCP/NF.

File name: Supplementary Movie 3

Description: Real-time observation of the bubble release process of NCP/TM.

File name: Supplementary Movie 4

Description: Real-time observation of the bubble release process of NCP/CC.

File name: Supplementary Movie 5

Description: Real-time observation of the bubble release process of NCP/GF.

File name: Supplementary Movie 6

Description: Real-time observation of the bubble release process of NCP/CP.
